# Asymmetric synthesis of chiral cycloalkenone derivatives *via* palladium catalysis†
†Electronic supplementary information (ESI) available: Detailed experimental procedures and copies of NMR spectra and HPLC chromatograms. See DOI: 10.1039/c3sc53250j



**DOI:** 10.1039/c3sc53250j

**Published:** 2014-01-27

**Authors:** Barry M. Trost, James T. Masters, Jean-Philip Lumb, Dahlia Fateen

**Affiliations:** a Department of Chemistry , Stanford University , Stanford , CA 94305-4401 , USA . Email: bmtrost@stanford.edu

## Abstract

The palladium-catalyzed oxidative desymmetrization of *meso*-dibenzoates yields γ-benzoyloxy cycloalkenones in good yields and with excellent levels of enantioselectivity. These compounds serve as precursors to a broad range of substituted cycloalkenones, including well-established synthetic building blocks and elaborated cycloalkanone derivatives. The ability to prepare both enantiomers of the oxidative desymmetrization products enables a unified strategy toward stereochemically diverse epoxyquinoid natural products.

## Abstract 2

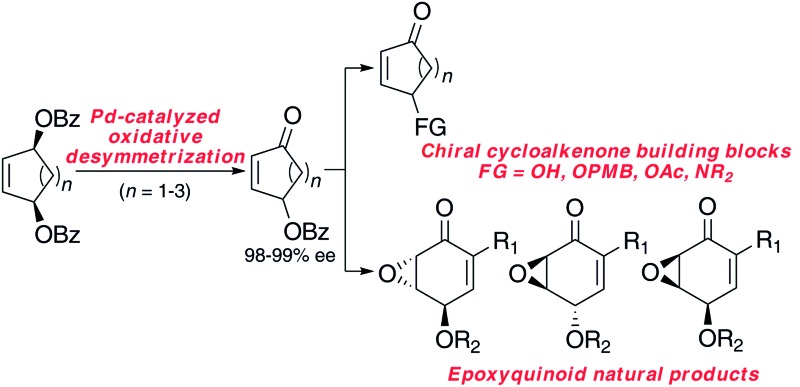
Palladium-catalyzed oxidative desymmetrization enables the efficient synthesis of both enantioenriched cycloalkenone building blocks and diverse epoxyquinoid natural products.

## Introduction

**Fig. 1:**
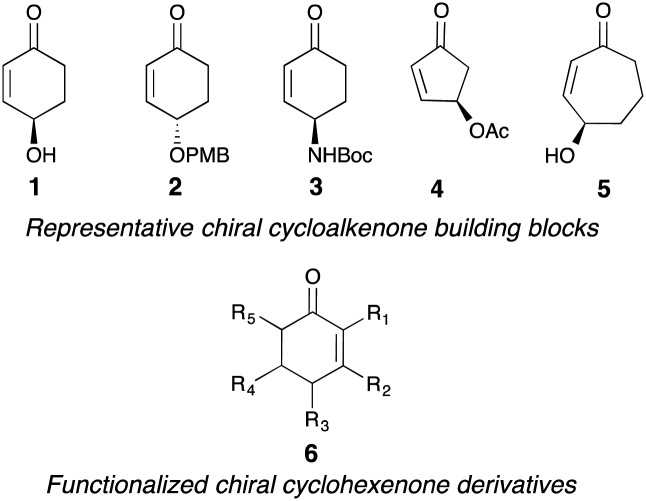
Diverse chiral cycloalkenones.

Chiral cycloalkenones are prominent structural motifs in organic synthesis.^1^ Chiral γ-substituted cycloalkenones in particular are important building blocks in both natural product and pharmaceutical synthesis. Heteroatom-substituted cycloalkenones such as **1–5** (Fig. 1) have been used extensively in these endeavors.^2^ Chemoselective elaboration of these building blocks may also provide densely substituted products in which every carbon is differentially functionalized. This is exemplified by cyclohexenone derivative **6**, a general structure that encompasses a number of natural products.

A number of syntheses of such chiral cycloalkenones have been reported, many of which involve enzymatic processes or multi-step derivations of chiral pool materials.^3^ These well-established approaches can provide products with very high levels of enantioenrichment, but they may not permit access to both product enantiomers without requiring lengthier or otherwise less practical synthetic sequences. This consideration is of particular importance given that both enantiomers of certain building blocks (*e.g.*, **1** and *ent*-**1**) have been employed in synthesis.^4^

Asymmetric catalysis may present an opportunity to prepare both enantiomers of a target with equal facility through catalyst control. These preparations may also benefit from high atom and step economy.^5,6^ Several innovative, catalytic asymmetric syntheses of γ-substituted cycloalkenones have been described.^7^ These methods often target or perform best for a specific cycloalkenone ring size or type of γ-substituent. A general method for the synthesis of chiral cycloalkenones of various ring sizes and with different γ-substituents would complement these approaches.

Herein we report such a strategy using asymmetric palladium catalysis. This process affords enantioenriched cyclopentenones, cyclohexenones, and cycloheptenones bearing various heteroatom substituents at the γ-position. This protocol also enables the efficient, asymmetric synthesis of more densely functionalized cyclohexenone-derived natural products.

**Fig. 2:**
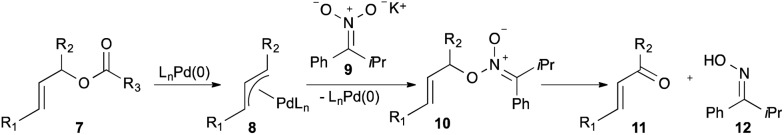
Pd-catalyzed allylic oxidation.

Our group has demonstrated that palladium-catalyzed allylic alkylation presents a unique method for the oxidation of allylic esters (Fig. 2).^8^ In this process, ionization of allylic ester **7** generates π-allylpalladium intermediate **8**, which undergoes selective *O*-alkylation with nitronate **9** to yield **10**. Fragmentation then provides α,β-unsaturated product **11** and oxime **12**, which can be recycled to **9**.^9^

**Fig. 3:**
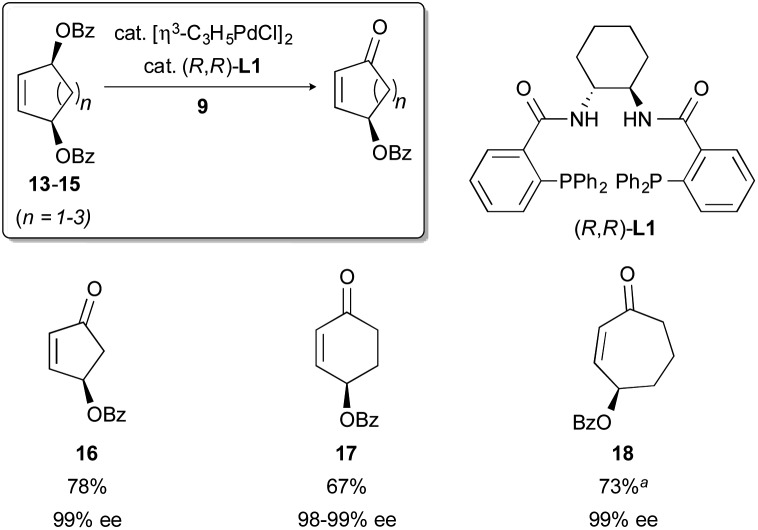
Oxidative desymmetrization of *meso* electrophiles. (*a*) Yield at 68% conversion (b.r.s.m.).

*Meso*-1,4-allylic dibenzoates are excellent substrates for this process. When compounds **13–15** (Fig. 3) are subjected to the reaction conditions in the presence of our chiral ligand **L1**, an oxidative desymmetrization proceeds. This delivers γ-benzoyloxy cycloalkenones **16–18** in good yields and with excellent enantioselectivity. Importantly, either enantiomer of the desired product can be obtained from the same *meso* precursor simply by selecting either (*R*,*R*)- or (*S*,*S*)-**L1**.

**Fig. 4:**
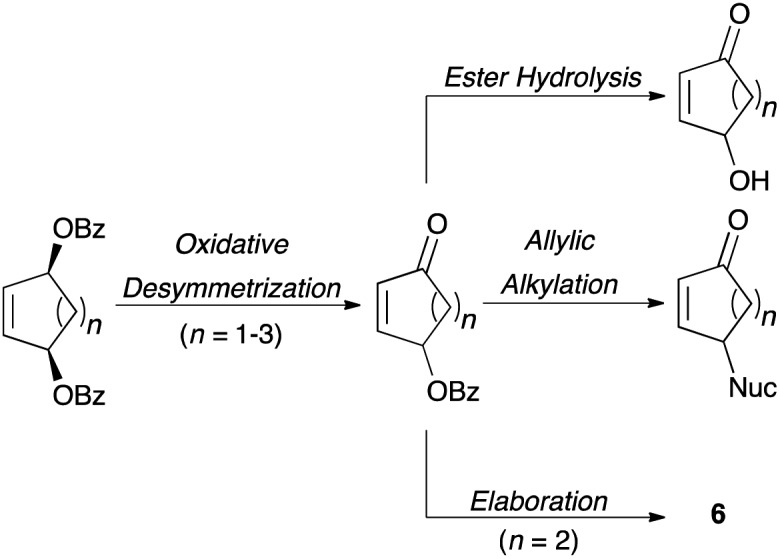
Strategy for the synthesis of diverse cycloalkenone derivatives.

With the aforementioned building blocks and natural product motifs serving as motivation, we sought to expand upon this entry into chiral γ-substituted cycloalkenones. We envisioned pairing this oxidative desymmetrization with the palladium-catalyzed allylic alkylation of heteroatom nucleophiles. This would enable access to a diverse set of γ-substituted cycloalkenones (Fig. 4). Complementing this, ester hydrolysis would provide γ-hydroxy cycloalkenones.

We also anticipated that the γ-substituents of the cycloalkenones so produced would direct the diastereoselectivity of subsequent intra- and intermolecular reactions involving the enone moiety. This would enable the rapid assembly of molecular complexity with excellent stereocontrol. Further manipulations would install additional functionality at the remaining positions, leading to fully elaborated structures of the type **6**.

## Results and discussion

In pursuit of these goals, it was necessary to perform the synthesis of cyclohexenone **17** from *meso*-diester **14** on larger (multi-gram) scale. It was soon discovered that the isolated yields on such a scale were highly variable and generally lower than yields on smaller scale. This was the case even when the consumption of **14** was high (TLC). Contemporaneously, other operations involving **17** revealed its sensitivity toward aqueous or alcoholic base. Suspecting that such a base (*e.g.*, KOH, from the KH used to generate nitronate **9**) may have been the cause of the lowered yields on larger scale, we instituted several operational modifications to improve the robustness of the oxidative desymmetrization reaction. Specifically, in the preparation of **9**, the commercial KH dispersion in mineral oil was washed with THF, rather than the more typical hexanes, to remove not only the oil but also any KOH present in the material. In addition, the reactions were limited in duration while monitoring for the consumption of the *meso*-dibenzoate (TLC). Once the dibenzoate was fully or largely consumed, the reaction mixture was thoroughly quenched with pH 7 buffer prior to extractive workup.

**Fig. 5:**
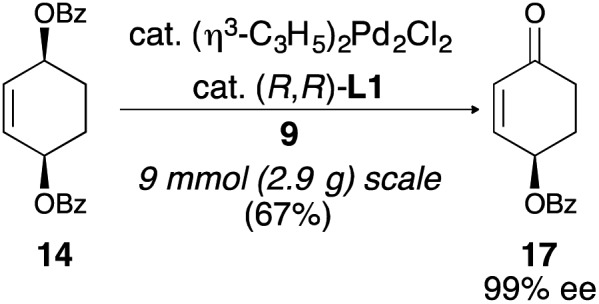
Multi-gram scale synthesis of **17**.

Validation of these modifications came in the form of increased and more consistent reaction yields. For example, the oxidation of **14** → **17** proceeded in a reproducible *ca*. 67% yield on multi-gram scale (9 mmol, a 35-fold increase in scale from our original report) while maintaining excellent enantioselectivity (Fig. 5). Applying this modified protocol to **13** and **15** was similarly successful.^10^

The palladium-catalyzed allylic alkylation (Pd-AA) of cycloalkenones **16–18** with heteroatom nucleophiles proved highly effective.^11^ Chiral γ-substituted cyclopentenone, cyclohexenone, and cycloheptenone products were obtained in good yields and with very good to excellent levels of enantioselectivity (Table 1). Alkylation with carboxylic, phenolic, and alcoholic nucleophiles successfully delivered oxygenated building blocks *ent*-**2** and **4** as well as products **19–21**. This synthesis of *ent*-**2** is particularly significant, as alkyl alcohols are typically poor nucleophiles in palladium-catalyzed allylic alkylation.^11*a*^ The preparation of acetoxyenone **19** is also noteworthy, as our attempts to engage the diacetate analogue of **14** in the oxidative desymmetrization were unsuccessful. Thus, this strategy of Pd-catalyzed oxidative desymmetrization and Pd-catalyzed transesterification represents a new and useful solution to this limitation. Alkylation with nitrogen nucleophiles was also successful, with potassium phthalimide as well as alkyl amines reacting smoothly to provide products **22–25**.

**Table 1:** Scope of Pd-catalyzed allylic alkylation (Pd-AA) of **16–18** ^*a*^

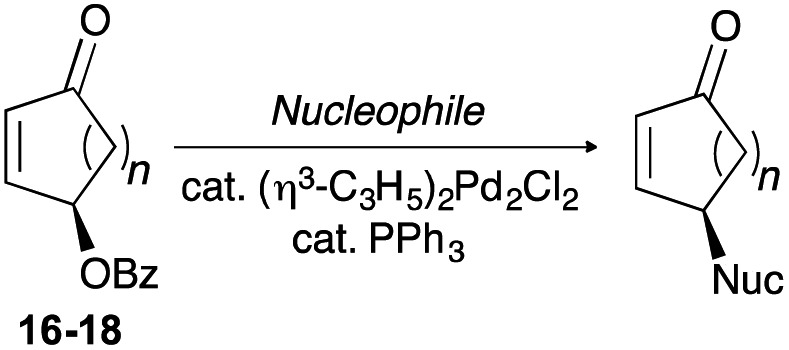				
Nucleophile	Electrophile	Product	Yield [%]	ee^*b*^ [%]
NaOAc	**16**	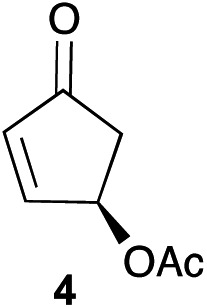	64^*c*^	93
NaOAc	**17**	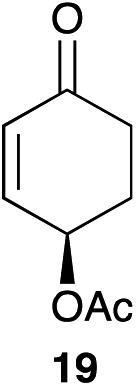	75	96
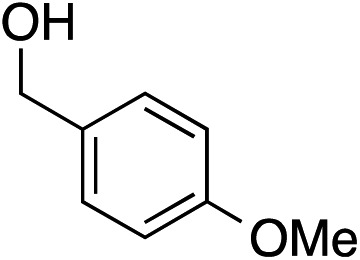	**17**	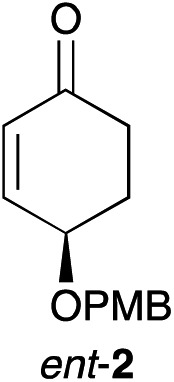	62	91
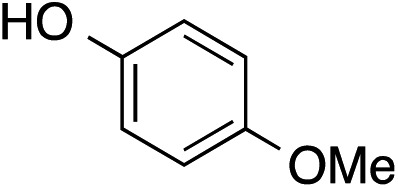	**17**	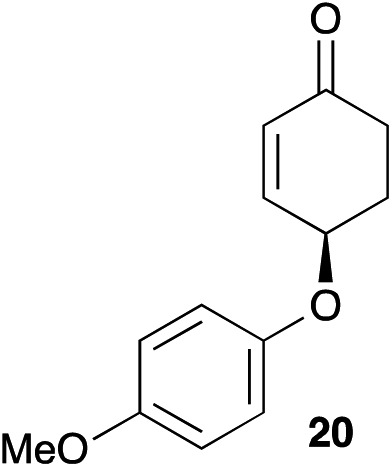	80^*d*^	98
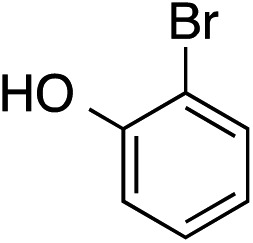	**17**	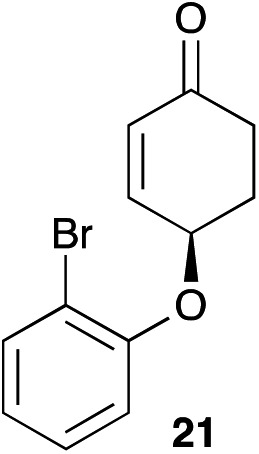	66^*d*^	92
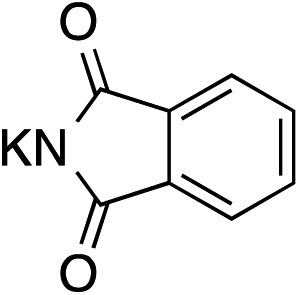	**17**	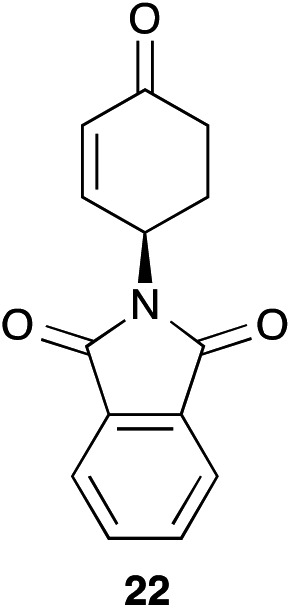	69	99
HN(*n*Pr_2_)	**17**	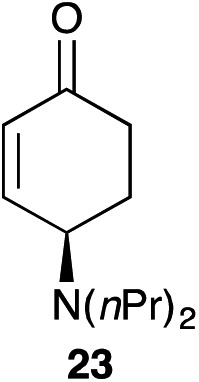	69	99
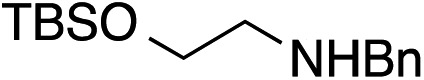	**17**	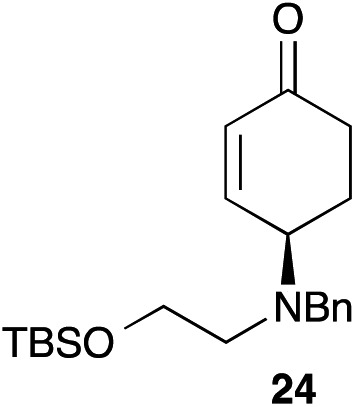	85	87
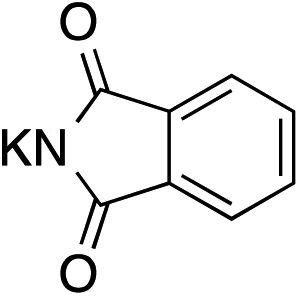	**18**	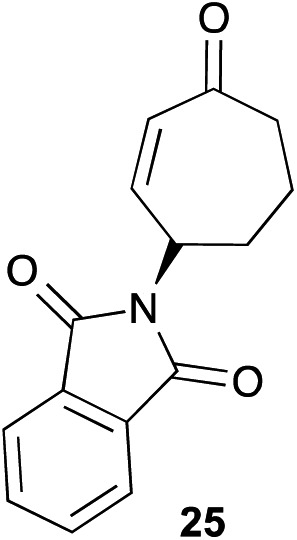	63^*e*^	94

^*a*^Reactions performed with 0.1 or 0.2 mmol **16–18** (99% ee), 2.5 mol% (η^3^-C_3_H_5_)_2_Pd_2_Cl_2_, and 15 mol% PPh_3_ at 0.2 M and 23 °C for 0.5–20 h. For complete details, see the ESI.†

^*b*^Determined by chiral HPLC.

^*c*^Performed at 0 °C with 2.5 mol% Pd_2_dba_3_·CHCl_3_ and 7.5 mol% (*S,S*)-**L1**. 10% of **16** (79% ee) was recovered.

^*d*^Cs_2_CO_3_ added.

^*e*^7.5 mol% dppf used in place of PPh_3_. PMB = *para*-methoxybenzyl, TBS = *tert*-butyldimethylsilyl.

Notably, the products of conjugate addition were not observed in any of these cases. Moreover, despite the sensitivity of **17** toward hydroxide or alkoxide base, the basic nature of the reactions did not hinder us from obtaining good yields of the desired products.

The transformations proceeded with high levels of chirality transfer. This occurred despite the potential for racemization *via* π–σ–π interconversion involving an *O*-bound palladium enolate, a means by which the enantiotopic faces of the π-allylpalladium species might be equilibrated as in the case of butenolide electrophiles.^12^ The substitution appears to proceed primarily through overall double inversion, with the absolute stereochemistry of the starting material being conserved in the product.^13^

**Fig. 6:**
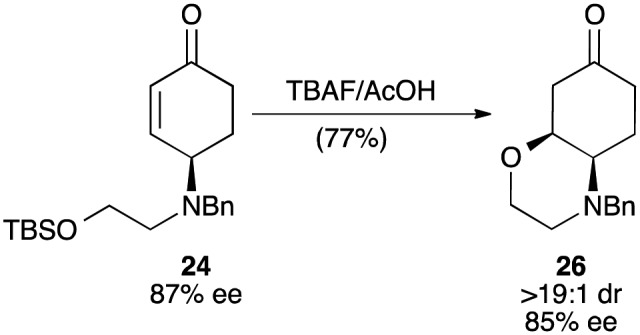
Elaboration of **24** to bicyclic ketone **26**. *Reagents and conditions*: TBAF·3H_2_O, AcOH, THF, 0 → 23 °C, 2 h, 77%.

Further elaboration of these Pd-AA products to more complex cycloalkanone derivatives could be achieved through reactions that engaged the enone moiety with the γ-substituent. For example, exposure of ethanolamine derivative **24** to buffered TBAF effected desilylation and oxa-Michael addition, providing bicyclic morpholine derivative **26** (Fig. 6). The rapid, stereocontrolled synthesis of this compound further highlights the utility of this tandem oxidative desymmetrization/allylic substitution process: the former reaction sets high stereochemical purity at the γ-position that is relayed in the diastereoselective conjugate addition, and the latter enables the introduction of a useful bis-nucleophile unit for a convergent synthesis of **26**.

**Fig. 7:**
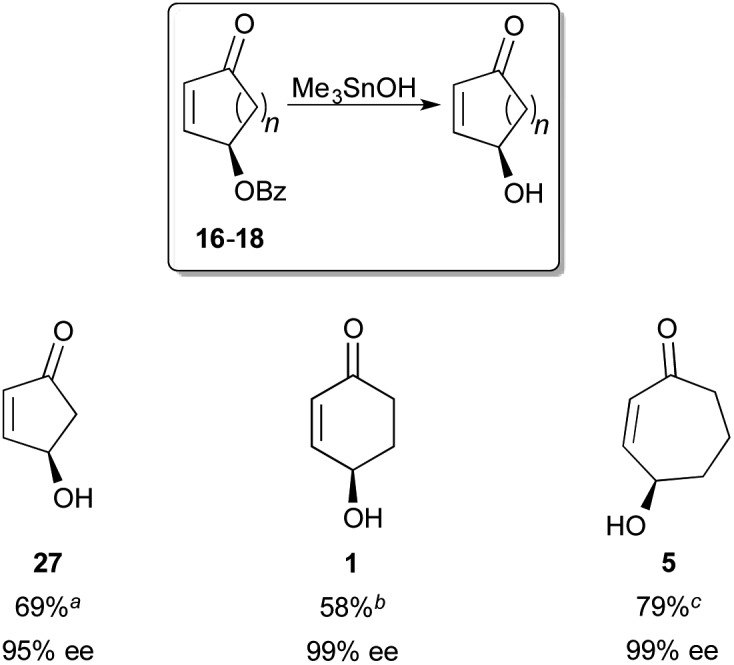
Synthesis of γ-hydroxycycloalkenones. *Reagents and conditions*: 0.2 mmol **16–18** (99% ee), Me_3_SnOH, 1,2-dichloroethane, 80 °C, 14 h. Yields at (*a*) 65%, (*b*) 73%, and (*c*) 66%, conversion (b.r.s.m.).

Compounds **16–18** were also converted to the corresponding enantioenriched γ-hydroxycycloalkenones **1**, **5**, and **27** (Fig. 7). In evaluating methods to hydrolyze these base-sensitive compounds, Me_3_SnOH—which is most typically used for the hydrolysis of methyl esters to their acids^14^—emerged as the optimal reagent for this transformation. To the best of our knowledge, this represents its first use for the saponification of an acyloxy group at a secondary stereocenter, to yield a chiral alcohol. In these events, the γ-hydroxy products were obtained with excellent levels of enantioenrichment, and the sense of absolute stereochemistry was retained in the products.^15^

**Fig. 8:**
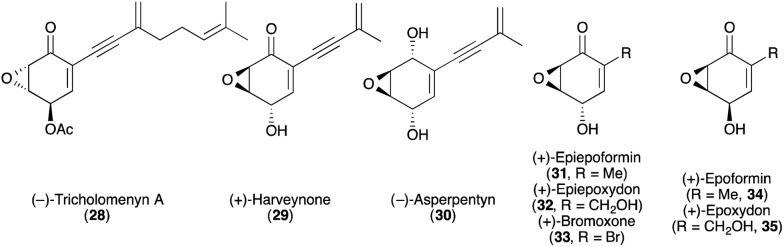
Stereochemically diverse epoxyquinoid natural products.

Emboldened by the chemo-, enantio-, and diastereoselective preparation of the products illustrated in Table 1, Fig. 6 and 7, we next examined the synthesis of more densely functionalized cyclohexenone-derived natural products. We targeted several members of the epoxyquinoid class of natural products, compounds **28–35** (Fig. 8).^16^ These biologically active natural products bear an intriguing stereochemical feature: although structurally similar to each other, certain members exhibit the opposite sense of absolute stereochemistry (*e.g.*, **28** *vs.* **29–33**), while others display different relative stereochemistry between the epoxide and the alcohol (*e.g.*, **31** and **32** *vs.* **34** and **35**). Moreover, for at least one member of this family (harveynone, **29**), both enantiomers are naturally occurring yet are derived from dissimilar sources and exhibit different biological activities.^16*a*^

**Fig. 9:**
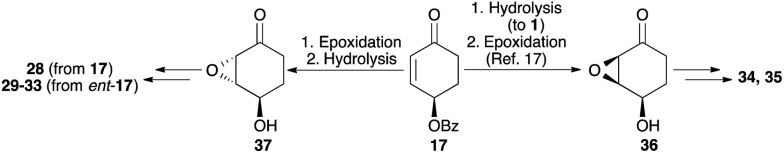
Stereodivergent access to epoxyquinoid precursors.

We proposed a unified strategy toward the structural cores of **28–35** from chiral cyclohexenones **17** and *ent*-**17** (Fig. 9). This approach would exploit the ability of an appropriately γ-substituted cyclohexenone to direct the diastereoselectivity of an intermolecular nucleophilic epoxidation. For instance, γ-hydroxycyclohexenone **1** is known to undergo hydroxyl-directed *syn* epoxidation.^17^ Thus, the preparation of **1** from **17** establishes access to *cis* product **36**, the core structure of **34** and **35**. In contrast, **17** could be expected to undergo *anti*-selective epoxidation. Hydrolysis would then deliver *trans* product **37**, featuring the absolute and relative stereochemistry of **28**. Performing the latter procedure with *ent*-**17** would provide *ent*-**37**, a precursor to **29–33**. Through this strategy, the core structures of all of the stereochemically diverse products **28–35** could be accessed from a common precursor, *meso*-diester **14**.

**Fig. 10:**
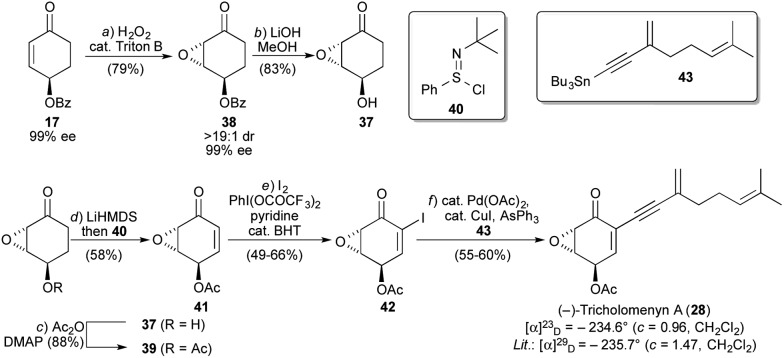
Total synthesis of (–)-tricholomenyn A (**28**). *Reagents and conditions*: (*a*) H_2_O_2_ (30% aq.), 10 mol% Triton B (40% aq.), THF, 0 °C, 45 min, 79%; (*b*) LiOH, MeOH, 0 °C, 1 h, 83%; (*c*) Ac_2_O, DMAP, MeCN, 0 → 23 °C, 10 min, 88%; (*d*) LiHMDS, THF, –78 °C, 30 min, then **40**, –78 °C, 30 min, 58%; (*e*) I_2_, PhI(OCOCF_3_)_2_, pyridine, BHT, CH_2_Cl_2_, 23 °C, 24 h, 49–66%; (*f*) 10 mol% Pd(OAc)_2_, 10 mol% CuI, 20 mol% AsPh_3_, **43**, THF, 0 °C, 1 h 30 min, 55–60%. Triton B = benzyltrimethylammonium hydroxide, DMAP = 4-dimethylaminopyridine, HMDS = hexamethyldisilazane, BHT = butylated hydroxytoluene.

Focusing on the synthesis of (–)-tricholomenyn A (**28**), we investigated the epoxidation of **17** (Fig. 10). Pleasingly, upon exposure to H_2_O_2_ and catalytic benzyltrimethylammonium hydroxide (Triton B),^18^ **17** cleanly underwent epoxidation to give **38** as a single diastereomer (^1^H NMR) and in 99% ee (chiral HPLC). Thus, the oxygenation and the absolute and relative stereochemistry of the *trans* epoxyquinoids were set, with excellent enantio- and diastereoselectivity, in three steps from commercial 1,3-cyclohexadiene.^19^ The fact that the oxidative desymmetrization delivered **17** with a γ-benzoyloxy substituent proved to be a significant synthetic advantage, as the epoxidation of the γ-acetoxy analogue led to poorer diastereoselectivity and to the formation of byproducts. These results are consistent with the observations of Bayón, Figueredo, and co-workers.^20^

Hydrolysis and acetylation proceeded smoothly, affording **39**. Completion of the synthesis then required oxidation of the ketone to an enone, α-iodination, and side chain installation. A number of oxidation conditions were investigated on both **38** and **39** (*e.g.*, selenylation/elimination, Saegusa-Ito oxidation, bromination/elimination, IBX oxidation of a silyl enol ether,^21^ and Pd(TFA)_2_-catalyzed aerobic oxidation^22^), all of which led to no or trace product and/or decomposition.^23^ Procedures involving the use of a lithium enolate at temperatures above –78 °C proved especially problematic, leading to extensive decomposition. As oxidations of this sort have been performed on γ-silyloxy, α,β-epoxyketones,^18^ we suspected that the incompatibility was due to the ester moiety, the electrophilic nature of which promoted side reactions. However, rather than lengthening the synthesis with a protection/deprotection sequence involving alcohol **37**, we investigated procedures in which the ketone enolate of **39** could be both formed and reacted at low temperature. We turned to the method of Mukaiyama,^24^ which involved sulfenylation of the lithium enolate of **39** with **40** and subsequent elimination, both of which proceeded efficiently at –78 °C to deliver **41**.

The conversion of **41** to iodoenone **42** initially proved challenging, as typical conditions (I_2_ in CH_2_Cl_2_/pyridine^25^) led to decomposition. This may have been the result of interactions between the acetoxy group of **41** and these co-solvent amounts of pyridine. Success was realized by applying the conditions of Benhida^26*a*^ (as applied by Hayashi^26*b*^), which employ only stoichiometric pyridine and a more activated iodinating reagent.

From **42**, cross coupling with known stannane **43** ^16*e*^ afforded (–)-tricholomenyn A (**28**), the analytical data for which matched literature data. The observed optical rotation ([*α*]23D = – 234.6° (*c* = 0.96, CH_2_Cl_2_)) was in line with that reported for material obtained using enzymatic resolution as the source of chirality ([*α*]29D = – 235.7° (*c* = 1.47, CH_2_Cl_2_)).^27^

**Fig. 11:**
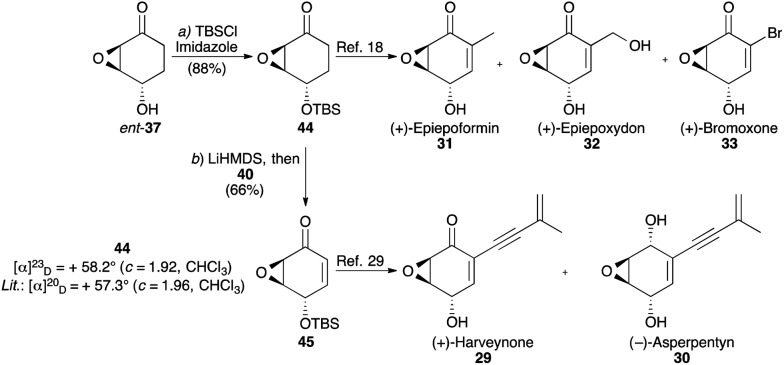
Syntheses of epoxyquinoids **29–33**. *Reagents and conditions*: (*a*) TBSCl, imidazole, CH_2_Cl_2_, 23 °C, 16 h, 88%; (*b*) LiHMDS, THF, –78 °C, 30 min, then **40**, –78 °C, 1 h, 66%.

Synthetic access to natural products **29–33** was then established through the synthesis of *ent*-**37** from *ent*-**17** (prepared from **14** in 98% ee using (*S*,*S*)-**L1**). Silylation afforded **44**, from which syntheses of **31–33** are known (Fig. 11). The analytical data for **44**, including optical rotation, was in line with data reported for material prepared through an enzymatic approach.^18^

The asymmetric synthesis of **44** has itself been the subject of past studies, for both natural product synthesis and pharmaceutical research.^18,28^ Prior syntheses have employed enzymatic reduction,^18^ enzymatic resolution,^28*a*^ chiral pool material,^28*b*^ or asymmetric deprotonation,^28*c*^ strategies that either required eight or more steps or, in the latter case, delivered more modest (77–85%) ee. The five-step procedure presented herein, which provides 98–99% ee, complements these approaches.

The oxidation of ketone **44** to enone **45** has previously been performed *via* silyl enol ether formation followed by either selenylation/elimination^18^ or stoichiometric Saegusa-Ito oxidation.^28*a*^ Given our successful oxidation of ketone **39** *via* the Mukaiyama protocol, we considered performing this reaction on **44** to deliver **45** directly in one step. Indeed, this protocol proved very effective, providing the enone in good yield. From **45**, syntheses of **29** and **30** are known.^29^

## Conclusions

We report a strategy for the synthesis of a diverse set of chiral cycloalkenone derivatives *via* asymmetric palladium catalysis. Using a newly optimized reaction procedure, the oxidative desymmetrization of *meso*-dibenzoates **13–15** affords cycloalkenones **16–18** in good yields and with excellent enantioselectivity. These products serve as platforms from which a variety of substituted cycloalkenones can be prepared through palladium-catalyzed allylic alkylation, including established building blocks such as *ent*-**2** and **4**. The products are obtained in good yields and with high levels of enantioenrichment. This Pd-AA approach also enables the convergent introduction of useful functionality, allowing for the synthesis of more elaborate cycloalkanone derivatives. The hydrolysis of **16–18** to enantioenriched γ-hydroxycycloalkenone building blocks is also demonstrated.

Cyclohexenones **17** and *ent*-**17** provide entry points to epoxyquinoid natural products **28–35** through stereoselective transformations and through reactions that elaborate the entire cyclohexenone structure. This approach to these stereochemically diverse products proceeds with excellent levels of enantio- and diastereoselectivity, and it establishes a concise, unified strategy starting from a single *meso* precursor (**14**).
