# Supplementary material for: Asymmetric synthesis of chiral cycloalkenone derivatives via palladium catalysis
Source: Chem Sci. 2014 Jan 27;5(4):1354–60. doi: 10.1039/c3sc53250j (PMC3992253; doi:10.1039/c3sc53250j)
Supplement: Supplementary file 1 [file SC-005-C3SC53250J-s001.pdf]

Electronic Supplementary Material (ESI) for Chemical Science
This journal is © The Royal Society of Chemistry 2014

Asymmetric Synthesis of Chiral Cycloalkenone Derivatives *via* Palladium Catalysis

Barry M. Trost,* James T. Masters, Jean-Philip Lumb, and Dahlia Fateen

Department of Chemistry, Stanford University, Stanford, California 94305-4401

Supporting Information

Table of Contents

1. Experimental Procedures

a)	General Considerations	2 – 3
b)	Synthesis of *meso* Dibenzoate **14**	4
c)	Synthesis of Oxidative Desymmetrization Products **16-18**	5 – 7
d)	Synthesis of Allylic Alkylation Products *ent*-**2**, **4**, and **19-25**	8 – 17
e)	Synthesis of Compound **26**	17 – 18
f)	Synthesis of γ-Hydroxycycloalkenones **1**, **5**, and **27**	18 – 21
g)	Total Synthesis of (–)-Tricholomenyn A (**28**)	21 – 29
h)	Synthesis of Epoxyquinoid Precursors **44** and **45**	29 – 30

2. Analytical Data

a)	^{1}H and ^{13}C NMR Spectra	31 – 72
b)	Chiral HPLC Chromatograms	73 – 103

Electronic Supplementary Material (ESI) for Chemical Science
This journal is © The Royal Society of Chemistry 2014

1. Experimental Procedures

a) General Considerations. Unless otherwise indicated, all reactions were performed in oven- or flame-dried glassware with magnetic stirring under a nitrogen or argon atmosphere. Air- and moisture-sensitive liquids and solutions were transferred *via* oven-dried, stainless steel syringe or cannula and were introduced into the reaction vessel through rubber septa. Reactions performed below room temperature (23 °C) were cooled with ice/water baths (0 °C) or dry ice/isopropanol baths (− 78 °C). Anhydrous PhMe, PhH, CH_2Cl_2 and THF were obtained from a Seca solvent purification system by Glass Contour. Hexamethyldisilazane (HMDS) and 1,2-dichloroethane (DCE) were distilled from CaH_2 under nitrogen. For use in Pd-AA reactions—except the oxidations of *meso* diesters, as noted below—anhydrous and deoxygenated THF was obtained from a Na/benzophenone ketyl still under argon. Unless otherwise indicated, yields are of isolated products. pH 7 buffer was prepared by dissolving K_2HPO_4 (99 g) and KH_2PO_4 (77 g) in DI water (1 L). *Meso* dibenzoates **13** and **15** were prepared by benzoylation of the corresponding diols.[1] $Pd_2dba_3{\cdot}CHCl_3$,[2] ligand **L1**,[3] and IBX (2-iodoxybenzoic acid)[4] were prepared by literature procedures. $(\eta^3\text{-}C_3H_5)_2Pd_2Cl_2$ was prepared by the literature procedure[5] and then crystallized from CH_2Cl_2 / hexanes.

Analytical and preparative thin-layer chromatography was performed using pre-coated 250 μm layer thickness silica gel 60 F_{254} plates (EMD Chemicals Inc.). Visualization was performed by ultraviolet light fluorescence quenching and/or by staining with aqueous potassium permanganate, aqueous ceric ammonium molybdate, or ethanolic *para*-anisaldehyde solutions followed by heating. Flash column chromatography was performed using 40-63 μm silica gel (Silicycle silica gel) using compressed air. The eluent employed for flash chromatography is reported using volume/volume ratios. Proton nuclear magnetic resonance (^{1}H NMR) spectra were acquired using a Varian Inova 600 MHz, Varian Inova 500 MHz, Varian Inova 300 MHz, or Varian Mercury 400 MHz spectrometer. Chemical shifts (δ) are reported in parts per million

---

[1] See a) I. Erden, C. Gärtner, M. Saeed Azimi, *Org. Lett.*, **2009**, *11*, 3986; b) J. Lou, M. Hashimoto, N. Verova, K. Nakanishi, *Org. Lett.*, **1999**, *1*, 51; c) B. M. Trost, J. Richardson, K. Yong, *J. Am. Chem. Soc.*, **2006**, *128*, 2540.
[2] T. Ukai, H. Kawazura, Y. Ishii, J. J. Bonnet, J. A. Ibers, *J. Organomet. Chem.* **1974**, *65*, 253.
[3] B. M. Trost, D. L. van Vranken, C. Bingel, *J. Am. Chem. Soc.* **1992**, *114*, 9327.
[4] M. Frigerio, M. Santagostino, S. Sputore, *J. Org. Chem.*, **1999**, *64*, 4537.
[5] N. Marion, O. Navarro, J. Mei, E. D. Stevens, N. M. Scott, S. P. Nolan, *J. Am. Chem. Soc.*, **2006**, *128*, 4101.

Electronic Supplementary Material (ESI) for Chemical Science
This journal is © The Royal Society of Chemistry 2014

(ppm) and are calibrated to the residual solvent peak ($CHCl_3$, 7.26 ppm). Coupling constants (*J*) are reported in Hz. Multiplicities are reported using the following abbreviations: app = apparent, s = singlet; d = doublet; t = triplet; q = quartet; m = multiplet (range of multiplet is given). Carbon nuclear magnetic resonance (^{13}C NMR) spectra were recorded using a Varian Inova 150 MHz, Varian Inova 125 MHz, Varian Inova 75 MHz, or a Varian Mercury 100 MHz spectrometer. Chemical shifts (δ) are reported in parts per million (ppm) and are calibrated to the residual solvent peak ($CHCl_3$, 77.16 ppm).

Infrared spectroscopic data were recorded on a Thermo Scientific Nicolet IR100 FT-IR spectrometer, using thin films of the sample on NaCl plates. The absorbance frequencies are recorded in wavenumbers (cm^{-1}). Chiral HPLC analysis was performed using an Agilent Technologies 1200 Series HPLC equipped with a Daicel CHIRALPAK® chiral stationary phase column (IA [amylose tris(3,5-dimethylphenylcarbamate) immobilized on silica support], IB [cellulose tris(3,5-dimethylphenylcarbamate) immobilized on silica support], or IC [cellulose tris(3,5-dichlorophenylcarbamate) immobilized on silica support]) or using a SpectraSYSTEM™ P1000 pump equipped with a SpectraSERIES™ UV100 detector and a Daicel chiral stationary phase column (CHIRALCEL® OD-H [cellulose tris(3,5-dimethylphenylcarbamate) immobilized on silica support], CHIRALCEL® OJ-H [cellulose tris (4-methylbenzoate) immobilized on silica support]), or CHIRALPAK® AD-H ([amylose tris(3,5-dimethylphenylcarbamate) immobilized on silica support]). Retention times of enantioenriched products were determined either from admixtures of the two enantiomers, each independently prepared from the *meso* dibenzoate using (*R*,*R*) or (*S*,*S*)-**L1**, or from racemic materials prepared from racemic γ-hydroxycycloalkenones.

Optical rotations were measured using a JASCO P2000 polarimeter using 5 cm glass cells with a sodium 589 nm filter and are reported as $[\alpha]_D^T$, concentration (g/100 mL), and solvent. Melting points were determined on a Thomas Hoover Capillary Melting Point Apparatus and are uncorrected. High-resolution mass spectra were acquired by the Vincent Coates Foundation Mass Spectrometry Laboratory, Stanford University (http://massspec.stanford.edu).

b) Synthesis of *meso* Dibenzoate 14.

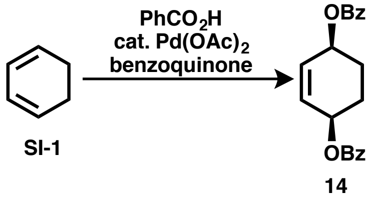

14: Based on the procedure of Bäckvall and co-workers.[6] A 500 mL round-bottom flask was charged with $PhCO_2H$ (37.0 g, 304 mmol, 7.6 equivalents), benzoquinone (8.85 g, 81.9 mmol, 2.05 equivalents), $Pd(OAc)_2$ (358 mg, 1.59 mmol, 0.04 equivalents), and acetone (250 mL). The resulting dark, heterogeneous reaction mixture was stirred for 15 minutes, and then 1,3-cyclohexadiene (**SI-1**, 3.8 mL, 3.2 g, 39.9 mmol, 1 equivalent) was added *via* syringe pump over 4 hours. The reaction mixture was stirred for 14 additional hours, concentrated to a black residue, dissolved in Et_2O (750 mL), and washed with 2 N NaOH (2 x 150 mL). As noted by Bäckvall and co-workers, the *cautious* addition of a small amount ($\leq$ 500 mg) of $NaBH_4$ helped improve phase separation, and this was necessary on some occasions. The pooled aqueous phases were extracted with Et_2O (200 mL), and to the pooled organics was added *ca.* 10 g SiO_2. The pooled organics were dried over $MgSO_4$, filtered, and concentrated to an oil, which was passed through a column of SiO_2 eluting with 4:1 hexanes:Et_2O. Concentration to a white solid and recrystallization from heptane (100 mL) afforded 7.8 g of **14** as a white solid. The filtrate was concentrated and recrystallized from heptane (20 mL) to afford a second crop of **14** (0.48 g, 8.28 g total, 25.69 mmol, 64%).

^{1}H NMR (500MHz, $CDCl_3$): 8.09-8.08 (m, 4H), 7.59-7.56 (m, 2H), 7.47-7.44 (m, 4H), 6.09 (d, J = 1.3 Hz, 2H), 5.45 (d, J =1.5 Hz, 2H), 2.11-2.09 (m, 4H)

Analytical data matched literature data.

[6] J. E. Bäckvall, K. L. Granberg, R. B. Hopkins, *Acta Chem. Scand.* **1990**, *44*, 492.

c) Synthesis of Oxidative Desymmetrization Products 16-18.

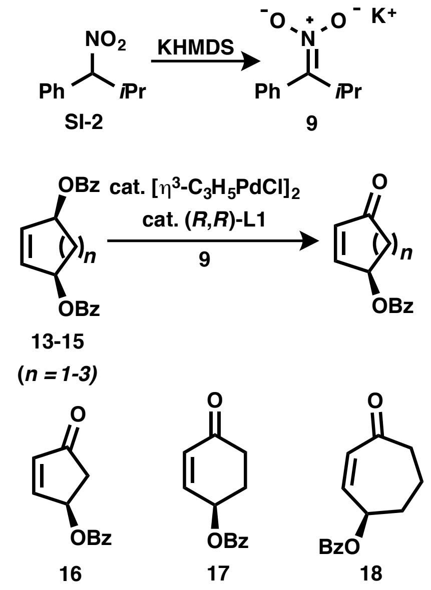

Representative procedure for oxidative desymmetrization: **Synthesis of 17.**

A flame-dried 250 mL round-bottom flask equipped with a stir bar was charged with THF (*ca*. 200 mL, Seca solvent system), which was sparged with a vigorous N_2 flow (Schlenk line) for 30 min. A separate 250 mL round-bottom flask was charged with a large stir bar, sealed with a septum, and flame-dried under vacuum. The mass of this flask/stir bar/septum system was recorded. The septum was removed, the flask was tared, and KH (*ca.* 30% dispersion in mineral oil, 2.22 g) was added. The flask was sealed with the septum and then evacuated and backfilled with N_2. The KH dispersion in oil was washed with THF (3 x 10 mL), using a cannula to remove the washings. These washings, which contain trace amounts of KH, were cautiously quenched at 0 °C with, sequentially, isopropanol, methanol, and water. The flask was quickly flame-dried under vacuum to afford KH as a free-flowing gray powder (0.74 g, determined by difference in mass, 18.45 mmol, 2.05 equivalents). The flask was backfilled with N_2 and placed in a 0 °C bath, and then THF (37 mL) was added, followed by HMDS dropwise (4.25 mL, 3.27 g, 20.3 mmol, 2.26 equivalents, 1.1 equivalents relative to KH). The mixture was stirred at 0 °C for 15 min, and then the cooling bath was removed and the mixture was stirred 30 min at room temperature, to afford a slightly cloudy solution of KHMDS in THF. The mixture was cooled to 0 °C, and a solution of (2-methyl-1-nitropropyl)benzene[1c] (**SI-2**, 4.09 g, 18.9 mmol, 2.1 equivalents) in THF

(38 mL total, including rinses) was added *via* cannula. The mixture turned yellow during this time, and, after 1-2 min, a cream-colored heterogeneous mixture of nitronate **9** formed. The cooling bath was then removed, and the mixture was stirred for 30 min. During this time, the catalyst solution was prepared by combining $(\eta^3\text{-}C_3H_5)_2Pd_2Cl_2$ (49.4 mg, 0.135 mmol, 0.015 equivalent, finely ground with a spatula) and (*R*,*R*)-**L1** (280 mg, 0.405 mmol, 0.045 equivalent) in a flame-dried 3-dram vial equipped with a stir bar, which was evacuated and backfilled with N_2 and then treated with THF (5 mL). The bright yellow, homogeneous catalyst solution was stirred for vigorously at least 10 min. A flame-dried 50 mL round-bottom flask equipped with a stir bar was charged with **14** (2.90 g, 9.0 mmol, 1.0 equivalent), sealed with a septum, evacuated, backfilled with N_2, treated with THF (24 mL), and stirred to effect complete dissolution (< 5 min). The flask containing **9** was cooled to 0 °C, and the solution of **14** was transferred to the former *via* cannula, using additional THF (6 mL) to rinse the flask and cannula. The catalyst solution was then immediately transferred *via* cannula. Care was taken to maintain vigorous stirring throughout the additions. The cooling bath was removed after the additions, and the resulting yellow-orange, heterogeneous mixture was stirred vigorously for 30 min, at which point TLC analysis (3:1 hexanes:Et_2O) indicated complete consumption of **14**. The reaction mixture was poured into a separatory funnel containing Et_2O (200 mL) and pH 7 phosphate buffer (150 mL), which was shaken thoroughly for 1-2 min. The phases were separated, and the aqueous phase was extracted with Et_2O (100 mL). The pooled organics were dried over $MgSO_4$, filtered, and concentrated. The residue was purified by column chromatography (6:1→4:1→2:1 hexanes:Et_2O, crude material loaded using 6:1 hexanes:Et_2O and some CH_2Cl_2) to afford **17** (1.30 g, 6.01 mmol, 67%, 99% ee) as a light yellow, viscous oil that solidified to a white solid on standing in a refrigerator.

^{1}H NMR (500 MHz; $CDCl_3$): δ 8.07-8.05 (m, 2H), 7.60 (tt, *J* = 7.5, 1.4 Hz, 1H), 7.49-7.45 (m, 2H), 6.98 (ddd, *J* = 10.2, 2.7, 1.3 Hz, 1H), 6.13-6.11 (m, 1H), 5.85-5.81 (m, 1H), 2.73-2.67 (m, 1H), 2.57-2.52 (m, 1H), 2.51-2.46 (m, 1H), 2.30-2.22 (m, 1H)

Chiral HPLC: OJ-H, 90:10 heptane:isopropanol, 0.50 mL/min, 254 nm, 29.48 min (*R* enantiomer), 34.32 min (*S* enantiomer)

$[\alpha]_D^{23}$ = + 167° (c = 0.85, $CHCl_3$), Lit.: $[\alpha]_D$ = + 201 (c = 0.85, $CHCl_3$)[7]

---

[7] L. Yu, R. Zhang, Z. Wang, *J. Chem. Soc., Perkin Trans. 1*, **2001**, 2958.

For (*S*)-**17**, prepared as described above with (*S*,*S*)-**L1** in 98% ee, $[\alpha]_D^{23}$ = − 178° (c = 0.85, $CHCl_3$), Lit.: $[\alpha]_D$ = −197 (c = 1.9, CH_2Cl_2)[8]

M.P. = 32-33 °C

Analytical data matched previously reported data.[1c]

Cycloalkenones **16** and **18** were prepared following the above procedure. Analytical data matched previously reported data and are partially reproduced below. For cyclopentenone **16**, the reaction was quenched 10 min after the addition of the catalyst solution (**13** consumed by TLC analysis). Compound **16** was obtained in 78% yield (0.859 mmol) and 99% ee (1.1 mmol reaction scale). For cycloheptenone **18**, the reaction was quenched 3 h after the addition of the catalyst solution, at which point the reaction had reached 68% conversion based on 32% recovered **15** (0.395 mmol). Compound **18** was obtained in 50% isolated yield (0.621 mmol, 73% yield based on 68% conversion) and 99% ee (1.25 mmol reaction scale).

16:

^{1}H NMR (400 MHz; $CDCl_3$): δ 8.05-8.03 (m, 2H), 7.71 (dd, *J* = 5.7, 2.4 Hz, 1H), 7.62-7.58 (m, 1H), 7.49-7.44 (m, 2H), 6.41 (dd, *J* = 5.7, 1.3 Hz, 1H), 6.12 (dtd, *J* = 6.4, 2.3, 1.3 Hz, 1H), 2.96 (dd, *J* = 18.8, 6.4 Hz, 1H), 2.50 (dd, *J* = 18.8, 2.2 Hz, 1H)

Chiral HPLC: OJ-H, 90:10 heptane:isopropanol, 0.50 mL/min, 254 nm, 33.74 min (*R* enantiomer), 37.56 min (*S* enantiomer)

$[\alpha]_D^{25}$ = + 184° (c = 0.38, $CHCl_3$)

18:

^{1}H NMR (400 MHz; $CDCl_3$): δ 8.08-8.05 (m, 2H), 7.61-7.57 (m, 1H), 7.49-7.45 (m, 2H), 6.58 (ddd, *J* = 12.5, 3.3, 1.2 Hz, 1H), 6.09 (ddt, *J* = 12.5, 2.2, 0.6 Hz, 1H), 5.85 (dddd, *J* = 9.5, 4.6, 3.3, 2.2 Hz, 1H), 2.71-2.68 (m, 2H), 2.38-2.29 (m, 1H), 2.09-1.92 (m, 3H).

Chiral HPLC: OJ-H, 90:10 heptane:isopropanol, 0.50 mL/min, 254 nm, 32.14 min (*S* enantiomer), 35.26 min (*R* enantiomer)

$[\alpha]_D^{25}$ = + 131° (c = 0.41, $CHCl_3$)

---

[8] R. J. Kazlauskas, A. N. E. Weissfloch, A. T. Rappaport, L. A. Cuccia, *J. Org. Chem.* **1991**, *56*, 2656.

This journal is © The Royal Society of Chemistry 2014

d) Synthesis of Allylic Alkylation Products *ent*-2, 4, and 19-25.

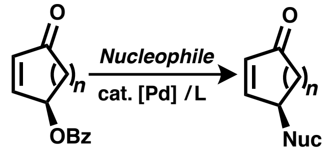

16-18

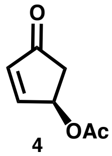

4

4: A round-bottom Biotage® microwave vial (2.0 mL – 5.0 mL size) equipped with a large stir bar was charged with Pd_2dba_3·$CHCl_3$ (2.6 mg, 0.025 mmol, 0.025 equivalents), (*S*,*S*)-**L1** (5.2 mg, 0.0075 mmol, 0.075 equivalents), and powdered NaOAc (24.6 mg, 0.3 mmol, 3 equivalents). The vial was sealed with a septum and evacuated and backfilled with N_2. Distilled THF (0.50 mL) was added, and the mixture was stirred for 10 minutes at room temperature to afford a heterogeneous, orange mixture. The vial was cooled to 0 °C, and solid **16** (20.2 mg, 0.0999 mmol, 1 equivalent) was added under N_2 flow. The reaction mixture was stirred for 3 h at 0 °C, at which point it was diluted with EtOAc (5 mL) and poured into saturated aqueous $NaHCO_3$ (5 mL). The phases were separated, and the aqueous phase was extracted with EtOAc (4 x 5 mL). The pooled organics were washed with saturated aqueous $NaHCO_3$ (5 mL), dried over $MgSO_4$, filtered, and concentrated. Purification of the residue by column chromatography (4:1→2:1 hexanes:EtOAc) afforded **4** (8.9 mg, 0.0635 mmol, 64%, 93% ee) as a clear, light yellow oil as well as recovered **16** (2.1 mg, 0.010 mmol, 10%, 79% ee).

^{1}H NMR (500 MHz; $CDCl_3$): 7.57 (dd, *J* = 5.7, 2.4 Hz, 1H), 6.34 (dd, *J* = 5.7, 1.3 Hz, 1H), 5.87-5.84 (m, 1H), 2.83 (dd, *J* = 18.7, 6.4 Hz, 1H), 2.33 (dd, *J* = 18.7, 2.2 Hz, 1H), 2.10 (s, 3H)

^{13}C NMR (125 MHz; $CDCl_3$): δ 205.1, 170.6, 159.1, 137.2, 72.1, 41.2, 21.0

Chiral HPLC: AD-H, 95:5 heptane:isopropanol, 0.80 mL/min, 220 nm, 11.15 min (*R* enantiomer), 12.17 min (*S* enantiomer)

$[\alpha]_D^{24}$ = + 53.8° (c = 0.81, $CHCl_3$), Lit: + 95° (c = 0.1, $CHCl_3$)[9]

Analytical data matched literature data.[10]

[9] M. Le Liepvre, J. Ollivier, D. J. Atiken, *Eur. J. Org. Chem.,* **2009**, 5953.
[10] K. Ulbrin, P. Kreitmeier, T. Vilaivan, O. Reiser, *J. Org. Chem.*, **2013**, *78*, 4202.

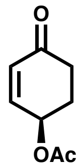

19

19: A round-bottom Biotage® microwave vial (2.0 mL – 5.0 mL size) equipped with a large stir bar was charged with **17** (40.7 mg, 0.188 mmol, 1 equivalent), (η^3-$C_3H_5)_2Pd_2Cl_2$ (1.7 mg, 0.005 mmol, 0.025 equivalents), PPh_3 (7.8 mg, 0.03 mmol, 0.15 equivalents), and powdered NaOAc (82 mg, 1 mmol, 5 equivalents). The vial was sealed with a septum-lined cap and evacuated and backfilled with N_2. Distilled THF (1.0 mL) was added, the pierced septum cap was sealed thoroughly with electrical tape and Parafilm®, and the resulting heterogeneous, yellow mixture was stirred vigorously for 3 h. The reaction mixture was then diluted with EtOAc (5 mL) and poured into saturated aqueous $NaHCO_3$ (5 mL). The phases were separated, and the aqueous phase was extracted with EtOAc (4 x 5 mL). The pooled organics were washed with saturated aqueous $NaHCO_3$ (5 mL), dried over $MgSO_4$, filtered, and concentrated. Purification of the residue by column chromatography (4:1→2:1 hexanes:EtOAc) afforded **19** (21.7 mg, 0.141 mmol, 75%, 96% ee) as a clear, light yellow oil.

^{1}H NMR (400 MHz; $CDCl_3$): δ 6.84 (ddd, *J* = 10.3, 2.8, 1.5 Hz, 1H), 6.05 (ddd, *J* = 10.3, 1.9, 0.9 Hz, 1H), 5.58-5.53 (m, 1H), 2.65-2.58 (m, 1H), 2.48-2.40 (m, 1H), 2.37-2.32 (m, 1H), 2.11 (s, 3H), 2.13-2.04 (m, 1H)

^{13}C NMR (100 MHz; $CDCl_3$): δ 198.1, 170.5, 147.8, 131.0, 67.9, 35.1, 28.8, 21.2

IR: 2921, 1740, 1688, 1372, 1237, 1038, 895 cm^{-1}

Chiral HPLC: OJ-H, 95:5 heptane:isopropanol, 0.50 mL/min, 220 nm, 40.72 min (*R* enantiomer), 43.29 min (*S* enantiomer)

$[\alpha]_D^{23}$ = + 119.6° (c = 1.0, $CHCl_3$), Lit: + 113° (c = 1.48, $CHCl_3$)[11]

Analytical data matched literature data.[11]

[11] H. Suzuki, N. Yamazaki, C. Kibayashi, *J. Org. Chem.*, **2001**, *66*, 1494.

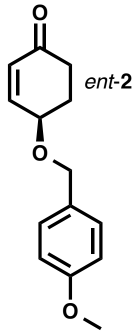
ent-**2**

ent-**2**: A round-bottom Biotage® microwave vial (2.0 mL – 5.0 mL size) equipped with a stir bar was charged with **17** (21.8 mg, 0.101 mmol, 1 equivalent), (η^3-C_3H_5)$_2$$Pd_2Cl_2$ (0.9 mg, 0.0025 mmol, 0.025 equivalents), and PPh_3 (3.9 mg, 0.015 mmol, 0.15 equivalents). The vial was sealed with a septum-lined cap and evacuated and backfilled with N_2. Degassed (N_2 sparge) PhMe (0.50 mL) was added, followed immediately by 4-methoxybenzyl alcohol (62 μL, 0.50 mmol, 5 equivalents). The pierced septum cap was sealed thoroughly with electrical tape and Parafilm®, and the resulting homogeneous, yellow mixture was stirred for 10 h, during which time its appearance changed to homogeneous and yellow-green. The reaction mixture was then diluted with Et_2O (5 mL) and poured into saturated aqueous $NaHCO_3$ (5 mL). The phases were separated, and the aqueous phase was extracted with Et_2O (3 x 5 mL). The pooled organics were dried over $MgSO_4$, filtered, and concentrated. Purification of the residue by column chromatography (2:1 hexanes:EtOAc) afforded *ent*-**2** (14.5 mg, 0.0624 mmol, 62%, 91% ee) as a light yellow oil.

^{1}H NMR (500 MHz; $CDCl_3$): δ 7.31-7.28 (m, 2H), 6.97 (ddd, J = 10.3, 2.4, 1.4 Hz, 1H), 6.92-6.89 (m, 2H), 5.99 (ddd, J = 10.3, 1.9, 1.0 Hz, 1H), 4.58 (q, J = 12.3 Hz, 2H), 4.25 (ddt, J = 9.3, 4.7, 2.3 Hz, 1H), 3.81 (s, 3H), 2.63-2.58 (m, 1H), 2.37-2.30 (m, 2H), 2.08-2.00 (m, 1H).

^{13}C NMR (100 MHz; $CDCl_3$): δ 199.0, 159.6, 150.9, 129.9, 129.8, 129.5, 114.1, 72.3, 70.8, 55.4, 35.5, 29.3

Chiral HPLC: IC, 90:10 heptane:isopropanol, 0.80 mL/min, 220 nm, 33.96 min (*S* enantiomer), 35.95 min (*R* enantiomer)

$[\alpha]_D^{23}$ = + 69.4° (c = 1.0, $CHCl_3$), Lit (**2**): – 89.2° (c = 1.01, $CHCl_3$)[12a]

Analytical data matched literature data.[12]

---

[12] a) J. E. Audia, L. Boisvert, A. D. Patten, A. Villalobos, S. J. Danishefsky, *J. Org. Chem.*, **1989**, *54*, 3738; b) C. Spino, B. Hill, P. Dubé, S. Gingras, *Can. J. Chem.*, **2003**, *81*, 81.

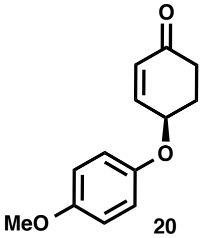

20

20: A round-bottom Biotage® microwave vial (2.0 mL – 5.0 mL size) equipped with a stir bar was charged with **17** (22.3 mg, 0.103 mmol, 1 equivalent), (η^3-$C_3H_5)_2Pd_2Cl_2$ (0.9 mg, 0.0025 mmol, 0.025 equivalents), PPh_3 (3.9 mg, 0.015 mmol, 0.15 equivalents), 4-methoxyphenol (24.8 mg, 0.20 mmol, 2 equivalents), and Cs_2CO_3 (65.2 mg, 0.20 mmol, 2 equivalents). The vial was sealed with a septum-lined cap and evacuated and backfilled with N_2. Degassed (N_2 sparge) CH_2Cl_2 (0.50 mL) was added, the pierced septum cap was sealed thoroughly with electrical tape and Parafilm®, and the resulting heterogeneous, yellow mixture was stirred vigorously for 30 min. The reaction mixture was then diluted with Et_2O (5 mL) and poured into saturated aqueous $NaHCO_3$ (5 mL). The phases were separated, and the aqueous phase was extracted with Et_2O (3 x 5 mL). The pooled organics were dried over $MgSO_4$, filtered, and concentrated. Purification of the residue by column chromatography (4:1→2:1→1:1 hexanes:Et_2O) afforded **20** (17.9 mg, 0.0820 mmol, 80%, 98% ee) as a yellow solid.

R_f = 0.26 (1:1 hexanes:Et_2O)

^{1}H NMR (400 MHz; $CDCl_3$): δ 7.03 (ddd, *J* = 10.3, 2.4, 1.5 Hz, 1H), 6.92-6.83 (m, 4H), 6.06 (ddd, *J* = 10.3, 1.9, 1.0 Hz, 1H), 4.96 (ddt, *J* = 9.3, 4.7, 2.3 Hz, 1H), 3.78 (s, 3H), 2.70-2.62 (m, 1H), 2.47-2.38 (m, 2H), 2.27-2.14 (m, 1H)

^{13}C NMR (100 MHz; $CDCl_3$): δ 198.5, 154.8, 151.0, 149.2, 130.4, 117.7, 115.0, 72.6, 55.8, 35.3, 29.4

IR: 3001, 2915, 2796, 1661, 1486, 1443, 1364, 1271, 1208, 1207, 1024, 930, 861, 816, 764 cm^{-1}

Chiral HPLC: IC, 90:10 heptane:isopropanol, 0.80 mL/min, 220 nm, 28.52 min (*S* enantiomer), 32.10 min (*R* enantiomer)

$[\alpha]_D^{24}$ = + 100.5° (c = 1.0, $CHCl_3$)

M.P. = 48-50 °C

HRMS (ESI): Calculated for $C_{13}H_{15}O_3$ $(M+H)^+$: 219.1016, found 291.1015

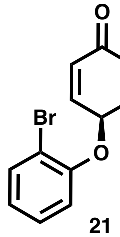

21

21: A round-bottom Biotage® microwave vial (2.0 mL – 5.0 mL size) equipped with a stir bar was charged with **17** (42.0 mg, 0.194 mmol, 1 equivalent), $(\eta^3\text{-}C_3H_5)_2Pd_2Cl_2$ (1.8 mg, 0.005 mmol, 0.025 equivalents), PPh_3 (7.9 mg, 0.03 mmol, 0.15 equivalents), and Cs_2CO_3 (130.3 mg, 0.40 mmol, 2 equivalents). The vial was sealed with a septum-lined cap and evacuated and backfilled with N_2. Degassed (N_2 sparge) CH_2Cl_2 (1.0 mL) was added, followed by 2-bromophenol (47 μL, 0.40 mmol, 2 equivalents). The pierced septum cap was sealed thoroughly with electrical tape and Parafilm®, and the resulting heterogeneous, yellow mixture was stirred vigorously for 30 min. The reaction mixture was then diluted with Et_2O (5 mL) and poured into saturated aqueous $NaHCO_3$ (5 mL). The phases were separated, and the aqueous phase was extracted with Et_2O (3 x 5 mL). The pooled organics were dried over $MgSO_4$, filtered, and concentrated. Purification of the residue by column chromatography (4:1→2:1 hexanes:EtOAc) afforded **21** (34.3 mg, 0.128 mmol, 66%, 92% ee) as a viscous, yellow oil.

R*f* = 0.37 (2:1 hexanes:EtOAc)

^{1}H NMR (400 MHz; $CDCl_3$): δ 7.58 (dd, *J* = 7.9, 1.6 Hz, 1H), 7.29 (ddd, *J* = 8.2, 7.4, 1.6 Hz, 1H), 7.07 (ddd, *J* = 10.3, 2.6, 1.4 Hz, 1H), 7.00 (dd, *J* = 8.3, 1.3 Hz, 1H), 6.92 (ddd, *J* = 7.9, 7.4, 1.4 Hz, 1H), 6.10 (ddd, *J* = 10.3, 1.8, 0.9 Hz, 1H), 5.10-5.05 (m, 1H), 2.78-2.71 (m, 1H), 2.49-2.40 (m, 2H), 2.36-2.27 (m, 1H).

^{13}C NMR (100 MHz; $CDCl_3$): δ 198.2, 153.9, 148.1, 134.1, 130.8, 128.7, 123.6, 116.6, 114.3, 73.4, 35.2, 29.3

IR: 3301, 3021, 2916, 1662, 1562, 1454, 1422, 1363, 1258, 1225, 1188, 1037, 1017, 931, 861, 738 cm^{-1}

Chiral HPLC: IC, 97:3 heptane:isopropanol, 0.80 mL/min, 220 nm, 27.93 min (*S* enantiomer), 29.63 min (*R* enantiomer)

$[\alpha]_D^{24}$ = + 111.1° (c = 0.46, $CHCl_3$)

HRMS (ESI): Calculated for $C_{12}H_{12}BrO_2$ $(M+H)^+$: 267.0015, found 267.0019

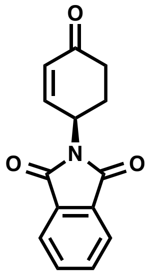

22

22: A round-bottom Biotage® microwave vial (2.0 mL – 5.0 mL size) equipped with a stir bar was charged with **17** (42.6 mg, 0.197 mmol, 1 equivalent), $(\eta^3\text{-}C_3H_5)_2Pd_2Cl_2$ (1.8 mg, 0.005 mmol, 0.025 equivalents), PPh_3 (7.9 mg, 0.03 mmol, 0.15 equivalents), and potassium phthalimide (185.2 mg, 1.0 mmol, 5 equivalents). The vial was sealed with a septum-lined cap and evacuated and backfilled with N_2. Distilled THF (1.0 mL) was added, the pierced septum cap was sealed thoroughly with electrical tape and Parafilm®, and the resulting heterogeneous, cream-colored mixture was stirred for 20 h. The reaction mixture was then diluted with Et_2O (5 mL) and filtered through a large pipet plug of Florisil®, eluting with Et_2O (5 mL). The filtrate was concentrated, and purification of the residue by column chromatography (2:1→1:1 hexanes:EtOAc) afforded **22** (32.6 mg, 0.135 mmol, 69%, 99% ee) as a white solid.

$\mathbf{R}_f$ = 0.34 (1:1 hexanes:EtOAc)

^{1}H NMR (500 MHz; $CDCl_3$): δ 7.89-7.85 (m, 2H), 7.78-7.74 (m, 2H), 6.88 (dt, J = 10.3, 2.1 Hz, 1H), 6.16 (ddd, J = 10.3, 2.9, 1.0 Hz, 1H), 5.22-5.17 (m, 1H), 2.71-2.63 (m, 2H), 2.60-2.51 (m, 1H), 2.23-2.18 (m, 1H)

^{13}C NMR (100 MHz; $CDCl_3$): δ 197.4, 167.7, 149.5, 134.5, 131.8, 130.5, 123.7, 47.2, 36.9, 28.3

IR: 3420, 2919, 2848, 1743, 1691, 1658, 1367, 1341, 1094, 1004, 839, 774, 713 cm^{-1}

Chiral HPLC: IA, 90:10 heptane:isopropanol, 0.80 mL/min, 220 nm, 28.47 min (*S* enantiomer), 31.83 min (*R* enantiomer)

$[\alpha]_D^{24}$ = + 79.5° (c = 0.76, $CHCl_3$)

M.P. = 123-124 °C

HRMS (ESI): Calculated for $C_{14}H_{12}NO_3$ $(M+H)^+$: 242.0812, found 242.0812

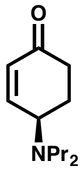

23

23: A round-bottom Biotage® microwave vial (2.0 mL – 5.0 mL size) equipped with a stir bar was charged with **17** (21.4 mg, 0.0990 mmol, 1 equivalent), (η^3-$C_3H_5)_2Pd_2Cl_2$ (0.9 mg, 0.0025 mmol, 0.025 equivalents), and PPh_3 (3.9 mg, 0.015 mmol, 0.15 equivalents). The vial was sealed with a septum-lined cap and evacuated and backfilled with N_2. Degassed (N_2 sparge) CH_2Cl_2 (0.50 mL) was added followed by di-*n*-propylamine (41 μL, 0.30 mmol, 3 equivalents), and the pierced septum cap was sealed thoroughly with electrical tape and Parafilm®. The resulting homogeneous, yellow solution was stirred for 30 min. The reaction mixture was then diluted with Et_2O (5 mL) and poured into saturated aqueous $NaHCO_3$ (5 mL). The phases were separated, and the aqueous phase was extracted with Et_2O (3 x 5 mL). The pooled organics were dried over $MgSO_4$, filtered, and concentrated. Purification of the residue by column chromatography (2:1→1:1 hexanes:EtOAc) afforded **23** (13.3 mg, 0.0680 mmol, 69%, 99% ee) as a clear, yellow, viscous oil.

R$_f$ = 0.41 (1:1 hexanes:EtOAc)

^{1}H NMR (500 MHz; $CDCl_3$): δ 6.98 (dt, *J* = 10.3, 2.0 Hz, 1H), 6.02 (ddd, *J* = 10.3, 2.8, 1.3 Hz, 1H), 3.68-3.64 (m, 1H), 2.57-2.52 (m, 1H), 2.46-2.30 (m, 5H), 2.15-2.11 (m, 1H), 1.92-1.86 (m, 1H), 1.49-1.42 (m, 4H), 0.88 (t, *J* = 7.4 Hz, 6H)

^{13}C NMR (125 MHz; $CDCl_3$): δ 199.6, 156.1, 130.6, 57.7, 53.2, 37.4, 24.6, 22.3, 11.9

IR: 2917, 2892, 2832, 2775, 1773, 1444, 1360, 1189, 1063 cm^{-1}

Chiral HPLC: AD-H, 99:1 heptane:isopropanol, 0.50 mL/min, 220 nm, 16.00 min (*R* enantiomer), 18.61 min (*S* enantiomer)

$[\alpha]_D^{23}$ = + 131.3° (c = 1.33, $CHCl_3$)

HRMS (ESI): Calculated for $C_{12}H_{22}NO$ $(M+H)^+$: 196.1696, found 196.1697

Note: Although spectroscopic data could be obtained in $CDCl_3$/$CHCl_3$, following its concentration from these solvents and upon standing, **23** underwent darkening to a purple oil. Passage of an ethereal solution of this material through a pipet plug of Florisil® returned it to a clear, yellow oil, which was stable after prolonged storage under N_2 in a refrigerator.

This journal is © The Royal Society of Chemistry 2014

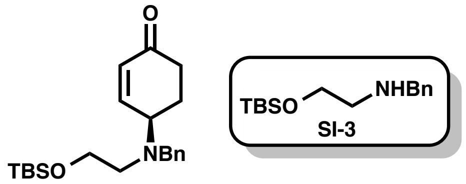

24

24: A round-bottom Biotage® microwave vial (2.0 mL – 5.0 mL size) equipped with a stir bar was charged with **17** (21.1 mg, 0.0976 mmol, 1 equivalent), (η^3-C_3H_5)$_2$$Pd_2Cl_2$ (0.9 mg, 0.0025 mmol, 0.025 equivalents), and PPh_3 (3.9 mg, 0.015 mmol, 0.15 equivalents). The vial was sealed with a septum-lined cap and evacuated and backfilled with N_2. Degassed (N_2 sparge) CH_2Cl_2 (0.50 mL) was added followed by *N*-benzyl-2-((*tert*-butyldimethylsilyl)oxy)ethan-1-amine[13] (**SI-3**, *vide supra*, 79.6 mg, 0.30 mmol, 3 equivalents, *via* tared 100 μL syringe). The pierced septum cap was sealed thoroughly with electrical tape and Parafilm®. The resulting homogeneous, yellow solution was stirred for 30 min. The reaction mixture was then diluted with Et_2O (5 mL) and poured into saturated aqueous $NaHCO_3$ (5 mL). The phases were separated, and the aqueous phase was extracted with Et_2O (3 x 5 mL). The pooled organics were dried over $MgSO_4$, filtered, and concentrated. Purification of the residue by column chromatography (4:1 hexanes:EtOAc) afforded **24** (29.8 mg, 0.0829 mmol, 85%, 87% ee) as a clear, yellow, viscous oil.

R_f = 0.32 (2:1 hexanes:EtOAc)

^{1}H NMR (500 MHz; $CDCl_3$): δ 7.38-7.30 (m, 4H), 7.27-7.24 (m, 1H), 7.03 (dt, *J* = 10.3, 2.0 Hz, 1H), 6.04 (ddd, *J* = 10.3, 2.7, 1.3 Hz, 1H), 3.81-3.66 (m, 3H), 3.62 (td, *J* = 6.3, 1.4 Hz, 2H), 2.67 (t, *J* = 6.4 Hz, 2H), 2.54 (dddd, *J* = 16.4, 4.0, 2.9, 1.2 Hz, 1H), 2.33-2.21 (m, 2H), 1.98-1.90 (m, 1H), 0.87 (s, 9H), 0.02 (s, 6H)

^{13}C NMR (125 MHz; $CDCl_3$): δ 199.4, 155.6, 140.0, 130.9, 128.51, 128.48, 127.2, 63.2, 57.7, 56.1, 52.9, 37.2, 26.0, 24.8, 18.4, -5.23, -5.24

IR: 2986, 2912, 2888, 2816, 1662, 1450, 1362, 1236, 1085, 925, 825, 766, 720, 688 cm^{-1}

Chiral HPLC: AD-H, 98:2 heptane:isopropanol, 0.30 mL/min, 220 nm, 13.49 min (*R* enantiomer), 14.55 min (*S* enantiomer)

[13] D. McKerrecher, K. G. Pike, M. J. Waring, "Heteroaryl benzamide derivatives for use as GLK activations in the treatment of diabetes." U.S. Patent US 2009/0105214 A1, April 23, **2009**.

[α]$_D^{24}$ = + 41.4° (c = 0.49, $CHCl_3$)

HRMS (ESI): Calculated for $C_{21}H_{34}NO_2Si$ $(M+H)^+$: 360.2353, found 360.2351

Note: **24** was obtained containing a small amount (*ca*. 6%, ^{1}H NMR) of an unidentified, possibly aromatic impurity.

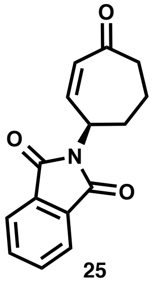

25

25: A round-bottom Biotage® microwave vial (2.0 mL – 5.0 mL size) equipped with a stir bar was charged with **18** (23.0 mg, 0.0999 mmol, 1 equivalent), $(\eta^3\text{-}C_3H_5)_2Pd_2Cl_2$ (0.9 mg, 0.0025 mmol, 0.025 equivalents), dppf (1,1'-bis(diphenylphosphino)ferrocene, 4.2 mg, 0.0075 mmol, 0.075 equivalents), and potassium phthalimide (92.6 mg, 0.5 mmol, 5 equivalents). The vial was sealed with a septum-lined cap and evacuated and backfilled with N_2. Distilled THF (0.5 mL) was added, the pierced septum cap was sealed thoroughly with electrical tape and Parafilm®, and the resulting heterogeneous, golden yellow mixture was stirred for 3 h. The reaction mixture was then diluted with Et_2O (5 mL) and filtered through a large pipet plug of Florisil®, eluting with Et_2O (5 mL). The filtrate was concentrated, and purification of the residue by column chromatography (4:1→2:1 hexanes:EtOAc) afforded **25** (16.1 mg, 0.0631 mmol, 63%, 94% ee) as a white solid.

R$_f$ = 0.36 (1:1 hexanes:EtOAc)

^{1}H NMR (400 MHz; $CDCl_3$): δ 7.89-7.84 (m, 2H), 7.78-7.73 (m, 2H), 6.47 (ddd, *J* = 12.5, 3.2, 1.3 Hz, 1H), 6.09 (dd, *J* = 12.5, 2.9 Hz, 1H), 5.16 (ddt, *J* = 11.3, 4.8, 3.1 Hz, 1H), 2.74-2.68 (m, 2H), 2.52-2.43 (m, 1H), 2.10-1.93 (m, 3H).

^{13}C NMR (100 MHz; $CDCl_3$): δ 202.7, 167.6, 144.2, 134.5, 131.8, 131.6, 123.7, 50.7, 43.1, 31.7, 20.2

IR: 2891, 1745, 1682, 1646, 1450, 1368, 1154, 1091, 883, 791, 711 cm^{-1}

Chiral HPLC: IA, 90:10 heptane:isopropanol, 0.80 mL/min, 220 nm, 32.73 min (*S* enantiomer), 42.0 min (*R* enantiomer)

[α]$_D^{23}$ = + 47.5° (c = 1.2, $CHCl_3$)

M.P. = 147-148 °C

HRMS (ESI): Calculated for $C_{15}H_{14}NO_3$ $(M+H)^+$: 256.0968, found 256.0971

e) Synthesis of Compound 26.

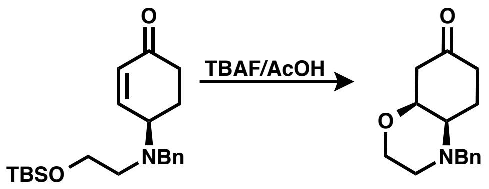

24
87% ee

26
85% ee

26: To a ½-dram vial equipped with a small stir bar was added TBAF·3H_2O (101.8 mg, 0.32 mmol, 4 equivalents), THF (0.80 mL), and AcOH (4.6 μL, 0.08 mmol, 1 equivalent). The mixture was cooled to 0 °C and stirred for 5 min, at which point it was transferred *via* syringe to a pre-cooled (0 °C) 3-dram vial containing neat **24** (29.0 mg, 0.0806 mmol, 1 equivalent, 87% ee) and a stir bar. The homogeneous, yellow solution was stirred for 1 h at this temperature, by which time its appearance had turned to brown and homogeneous. The reaction mixture was warmed to 23 °C and stirred for 1 h, by which time its appearance had turned to purple and homogeneous. The reaction mixture was then diluted with Et_2O (5 mL) and poured into saturated aqueous $NaHCO_3$ (5 mL), using some water (*ca.* 5 mL) to transfer. The phases were separated, and the aqueous phase was extracted with Et_2O (3 x 5 mL). The pooled organics were dried over $MgSO_4$, filtered, and concentrated. Purification of the residue by column chromatography (2:1→1:1 hexanes:EtOAc) afforded **26** (15.2 mg, 0.0620 mmol, 77%, 85% ee) as a clear, yellow, viscous oil and as a single diastereomer (NMR).

R*f* = 0.21 (2:1 hexanes:EtOAc)

^{1}H NMR (400 MHz; $CDCl_3$): δ 7.38-7.25 (m, 5H), 4.09-4.06 (br s, 1H), 3.87 (ddd, *J* = 11.3, 3.6, 2.3 Hz, 1H), 3.71-3.62 (m, 3H), 3.02-2.99 (m, 1H), 2.73-2.18 (m, 7H), 1.92-1.85 (m, 1H).

^{13}C NMR (100 MHz; CD_3CN): δ 209.7, 140.0, 129.7, 129.2, 127.9, 78.3, 67.7, 59.3, 57.4, 46.6, 46.4, 39.1, 17.3 (Not all carbons could be resolved in $CDCl_3$; as a result, the spectrum was acquired in CD_3CN)

IR: 2916, 2864, 2819, 1695, 1432, 1333, 1149, 1116, 1092, 1075, 1049, 735, 690 cm^{-1}

Chiral HPLC: AD-H, 95:5 heptane:isopropanol, 0.80 mL/min, 220 nm, 15.66 min (**26**), 20.37 min (*ent*-**26**)

Electronic Supplementary Material (ESI) for Chemical Science
This journal is © The Royal Society of Chemistry 2014

$[\alpha]_D^{23}$ = – 30.4° (c = 1.0, $CHCl_3$)

HRMS (ESI): Calculated for $C_{15}H_{20}NO_2$ $(M+H)^+$: 246.1489, found 246.1484

Note: The product is assigned as the *cis* diastereomer on the basis of a presumed pseudoaxial attack of oxygen, in accordance with literature precedent.[14]

f) Synthesis of γ-Hydroxycycloalkenones 1, 5, and 27.

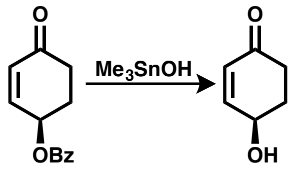

17
99% ee

1
99% ee

1: A round-bottom Biotage® microwave vial (2.0 mL – 5.0 mL size) equipped with a stir bar was charged with **17** (41.7 mg, 0.193 mmol, 1 equivalent) and Me_3SnOH (72.3 mg, 0.40 mmol, 2.1 equivalents). The vial was sealed with a septum-lined cap, and then it was evacuated and backfilled with N_2. Freshly distilled 1,2-dichloroethane (0.50 mL) was added, and the pierced septum cap was sealed thoroughly with electrical tape and Parafilm®. The vessel was heated to 80 °C for 14 h, at which point it was cooled to room temperature, diluted with EtOAc (5 mL), and applied to a column of Florisil® (8 cm x 3 cm). The column was eluted with EtOAc (50 mL), and the solution was concentrated. Preparative thin-layer chromatography (1:2 hexanes:EtOAc) afforded recovered **17** (11.3 mg, 0.0522 mmol, 27% recovery) and **1** (9.2 mg, 0.082 mmol, 43%, 58% based on 73% conversion) as a clear, colorless oil. Chiral HPLC analysis of recovered **17** indicated 99% ee.

^{1}H NMR (500 MHz; $CDCl_3$): δ 6.93 (ddd, *J* = 10.2, 2.4, 1.7 Hz, 1H), 5.98 (ddd, *J* = 10.2, 2.0, 1.1 Hz, 1H), 4.59 (m, 1H), 2.62-2.56 (m, 1H), 2.42-2.33 (m, 2H), 2.04-1.96 (m, 2H)

^{13}C NMR (125 MHz; $CDCl_3$): 199.0, 153.0, 129.4, 66.5, 35.5, 32.6

$[\alpha]_D^{25}$ = + 102.6° (c = 0.92, $CHCl_3$), Lit: + 110° (c = 0.2, $CHCl_3$)[15]

Analytical data matched literature data.[15,16]

---

[14] E. Vásquez, A. Galindo, D. Gnecco, S. Bernès, J. L. Terán, R. G. Enríquez, *Tetrahedron: Asymmetry,* **2001**, *12*, 3209.
[15] A. S. Demir, O. Sesenoglu, *Org. Lett.*, **2002**, *4*, 2021.
[16] G. Dickmeiss, V. De Sio, J. Udmark, T. B. Poulsen, V. Marcos, K. A. Jørgensen, *Angew. Chem. Int. Ed.*, **2009**, *48*, 6650.

To determine the *ee* of **1**, a portion of the material obtained was converted to **17** as follows: to a solution of **1** (3.9 mg, 0.035 mmol, 1 equivalent) in CH_2Cl_2 (0.35 mL) at 0 °C was added pyridine (28 μL, 0.35 mmol, 10 equivalents) and benzoyl chloride (20 μL, 0.17 mmol, 5 equivalents). The solution was stirred for 5 min, and then it was warmed to 23 °C. After 1 h 30 min, the reaction mixture was diluted with Et_2O (1 mL) and water (1 mL) and transferred to a test tube containing pH 7 buffer (1 mL). The phases were separated, and the aqueous phase was extracted with Et_2O (3 x 1 mL). The pooled organics were dried over $MgSO_4$, filtered, and concentrated. Purification of the residue by preparative thin-layer chromatography (2:1 hexanes:EtOAc) delivered **17** (4.4 mg, 0.020 mmol, 59%) as a clear, colorless oil. Analytical data (^{1}H NMR) matched that reported, and chiral HPLC analysis indicated 99% ee.

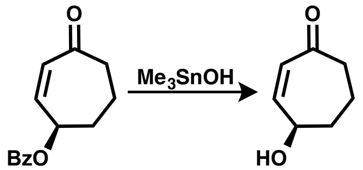

18
99% ee
5
99% ee

5: A round-bottom Biotage® microwave vial (2.0 mL – 5.0 mL size) equipped with a stir bar was charged with **18** (46.8 mg, 0.203 mmol, 1 equivalent) and Me_3SnOH (72.3 mg, 0.40 mmol, 2.0 equivalents). The vial was sealed with a septum-lined cap, and then it was evacuated and backfilled with N_2. Freshly distilled 1,2-dichloroethane (0.50 mL) was added, and the pierced septum cap was sealed thoroughly with electrical tape and Parafilm®. The vessel was heated to 80 °C for 14 h, at which point it was cooled to room temperature, diluted with EtOAc (5 mL), and applied to a column of Florisil® (8 cm x 3 cm). The column was eluted with EtOAc (50 mL), and the solution was concentrated. Preparative thin-layer chromatography (1:2 hexanes:EtOAc) afforded recovered **18** (16.1 mg, 0.0699 mmol, 34% recovery) and **5** (13.3 mg, 0.105 mmol, 52%, 79% based on 66% conversion) as a clear, colorless oil. Chiral HPLC analysis of recovered **18** indicated 99% ee.

^{1}H NMR (500 MHz; $CDCl_3$): δ 6.57 (ddd, J = 12.5, 3.0, 1.1 Hz, 1H), 5.95 (ddd, J = 12.5, 2.2, 0.8 Hz, 1H), 4.59-4.55 (m, 1H), 2.63-2.53 (m, 2H), 2.23-2.10 (m, 2H), 1.87-1.79 (m, 3H).

^{13}C NMR (125 MHz; $CDCl_3$): 203.4, 149.3, 130.0, 70.6, 43.2, 35.3, 18.4

[α]$_D$^{24} = + 119.2° (c = 0.67, $CHCl_3$), Lit: + 113.9° (c = 0.54, $CHCl_3$)[17]

Analytical data matched literature data.[17]

To determine the *ee* of **5**, a portion (4.4 mg, 0.035 mmol) was benzoylated in the same manner as **1**. Purification by preparative thin-layer chromatography (2:1 hexanes:EtOAc) delivered **18** (3.9 mg, 0.017 mmol, 49%), and chiral HPLC analysis indicated 99% ee.

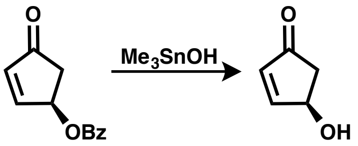

16
99% ee

27
95% ee

27: A round-bottom Biotage® microwave vial (2.0 mL – 5.0 mL size) equipped with a stir bar was charged with **16** (40.4 mg, 0.200 mmol, 1 equivalent) and Me_3SnOH (54.2 mg, 0.30 mmol, 1.5 equivalents). The vial was sealed with a septum-lined cap, and then it was evacuated and backfilled with N_2. Freshly distilled 1,2-dichloroethane (0.50 mL) was added, and the pierced septum cap was sealed thoroughly with electrical tape and Parafilm®. The vessel was heated to 80 °C for 14 h, at which point it was cooled to room temperature, diluted with EtOAc (5 mL), and applied to a column of Florisil® (8 cm x 3 cm). The column was eluted with EtOAc (50 mL), and the solution was concentrated. Preparative thin-layer chromatography (1:2 hexanes:EtOAc) afforded recovered **16** (14.3 mg, 0.0707 mmol, 35% recovery) and **27** (8.7 mg, 0.089 mmol, 44%, 69% based on 65% conversion) as a clear, colorless oil. Chiral HPLC analysis of recovered **16** indicated 99% ee.

^{1}H NMR (500 MHz; $CDCl_3$): δ 7.57 (dd, *J* = 5.7, 2.3 Hz, 1H), 6.23 (dd, *J* = 5.7, 1.3 Hz, 1H), 5.07-5.05 (m, 1H), 2.78 (dd, *J* = 18.5, 6.1 Hz, 1H), 2.36-2.19 (br s, 1H), 2.28 (dd, *J* = 18.5, 2.2 Hz, 1H).

^{13}C NMR (125 MHz; $CDCl_3$): 206.7, 163.3, 135.4, 70.6, 44.4

[α]$_D$^{24} = + 51.8° (c = 0.87, $CHCl_3$), Lit: + 57° (c = 1, $CHCl_3$)[16]

Analytical data matched literature data.[16]

[17] M. Kawasumi, N. Kanoh, Y. Iwabuchi, *Org. Lett.*, **2011**, *13*, 3620.

To determine the *ee* of **27**, a portion (3.4 mg, 0.035 mmol) was benzoylated in the same manner as **1**. Purification by preparative thin-layer chromatography (2:1 hexanes:EtOAc) delivered **16** (4.4 mg, 0.022 mmol, 62%), and chiral HPLC analysis (*vide supra*) indicated 95% ee.

g) Total Synthesis of (–)-Tricholomenyn A (28).

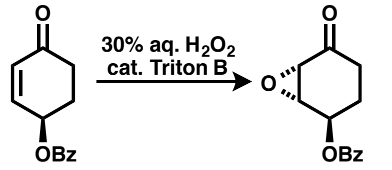

17
99% ee

38
>19:1 dr
99% ee

38: Based on the procedure of Kitahara and co-worker.[18] To a solution of **17** (783 mg, 3.62 mmol, 1 equivalent) in THF (4.8 mL) at 0 °C was added hydrogen peroxide (1.12 mL of a *ca.* 30% aqueous solution, 10.86 mmol, 3 equivalents) followed by Triton B (benzyltrimethylammonium hydroxide, 0.14 mL of a 40% aqueous solution, 0.36 mmol, 0.10 equivalent). The resulting light yellow reaction mixture was stirred at 0 °C for 1 h, and then it was diluted with saturated aqueous $NaHCO_3$ (5 mL) and Et_2O (10 mL). The phases were separated, and the aqueous phase was extracted with Et_2O (3 x 10 mL). The pooled organics were dried over $MgSO_4$, filtered, and concentrated. The residue was purified by column chromatography (4:1 hexanes:EtOAc) to afford **38** (665 mg, 2.86 mmol, 79% yield) as a colorless oil.

$\mathbf{R}_f$ = 0.36 (4:1 hexanes:EtOAc)

^{1}H NMR (400 MHz; $CDCl_3$): δ 8.02-8.00 (m, 2H), 7.59 (t, J = 7.4 Hz, 1H), 7.47-7.43 (m, 2H), 5.76 (q, J = 3.3 Hz, 1H), 3.81 (ddd, J = 3.8, 2.9, 0.9 Hz, 1H), 3.39 (d, J = 3.8 Hz, 1H), 2.57-2.27 (m, 3H), 2.07-2.00 (m, 1H)

^{13}C NMR (100 MHz, $CDCl_3$): 203.9, 165.7, 133.7, 129.8, 129.4, 128.7, 67.6, 55.4, 55.2, 32.0, 22.3.

IR: 3064, 2951, 1716, 1601, 1585, 1492, 1452, 1408, 1317, 1267, 1178, 1108, 1070, 1028, 923, 847, 791, 764 cm^{-1}

[18] T. Tachihara, T. Kitahara, *Tetrahedron*, **2003**, *59*, 1773.

Chiral HPLC: Chiracel OJ-H, 90:10 heptane:isopropanol, 0.80 mL/min, 220 nm, 11.07 min (**38**), 11.94 min (*ent*-**38**)

$[\alpha]_D^{23}$ = − 4.5° (c = 0.75, $CHCl_3$). For *ent*-**38**, $[\alpha]_D^{23}$ = + 3.6° (c = 0.75, $CHCl_3$)

HRMS (ESI): Calculated for $C_{13}H_{12}NaO_4$ $(M+Na)^+$: 255.0628, found 255.0629

Note: On some occasions, a second addition of Triton B (0.10 equiv) was necessary to initiate the reaction.

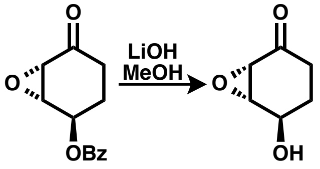

38 37

37: A solution of **38** (656 mg, 2.82 mmol, 1 equivalent) in MeOH (22 mL) was cooled to 0 °C and treated with a freshly prepared, pre-cooled (0 °C) solution of $LiOH{\cdot}H_2O$ (178 mg, 4.24 mmol, 1.5 equivalents) in MeOH (6 mL). The reaction mixture was stirred at 0 °C for 1 h before it was quenched by the addition of pH 7 buffer (25 mL) and poured into Et_2O (50 mL). The phases were separated, and the aqueous phase was extracted with Et_2O (3 x 50 mL). The pooled organics were dried over $MgSO_4$, filtered, and concentrated, and the residue was purified by column chromatography (4:1→1:1 hexanes:EtOAc) to afford **37** (302 mg, 2.36 mmol, 83%) as a clear, colorless oil.

$\mathbf{R}_f$ = 0.19 (1:1 hexanes:EtOAc)

^{1}H NMR (400MHz, $CDCl_3$): 4.57-4.54 (m, 1H), 3.60-3.58 (m, 1H), 3.31 (d, 3.8 Hz, 1H), 2.42-2.39 (m, 2H), 2.23-2.14 (m, 1H), 1.84-1.77 (m, 1H)

^{13}C NMR (125MHz, $CDCl_3$): 205.2, 64.6, 58.0, 55.0, 31.6, 25.7

IR (film): 3418 (br), 2944, 1714, 1427, 1338, 1257, 1228, 1187, 1060, 985, 941, 871, 804, 785, 769 cm^{-1}

$[\alpha]_D^{23}$ = − 102.6 (c = 0.29, $CHCl_3$). For *ent*-**37**, $[\alpha]_D^{23}$ = + 104.6 (c = 0.29, $CHCl_3$)

HRMS (ESI): Calculated for $C_6H_9O_3$ $(M+H)^+$: 126.0546, found 126.0545

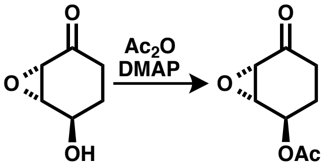

37 39

39: Based on the procedure of Figueredo, Bayón, and co-workers.[19] To a solution of **37** (123 mg, 0.960 mmol, 1 equivalent) in MeCN (4.8 mL) at 0 °C was added DMAP (129 mg, 1.06 mmol, 1.1 equivalents) followed immediately by Ac_2O (0.36 mL, 3.84 mmol, 4 equivalents). The reaction mixture was stirred for 1-2 min at 0 °C, and then the cooling bath was removed and the reaction mixture stirred for 10 min. It was then poured into a mixture of Et_2O (10 mL) and saturated aqueous $NaHCO_3$ (10 mL), diluting this mixture with a small amount of water. The phases were separated, and the aqueous phase was extracted with Et_2O (2 x 10 mL). The pooled organics were dried over $MgSO_4$, filtered, and concentrated. The residue was purified by column chromatography (2:1 hexanes:EtOAc) to afford **39** (144 mg, 0.846 mmol, 88%) as a clear, light yellow oil.

R_f = 0.35 (2:1 hexanes:EtOAc)

^{1}H NMR (500 MHz; $CDCl_3$): δ 5.50-5.47 (m, 1H), 3.67-3.64 (m, 1H), 3.31 (d, J = 3.8 Hz, 1H), 2.47-2.41 (m, 1H), 2.37-2.30 (m, 1H), 2.25-2.18 (m, 1H), 2.09 (s, 3H), 1.90-1.84 (m, 1H)

^{13}C NMR (125 MHz; $CDCl_3$): δ 203.9, 170.3, 67.1, 55.2, 55.0, 31.8, 22.1, 21.1

IR: 2952, 1716, 1442, 1373, 1233, 1130, 1040, 964, 863, 796, 762 cm^{-1}

$[\alpha]_D^{22}$ = − 55.0° (c = 0.40, $CHCl_3$)

HRMS (ESI): Calculated for $C_8H_{10}NaO_4$ $(M+Na)^+$: 193.0471, found 193.0466

[19] Toribio, G., Marjanet, G., Alibés, R., de March, P., Font, J., Bayón, P., Figueredo, M. *Eur. J. Org. Chem.* **2011**, 1534.

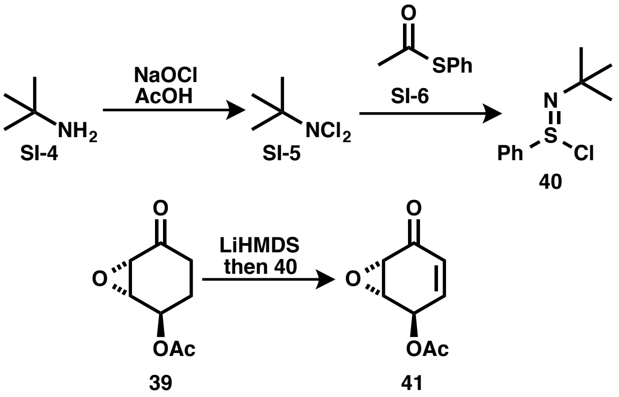

Preparation of Mukaiyama reagent **40**: The synthesis of *N*,*N*-dichloro-*tert*-butylamine (**SI-5**) was adapted from the literature procedure for *tert*-butyl hypochlorite.[20] To a 250 mL Erlenmeyer flask equipped with a stir bar was added commercial, household bleach (*ca.* 5-10% NaOCl, 100 mL). The flask was cooled to 0 °C, and the fume hood lights were turned off. AcOH (4.9 mL, 86 mmol, 2.2 equivalents) was added dropwise, followed by *tert*-butylamine (**SI-4**, 4.1 mL, 39 mmol, 1 equivalent, *added cautiously*). The reaction mixture was stirred for 5 min, and then it was poured into a separatory funnel containing CH_2Cl_2 (100 mL). At this point, the fume hood lights were turned on. The aqueous phase was discarded, and the organic phase was washed with saturated aqueous $NaHCO_3$ (50 mL) then water (50 mL), dried over $MgSO_4$, filtered, and concentrated to provide the volatile **SI-5** (2.23 g, 15.7 mmol, 40%) as a light yellow oil. **SI-5** was used immediately in the next step, which was adapted from the literature.[21] **SI-5** (2.23 g, 15.7 mmol, 1.2 equivalents) was dissolved in PhH (13 mL), and *S*-phenyl thioacetate (**SI-6**, 1.77 mL, 13.1 mmol, 1 equivalent) was added. The flask was heated to 80 °C and then sealed with a plastic cap. After 1 h 15 min, TLC (5:1 hexanes:Et_2O) indicated complete consumption of **SI-6**. The flask was cooled to room temperature and concentrated. Volatile material was removed azeotropically with PhH (2 x 20 mL). The residue was dissolved in CH_2Cl_2, filtered through a large pipet column of sand, and concentrated to deliver crude **40** (3.18 g, 14.7 mmol, >100%), which was used without further purification.

[20] M. J. Mintz, C. Walling, *Org. Synth.* **1969**, *49*, 9.
[21] T. Mukaiyama, J.-i. Matsuo, M. Yanagisawa, *Chem. Lett.*, **2000**, 1072.

41: The procedure was adapted from the literature.[22] Ketone **39** (42.5 mg, 0.250 mmol, 1 equivalent) was dried by azeotropic removal of water with PhH (2 x 2 mL), and then it was dissolved in freshly distilled THF (2.5 mL). The solution was cooled to – 78 °C. Freshly prepared LiHMDS (0.70 mL of a solution prepared by adding *n*BuLi [0.43 mL of a 2.36 M solution in hexanes] to freshly distilled HMDS [0.23 mL] in THF [2.0 mL] at – 78 °C, stirring for 30 min, then warming to 23 °C and using immediately, corresponds to 0.263 mmol, 1.05 equivalents) was added, and the clear, colorless enolate solution was stirred at – 78 °C for 30 min. A solution of reagent **40** (162 mg, 0.75 mmol, 3 equivalents) in THF (2 x 300 μL) was added *via* syringe, affording a neon green/yellow reaction mixture. After stirring for 30 min at – 78 °C, the reaction mixture was poured into a mixture of pH 7 buffer (10 mL) and Et_2O (10 mL). The phases were separated, and the aqueous phase was extracted with Et_2O (2 x 10 mL). The pooled organics were washed with water (10 mL) then brine (10 mL), dried over $MgSO_4$, filtered, and concentrated. The residue was purified by column chromatography (4:1 hexanes:EtOAc) to afford **41** (24.5 mg, 0.145 mmol, 58%) as a viscous yellow oil that partially solidified over time.

$\mathbf{R_f}$ = 0.35 (2:1 hexanes:EtOAc)

^{1}H NMR (500 MHz; $CDCl_3$): δ 6.60 (ddd, *J* = 10.6, 4.7, 2.5 Hz, 1H), 6.10 (dt, *J* = 10.6, 1.5 Hz, 1H), 5.76 (dq, *J* = 4.7, 1.1 Hz, 1H), 3.74 (ddd, *J* = 3.6, 2.5, 1.2 Hz, 1H), 3.51-3.49 (m, 1H), 2.15 (s, 3H)

^{13}C NMR (125 MHz; $CDCl_3$): δ 192.7, 169.9, 139.5, 129.1, 64.0, 55.4, 53.0, 20.8

IR: 2919, 2850, 1739, 1689, 1372, 1220, 1024, 794 cm^{-1}

$[\alpha]_D^{22}$ = – 377.3° (c = 0.80, $CHCl_3$)

M.P. = 85-86 °C (solid material obtained *via* IBX oxidation)

MS (ESI): Calculated for $C_8H_7O_4$: 168.04, found 167.15 $(M-H)^-$

Notes:

1) Ketone **39** and enone **41** could not be separated from each other by chromatography, and **41** was obtained in this manner containing a small amount (*ca*. 3%, ^{1}H NMR) of unreacted **39**. The material also contained minor amounts of apparent byproducts from reagent **40** (*ca*. 10%, ^{1}H NMR).

[22] T. Mukaiyama, J.-i. Matsuo, H. Kitagawa, *Chem. Lett.*, **2000**, 1250.

2) The oxidation **39→41** could also be performed with IBX and catalytic *p*-TsOH·H_2O (0.35 equivalents) in DMSO,[23] although this required an excess of IBX (6-7 equivalents) and extended heating (65 °C, 24 h) to obtain high conversion. These conditions delivered **39** and **41** as the only isolable products but in highly variable yields (25-66%), a result that led us to favor the above procedure.

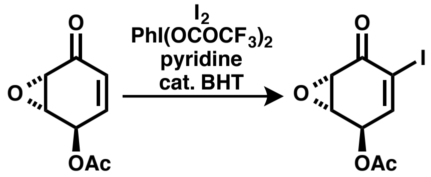

41 **42**

42: Based on literature procedures.[24] A ½-dram vial equipped with a small stir bar was charged with I_2 (43 mg, 0.17 mmol, 1.3 equivalents), $PhI(OCOCF_3)_2$ (72 mg, 0.17 mmol, 1.3 equivalents), CH_2Cl_2 (250 µL), and pyridine (27 µL, 0.34 mmol, 2.6 equivalents), and this mixture was stirred for 15 min. Meanwhile, to a solution of **41** (21.7 mg, 0.129 mmol, 1 equivalent) in CH_2Cl_2 (300 µL) was added BHT (butylated hydroxytoluene, 0.7 mg, 0.003 mmol, 0.065 equivalents). The former solution was transferred to the latter *via* 100 µL syringe, using additional CH_2Cl_2 (100 µL) to rinse the vial and syringe. The latter vial was capped, sealed well with Parafilm®, and wrapped in aluminum foil. After stirring for 24 h, the reaction mixture was diluted with Et_2O (5 mL) and poured into aqueous $Na_2S_2O_3$ (10 mL). The phases were separated, the aqueous phase was extracted with Et_2O (2 x 5 mL), and the pooled organics were dried over $MgSO_4$, filtered, and concentrated. The residue was purified by column chromatography (4:1→2:1 hexanes:EtOAc) to deliver **42** (18.7 mg, 0.0636 mmol, 49%) as a viscous, yellow oil that partially solidified over time.

R*f* = 0.35 (2:1 hexanes:EtOAc)

^{1}H NMR (400 MHz; $CDCl_3$): δ 7.38 (dd, *J* = 5.2, 2.3 Hz, 1H), 5.64 (dt, *J* = 5.2, 1.2 Hz, 1H), 3.78 (ddd, *J* = 3.5, 2.3, 1.3 Hz, 1H), 3.67 (dd, *J* = 3.5, 1.2 Hz, 1H), 2.15 (s, 3H)

^{13}C NMR (100 MHz; $CDCl_3$): δ 187.4, 169.6, 147.7, 104.9, 66.0, 55.2, 51.4, 20.7

IR: 2919, 1738, 1694, 1601, 1369, 1330, 1214, 1025, 963, 936, 904, 874, 781 cm^{-1}

---

[23] K. C. Nicolaou, T. Montagnon, P. S. Baran, Y.-L. Zhong, *J. Am. Chem. Soc.*, **2002**, *124*, 2245.
[24] a) R. Benhida, P. Blanchard, J.-L. Fourrey, *Tetrahedron Lett.*, **1998**, *39*, 6849; b) M. Shoji, H. Imai, M. Mukaida, K. Sakai, H. Kakeya, H. Osada, Y. Hayashi, *J. Org. Chem.*, **2005**, *70*, 79.

[α]D[24] = – 178.9° (c = 0.50, $CHCl_3$), Lit: – 204.4° (c = 0.50, $CHCl_3$)[25a]

Notes:

1) As with **41**, compound **42** prepared in this manner contained a small amount of inseparable, unreacted **39** (*ca.* 3%, ^{1}H NMR) as well as minor byproducts from reagent **40** (*ca.* 10%, ^{1}H NMR).

2) Yields ranging from 49-66% were obtained, using **41** obtained from the Mukaiyama protocol or from IBX oxidation.

Analytical data matched literature data.[25]

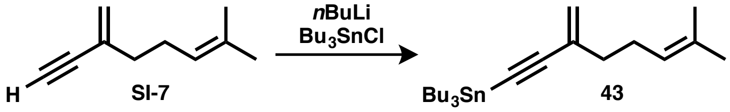

43: To a solution of compound **SI-7** (40.3 mg, 0.30 mmol, 1.0 equivalent, prepared according to the procedure of Wulff and co-workers[26]) in freshly distilled THF (1.0 mL) at – 78 °C was added freshly titrated *n*BuLi (0.125 mL, 2.39 M in hexanes, 0.30 mmol, 1.0 equivalent) dropwise. The solution was stirred at – 78 °C for 15 min, at which point it was warmed to 0 °C by replacing the cooling bath with an ice/water bath. After 15 min, the solution was re-cooled to – 78 °C, and freshly distilled Bu_3SnCl (81.4 µL, 0.30 mmol, 1.0 equivalent) was added dropwise. The cooling bath was removed, and the solution was stirred at room temperature for 30 min. It was then diluted with Et_2O (5 mL) and *quickly* vacuum filtered through a fritted funnel containing a thin pad of SiO_2, into a flame-dried 100 mL flask. The pad was washed with additional Et_2O (15 mL). The filtrate was concentrated, the residue was dissolved in Et_2O, and the solution was filtered through a pipet plug of Celite® into a flame-dried 2-dram vial. The filtrate was concentrated to afford **43** (117 mg, 0.276 mmol, 92%) as a light yellow, clear oil, which was used without further purification.

Note: In our hands, attempts to purify **43** using column chromatography on SiO_2 (hexanes eluent, no deactivation) led to complete destannylation, returning terminal alkyne **SI-7**. However, this could be avoided by purifying **43** using the quick filtration described.

---

[25] a) X. Ma, J. C. Jury, M. G. Banwell, *Tetrahedron Lett.* **2011**, *52*, 2192; b) D. M. Pinkerton, M. G. Banwell, A. C. Willis, *Aust. J. Chem.* **2009**, *62,* 1639.
[26] W. Jiang, M. J. Fuertes, W. D. Wulff, *Tetrahedron*, **2000**, *56*, 2183.

Electronic Supplementary Material (ESI) for Chemical Science
This journal is © The Royal Society of Chemistry 2014

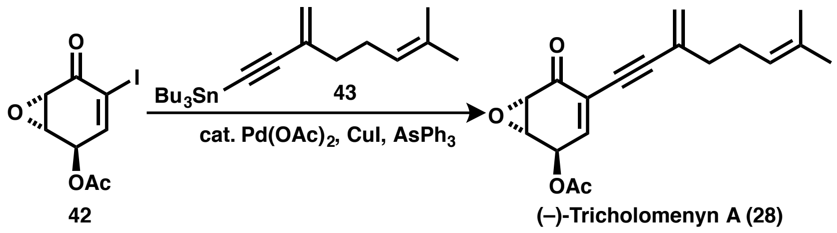

(–)-**Tricholomenyn A (28)**: The procedure of Banwell and co-workers was adapted.[25b] To a solution of **42** (17.5 mg, 0.0595 mmol, 1 equivalent) in distilled THF (1.0 mL) in a 2-dram vial at 0 °C was added **43** (50.4 mg, 0.12 mmol, 2 equivalents) *via* tared 100 µL syringe, yielding a golden yellow, homogeneous solution. To this was added $Pd(OAc)_2$ (1.3 mg, 0.006 mmol, 0.10 equivalent), CuI (1.1 mg, 0.006 mmol, 0.10 equivalent), and $AsPh_3$ (3.7 mg, 0.012 mmol, 0.20 equivalent) all at once under N_2 flow. The reaction mixture was stirred at 0 °C for 1 h 30 min, during which time its appearance changed to olive green and homogeneous. The reaction mixture was then poured into a mixture of Et_2O (20 mL) and pH 7 buffer (5 mL), the phases were separated, and the aqueous phase was extracted with Et_2O (2 x 5 mL). The pooled organics were washed with saturated aqueous NH_4F (2 x 10 mL), then water (10 mL), then brine (10 mL), dried over $MgSO_4$, filtered, and concentrated. The residue was purified by column chromatography (4:1 hexanes:EtOAc) followed by preparative thin-layer chromatography (20:1 CH_2Cl_2:PhH) to deliver **28** (9.9 mg, 0.0330 mmol, 55%, free of the impurities derived from **40** and residual ketone **39**) as a clear, light yellow, viscous oil.

$\mathbf{R}_f$ = 0.31 (4:1 hexanes:EtOAc)

^{1}H NMR (600 MHz; $CDCl_3$): δ 6.75 (dd, *J* = 5.2, 2.5 Hz, 1H), 5.82 (dt, *J* = 5.2, 1.2 Hz, 1H), 5.45 (d, *J* = 1.6 Hz, 1H), 5.35 (d, *J* = 1.4 Hz, 1H), 5.11-5.09 (m, 1H), 3.74 (ddd, *J* = 3.7, 2.4, 1.4 Hz, 1H), 3.60 (dd, *J* = 3.6, 1.0 Hz, 1H), 2.27-2.20 (m, 4H), 2.14 (s, 3H), 1.68 (s, 3H), 1.62 (s, 3H)

^{13}C NMR (125 MHz; $CDCl_3$): δ 189.6, 169.8, 140.5, 132.6, 130.7, 125.2, 123.7, 123.2, 96.1, 82.0, 64.3, 54.9, 53.1, 37.1, 26.8, 25.8, 20.8, 17.9

IR: 2922, 2205, 1742, 1702, 1617, 1443, 1372, 1299, 1219, 1116, 1025, 987, 909, 829, 779 cm^{-1}

$[\alpha]_D^{23}$ = – 234.6° (c = 0.96, CH_2Cl_2), Lit: – 241.3° (c = 1.35, CH_2Cl_2),[25b] – 228° (c = 0.1, CH_2Cl_2),[27] – 235.7° (c = 1.47, CH_2Cl_2),[28] – 237 (c = 0.6, CH_2Cl_2),[29] – 148.1° (c = 0.35, CH_2Cl_2)[30]

HRMS (ESI): Calculated for $C_{18}H_{20}NaO_4$ $(M+Na)^+$: 323.1254, found 323.1255

Analytical data matched literature data.[25b, 27-30]

Note: Yields ranging from 55-60% were obtained using **42** prepared using either the Mukaiyama protocol or IBX oxidation, followed by iodination.

h) Synthesis of Epoxyquinoid Precursors 44 and 45.

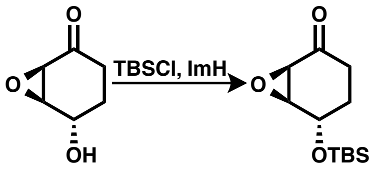

ent-**37** **44**

44: To a solution of ***ent*-37** (43.0 mg, 0.336 mmol, 1 equivalent, prepared as detailed above for **37**, 98% ee) in CH_2Cl_2 (1.3 mL) was added TBSCl (65.8 mg, 0.44 mmol, 1.3 equivalents) and imidazole (35.4 mg, 0.52 mmol, 1.55 equivalents). The reaction mixture was stirred for 16 h, and then it was loaded directly onto an SiO_2 column and eluted with 10:1 hexanes:Et_2O to provide **44** (71.2 mg, 0.294 mmol, 88%) as a clear, colorless oil.

R_f = 0.59 (4:1 hexanes:EtOAc)

^{1}H NMR (400 MHz; $CDCl_3$): δ 4.44 (dd, J = 4.0, 2.8 Hz, 1H), 3.45 (app t, J = 3.1 Hz, 1H), 3.25 (d, J = 3.9 Hz, 1H), 2.42-2.28 (m, 2H), 2.12-2.03 (m, 1H), 1.72-1.66 (m, 1H), 0.88 (s, 9H), 0.11 (s, 3H), 0.09 (s, 3H).

^{13}C NMR (100 MHz; $CDCl_3$): δ 205.0, 65.3, 58.1, 55.0, 31.7, 25.9, 25.6, 18.2, -4.6, -4.7

IR: 2955, 2889, 2859, 1719, 1472, 1444, 1405, 1362, 1336, 1254, 1228, 1187, 1092, 1035, 982, 687, 839, 778 cm^{-1}

$[\alpha]_D^{23}$ = + 58.2° (c = 1.92, $CHCl_3$), Lit: + 57.3° (c = 1.96, $CHCl_3$)[18]

Analytical data matched literature data.[18,31]

---

[27] J. Li, S. Park, R. L. Miller, D. Lee, *Org. Lett.* **2009**, *11*, 571.
[28] T. Kamikubo, K. Ogasawara, *Chem. Commun.*, **1996***, 1679.*
[29] M. W. Miller, C. R. Johnson, *J. Org. Chem.* **1997**, *62*, 1582.
[30] L. Garlaschelli, E. Magistrali, G. Vidari, O. Zuffardi, *Tetrahedron Lett.* **1995**, *36*, 5633.

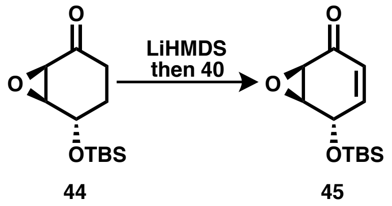

45: To a solution of **44** (26.3 mg, 0.109 mmol, 1 equivalent) at – 78 °C was added freshly prepared LiHMDS (0.15 mL of a solution prepared by adding *n*BuLi [0.45 mL of a 2.20 M solution in hexanes] to freshly distilled HMDS [0.23 mL] in THF [0.50 mL] at – 78 °C, stirring for 30 min, then warming to 23 °C and using immediately, corresponds to 0.13 mmol, 1.2 equivalents). The solution was stirred at – 78 °C for 30 min, at which point a solution of **40** (70 mg, 0.33 mmol, 3 equivalents) in THF (200 μL) was added *via* cannula. Additional THF (2 x 100 μL) was used to rinse the vial containing **40** and the cannula. The reaction mixture was stirred at – 78 °C for 1 h, at which point it was poured into a mixture of pH 7 buffer (3 mL) and Et_2O (5 mL). The phases were separated, and the aqueous phase was extracted with Et_2O (2 x 5 mL). The pooled organics were dried over $MgSO_4$, filtered, and concentrated, and the residue was purified by column chromatography (20:1 hexanes:EtOAc) to afford **45** (17.1 mg, 0.0711 mmol, 66%) as a clear, light yellow oil.

$\mathbf{R}_f$ = 0.59 (4:1 hexanes:EtOAc)

^{1}H NMR (400 MHz; $CDCl_3$): δ 6.52 (ddd, J = 10.5, 4.5, 2.6 Hz, 1H), 5.96 (ddd, J = 10.5, 1.8, 1.2 Hz, 1H), 4.66 (dq, J = 4.5, 1.1 Hz, 1H), 3.65 (ddd, J = 3.6, 2.5, 1.1 Hz, 1H), 3.46 (ddd, J = 3.6, 1.8, 1.1 Hz, 1H), 0.92 (s, 9H), 0.18 (s, 3H), 0.16 (s, 3H).

^{13}C NMR (100 MHz; $CDCl_3$): δ 193.3, 144.5, 126.4, 63.8, 58.5, 53.5, 25.8, 18.3, -4.3, -4.8

IR: 2956, 2931, 2888, 2859, 1692, 1472, 1259, 1090, 876, 856, 839, 824, 806, 779 cm^{-1}

Analytical data matched literature data.[18,32]

---

[31] V. Rodeschini, P. Van de Weghe, E. Salomon, C. Tarnus, J. Eustache, *J. Org. Chem.*, **2005**, *70*, 2409.
[32] M. T. Barros, C. D. Maycock, M. R. Ventura, *Chem. Eur. J.*, **2000**, *6*, 3991.

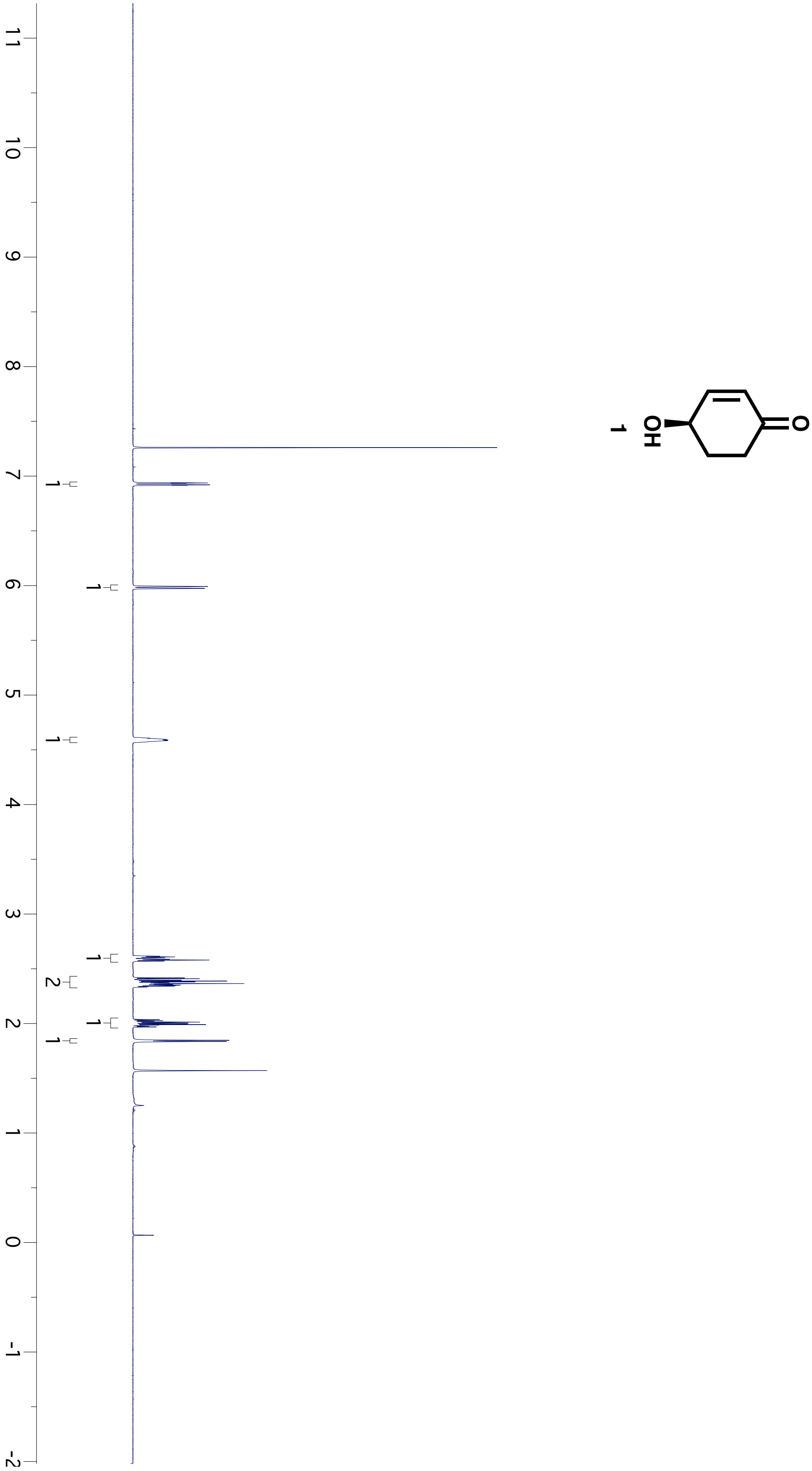

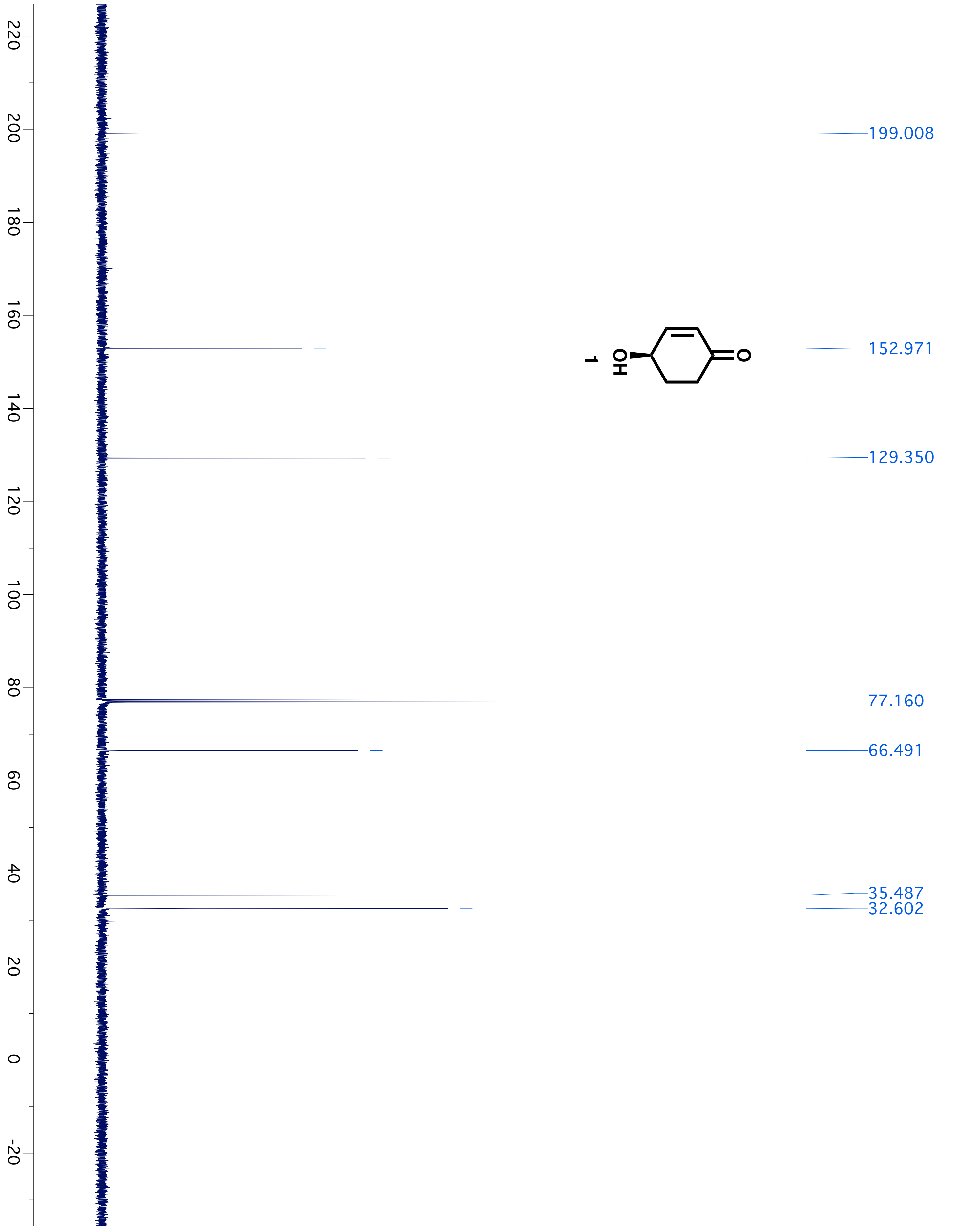
199.008
152.971
129.350
77.160
66.491
35.487
32.602
1
220
200
180
160
140
120
100
80
60
40
20
0
-20

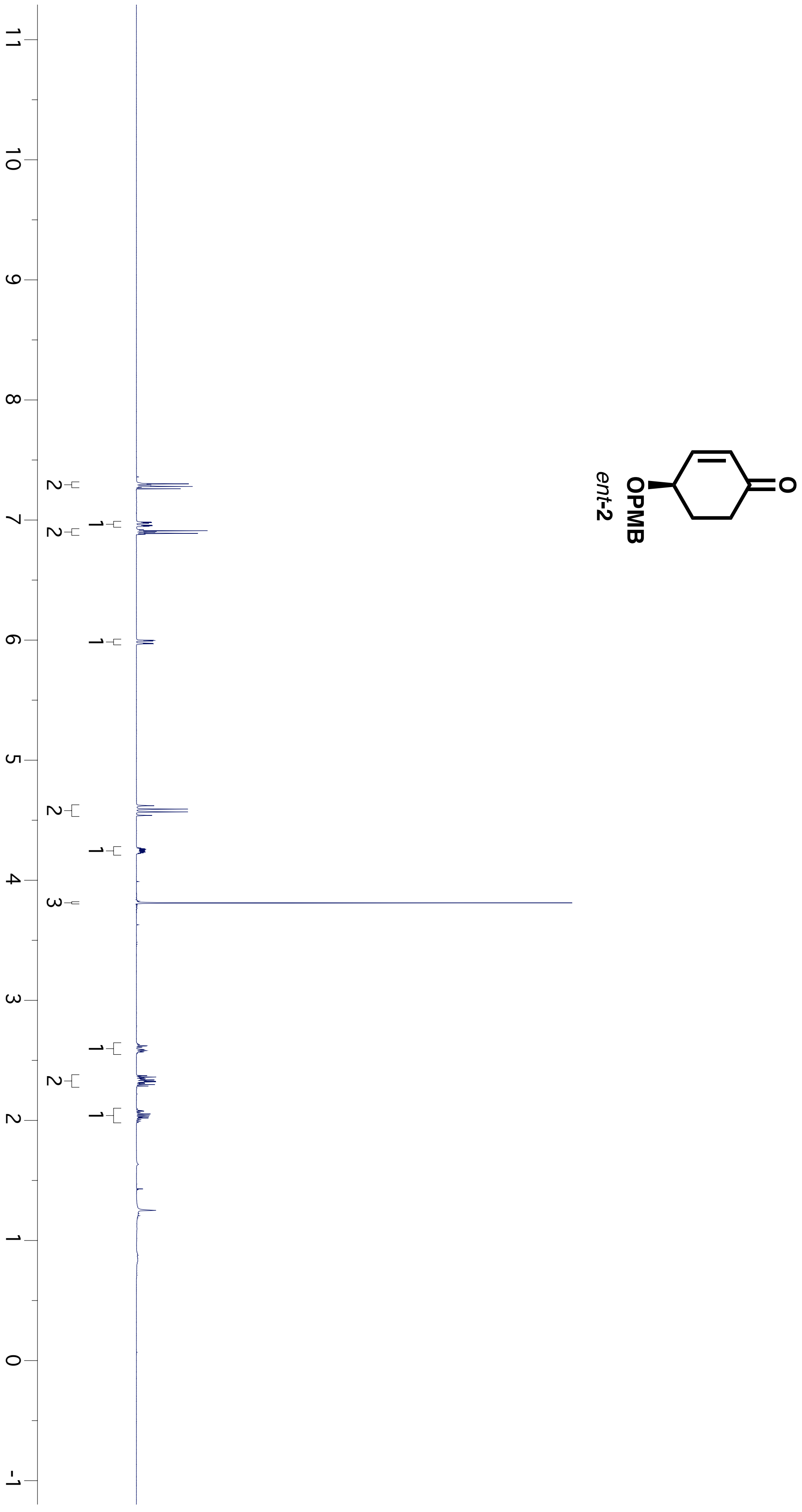
OPMB
ent-2

This journal is © The Royal Society of Chemistry 2014

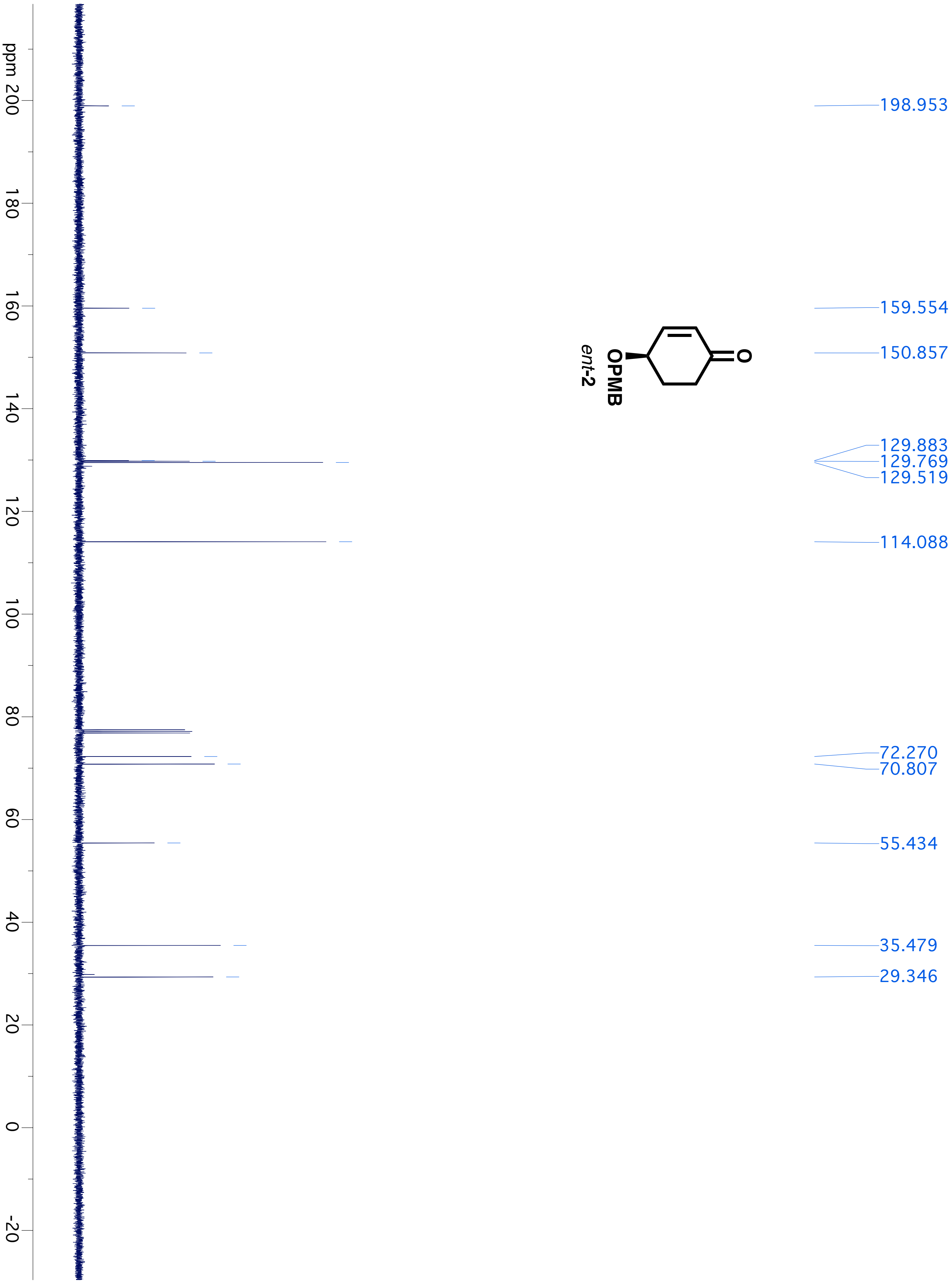

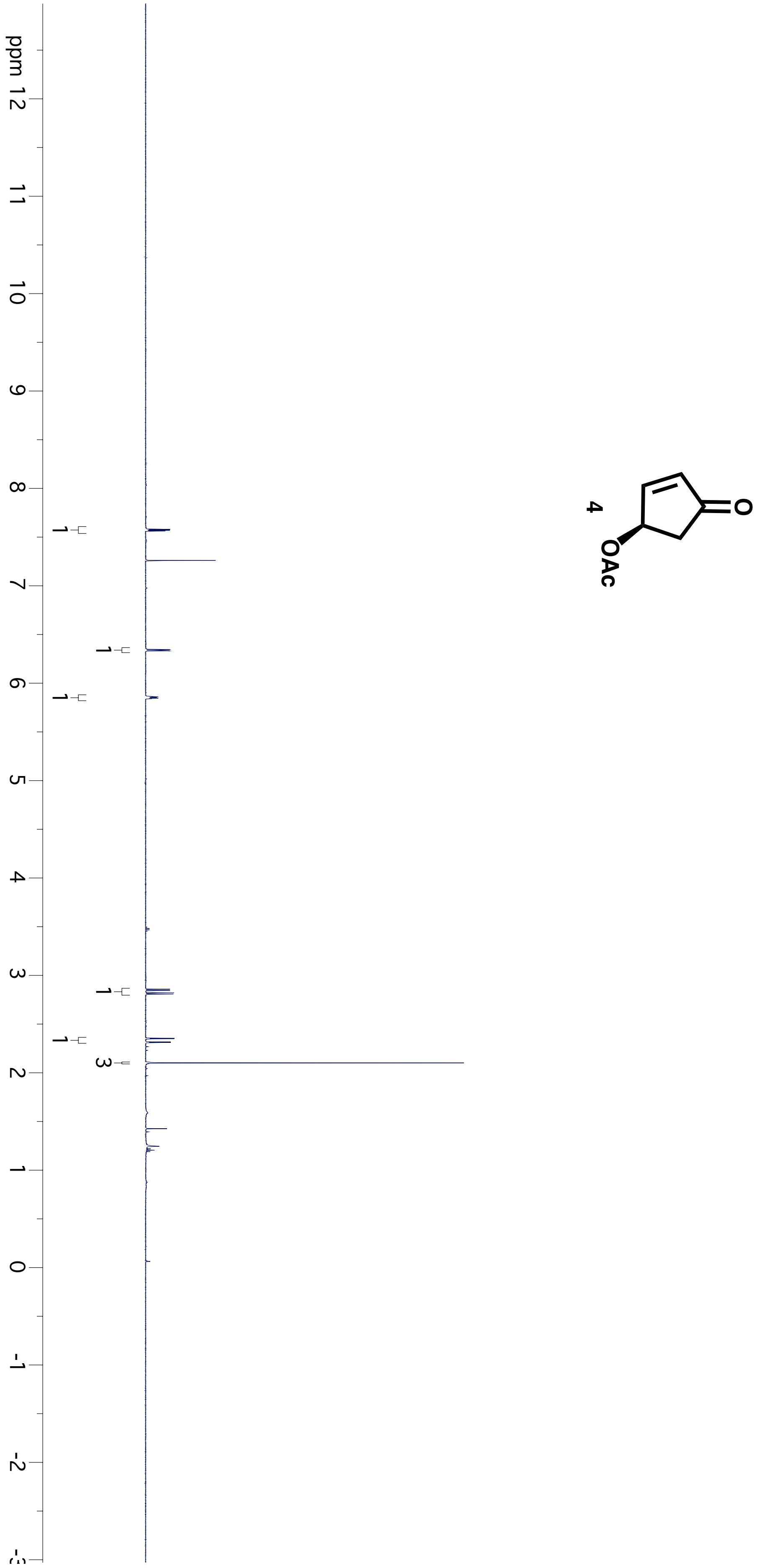
O
OAc
4
ppm
12
11
10
9
8
7
6
5
4
3
2
1
0
-1
-2

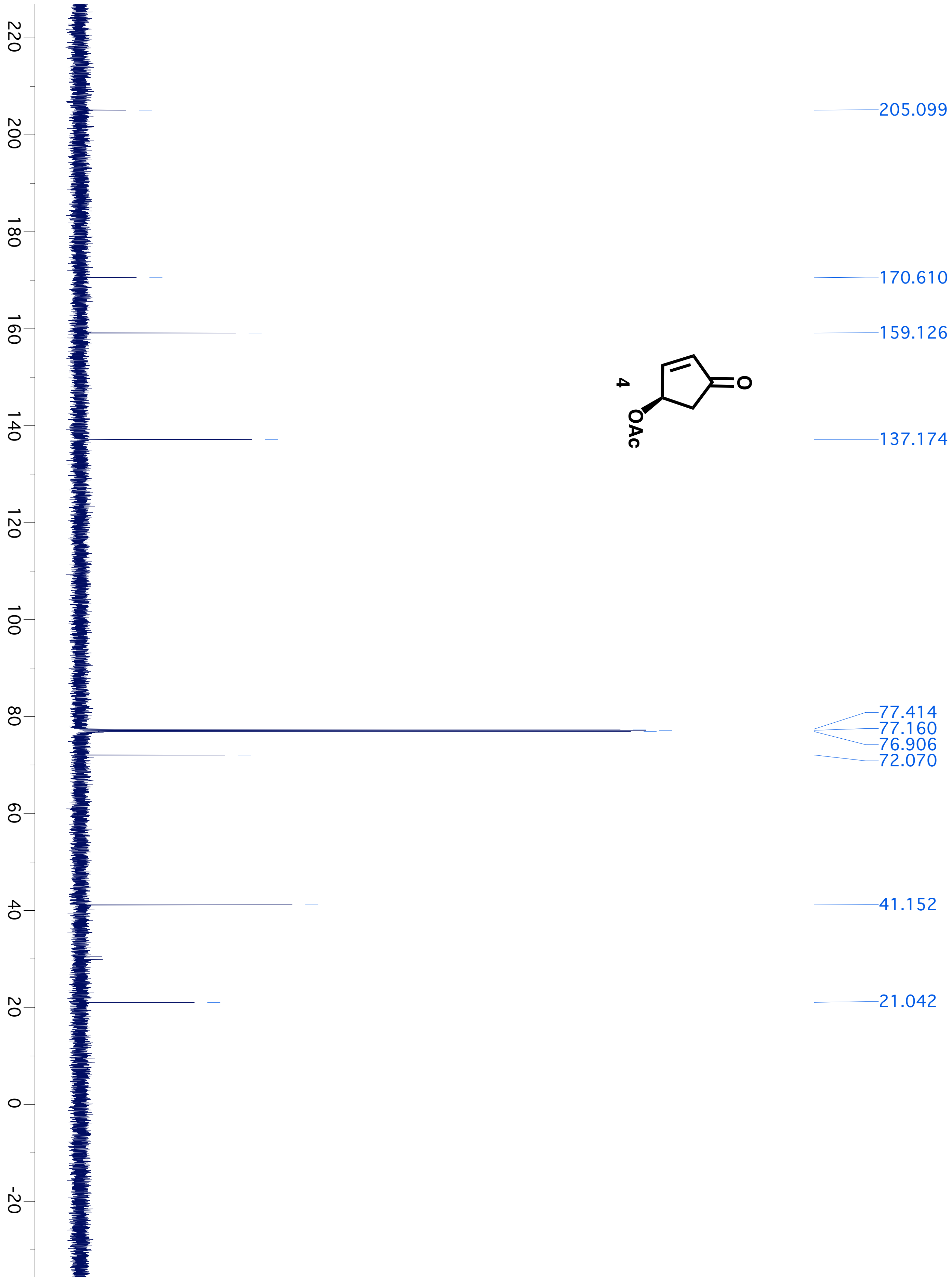
205.099
170.610
159.126
137.174
77.414
77.160
76.906
72.070
41.152
21.042
4
OAc
O

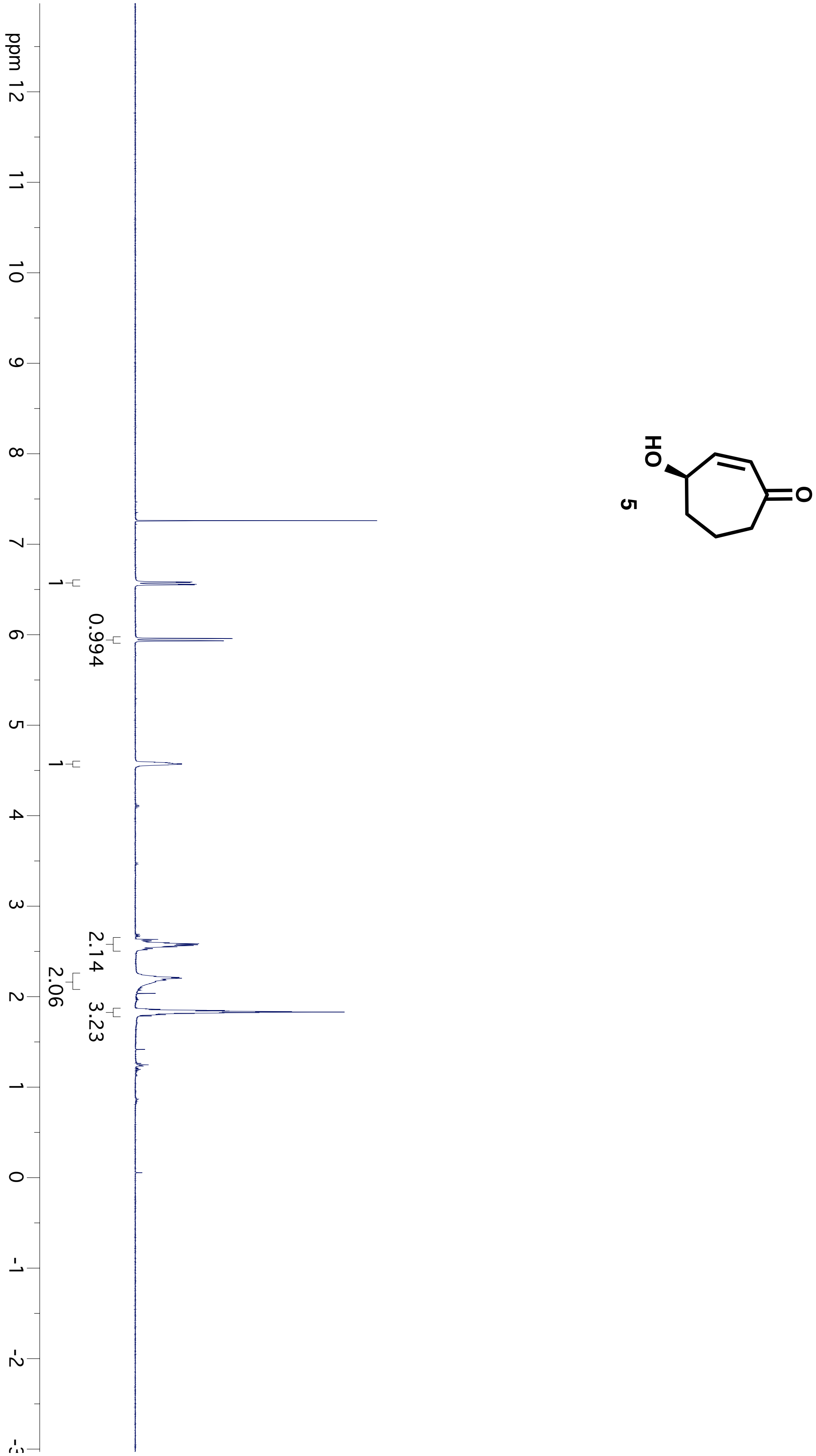
HO
O
5
ppm
12
11
10
9
8
7
6
5
4
3
2
1
0
-1
-2
1
0.994
1
2.14
2.06
3.23

Electronic Supplementary Material (ESI) for Chemical Science
This journal is © The Royal Society of Chemistry 2014

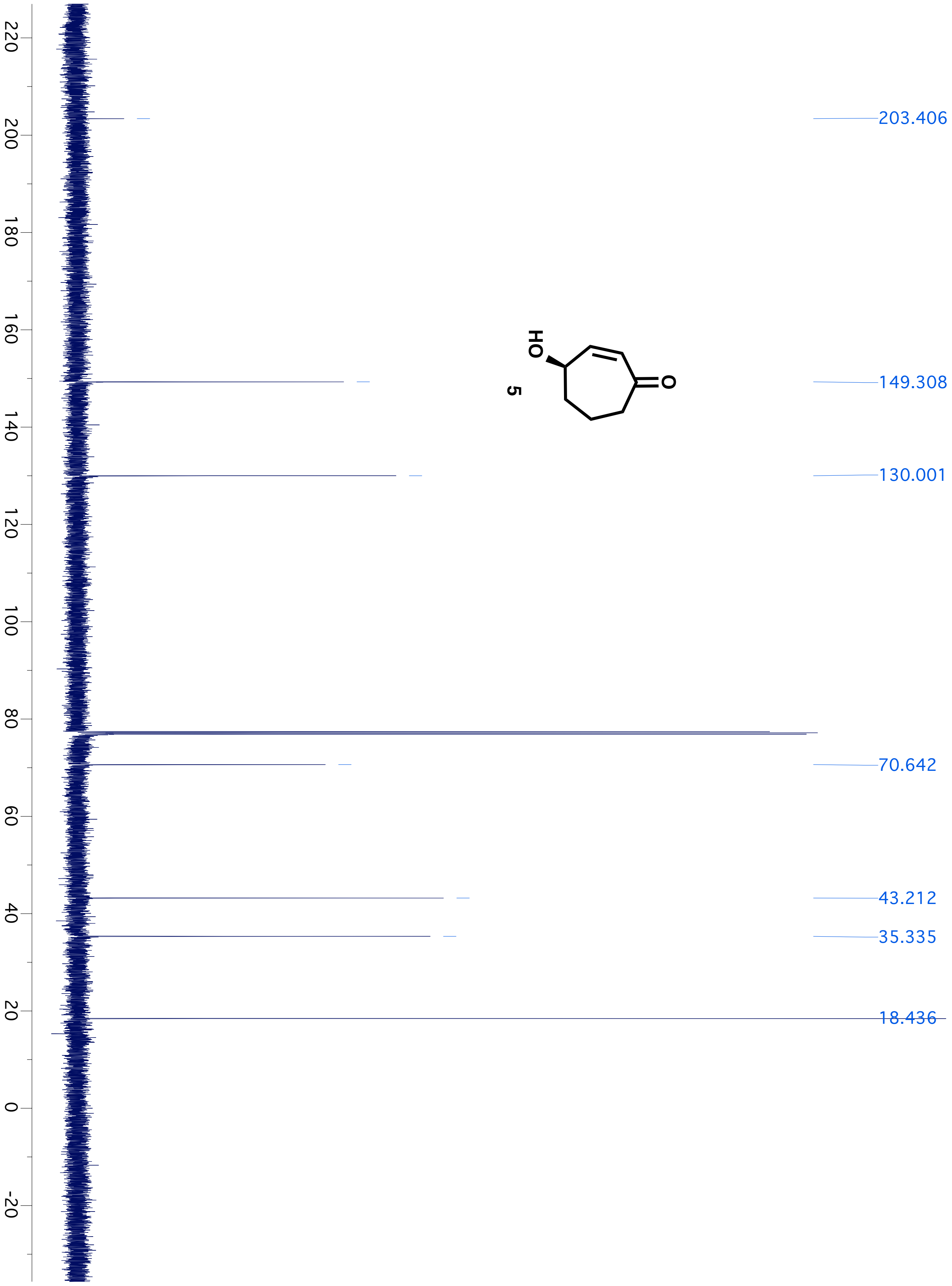

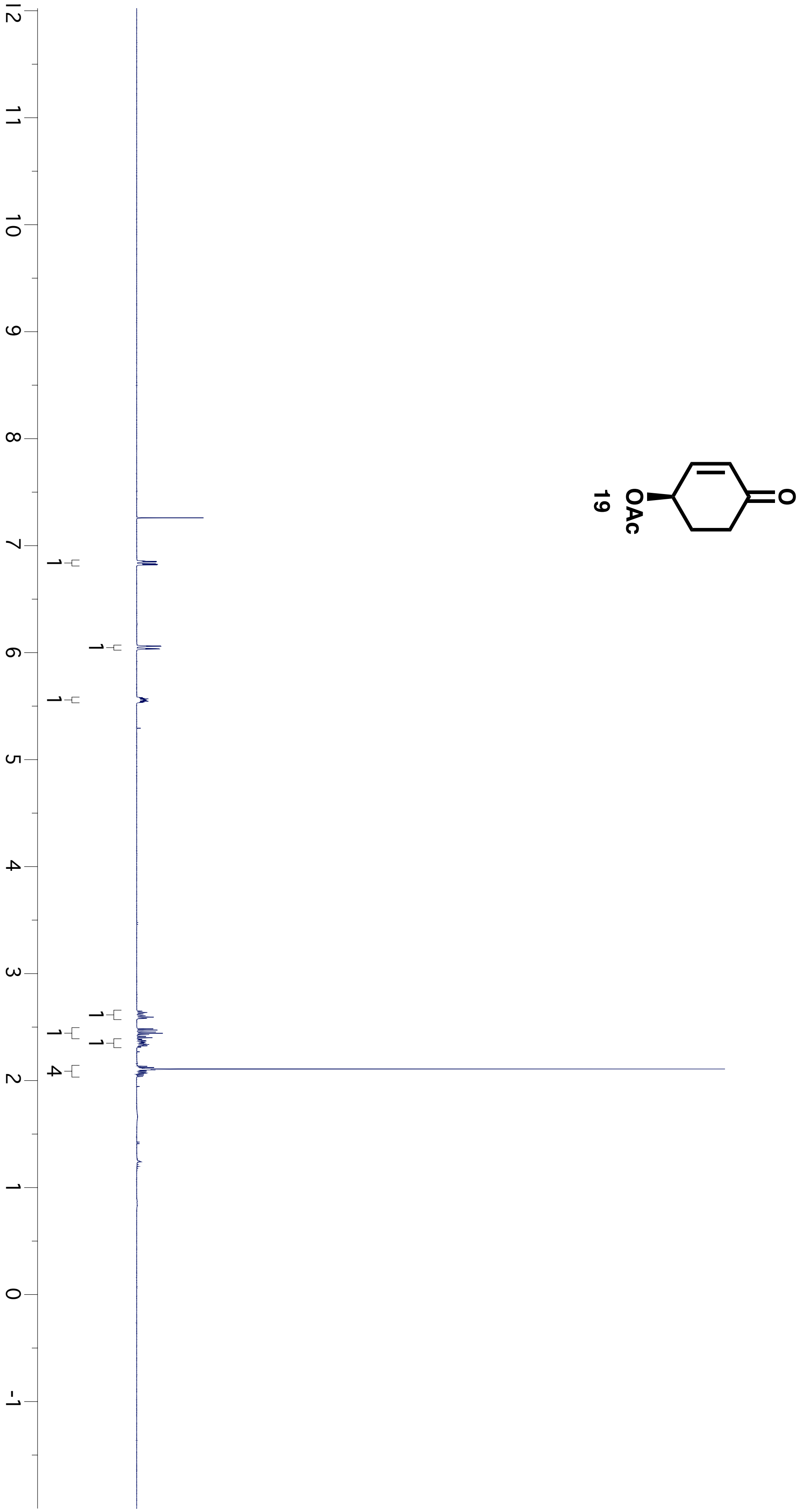

19

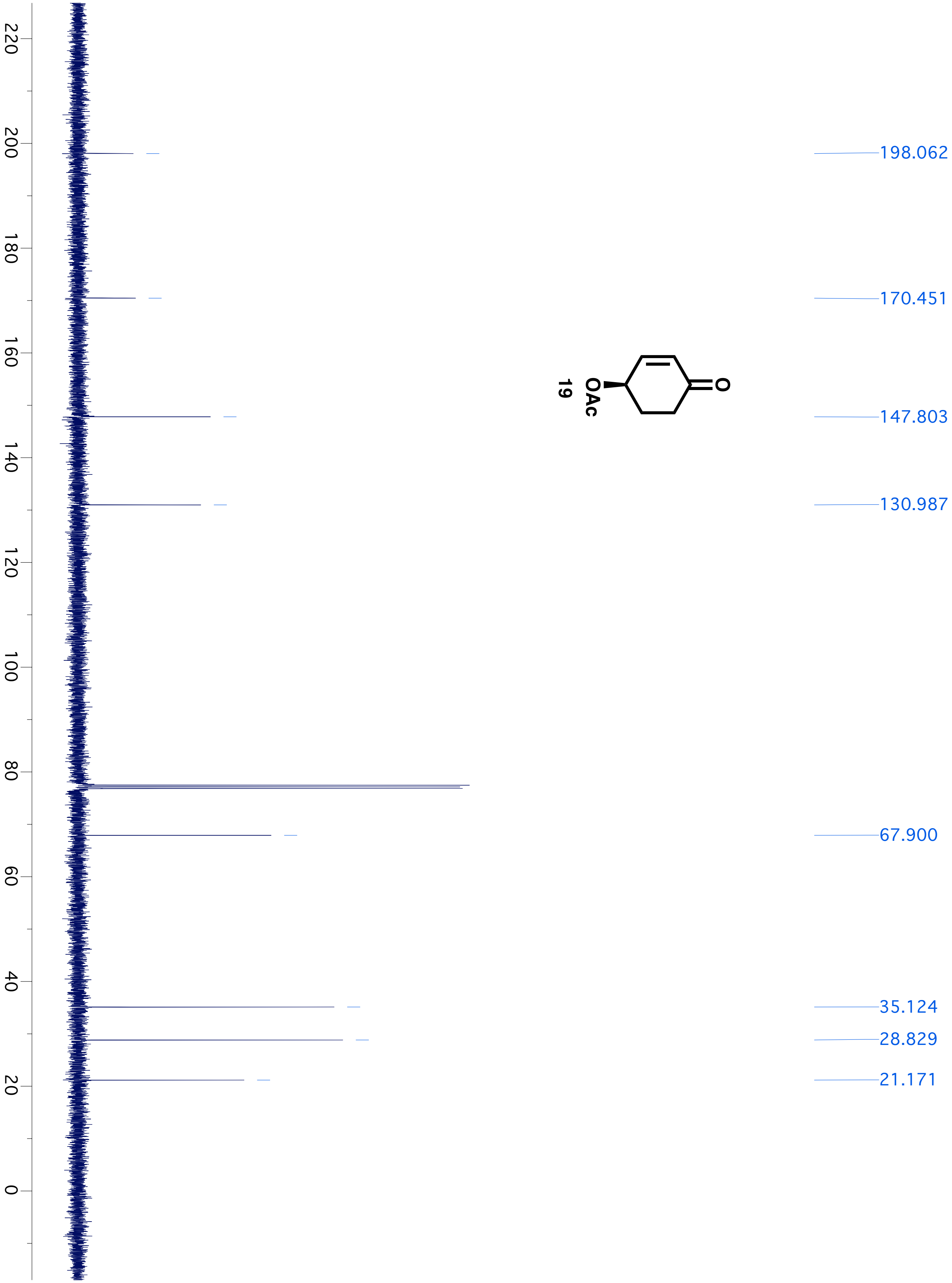
198.062
170.451
147.803
130.987
67.900
35.124
28.829
21.171
OAc
O
19
220
200
180
160
140
120
100
80
60
40
20
0

Electronic Supplementary Material (ESI) for Chemical Science
This journal is © The Royal Society of Chemistry 2014

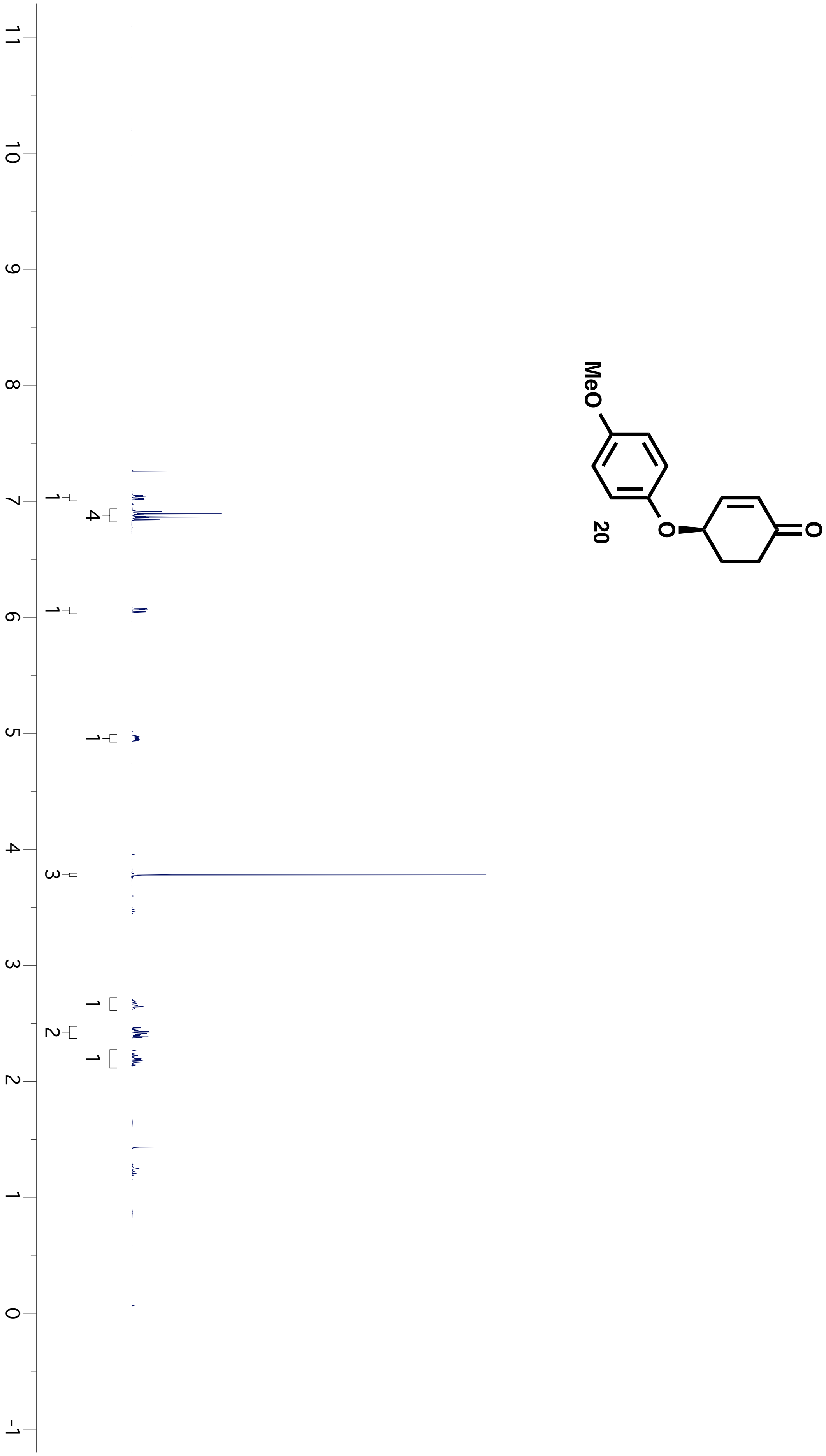

Electronic Supplementary Material (ESI) for Chemical Science
This journal is © The Royal Society of Chemistry 2014

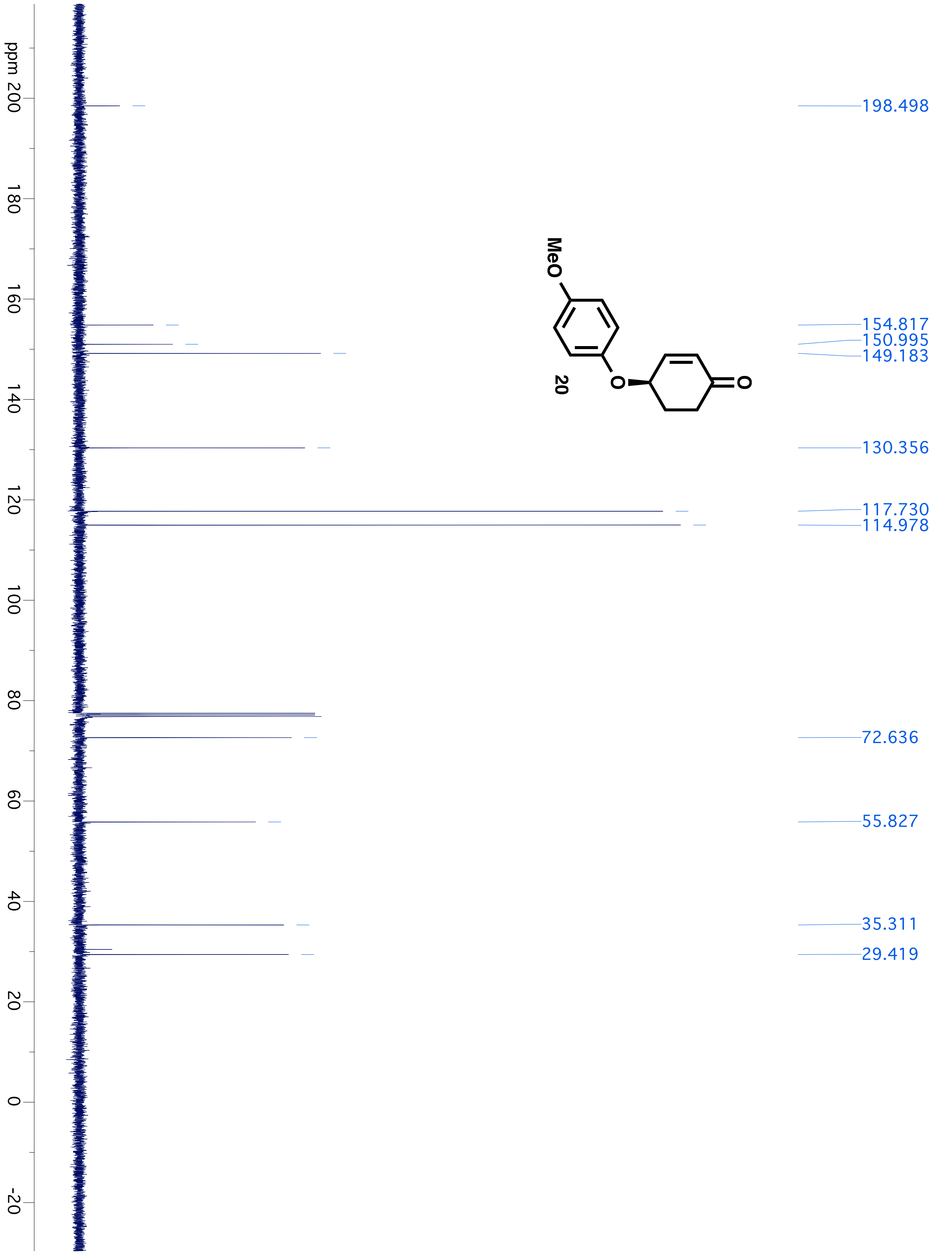

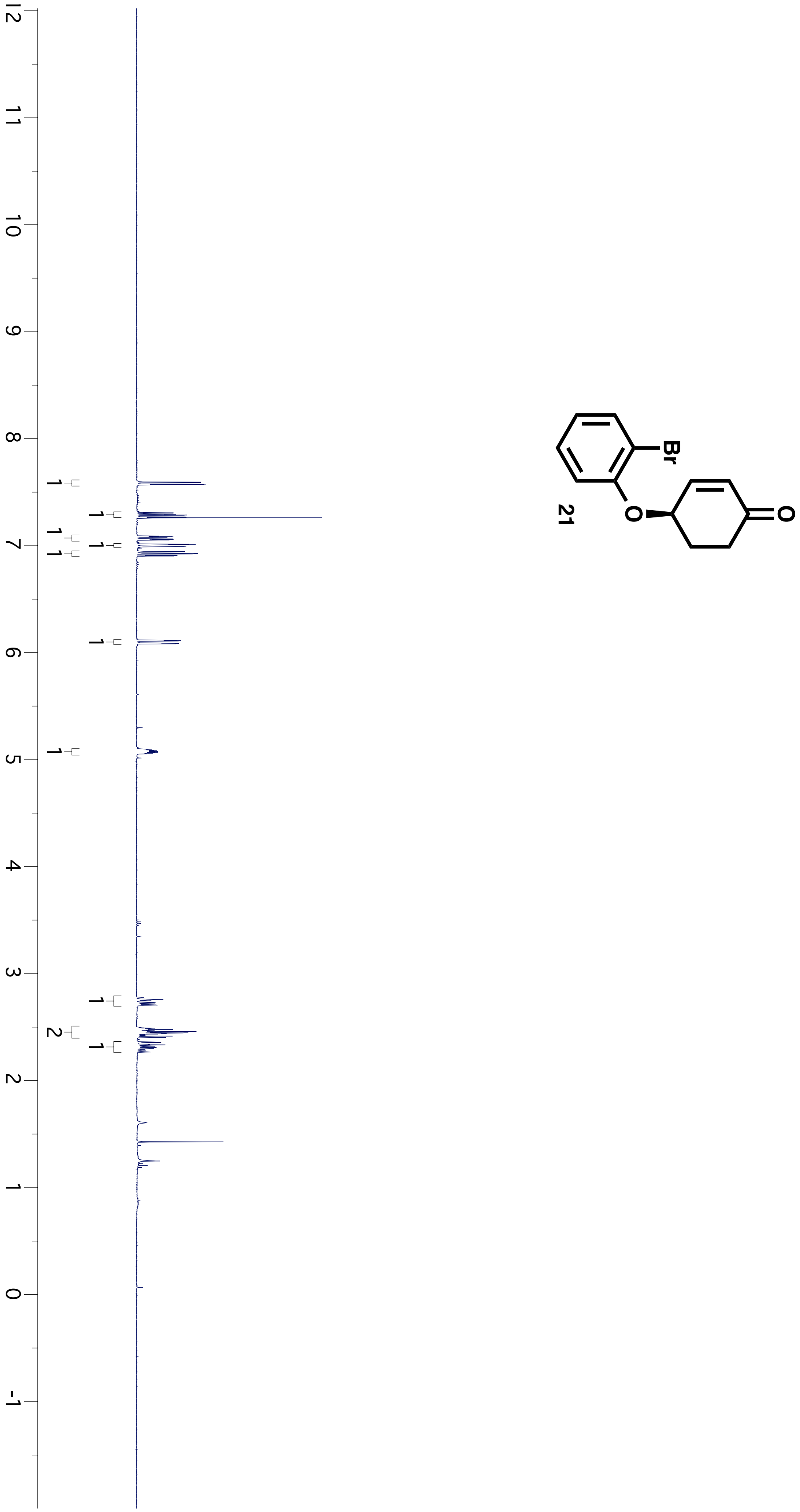
Br
O
O
21

Electronic Supplementary Material (ESI) for Chemical Science
This journal is © The Royal Society of Chemistry 2014

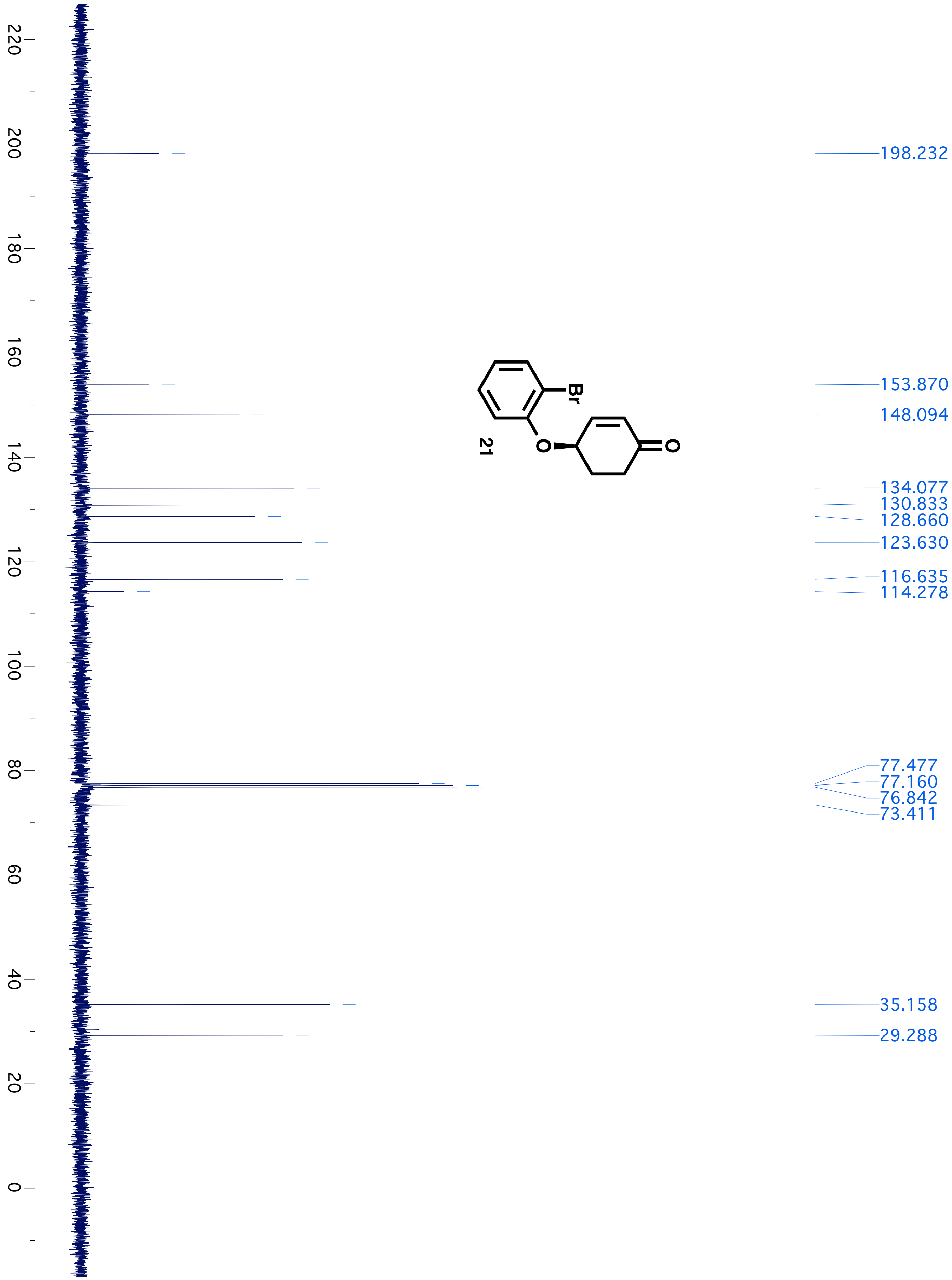

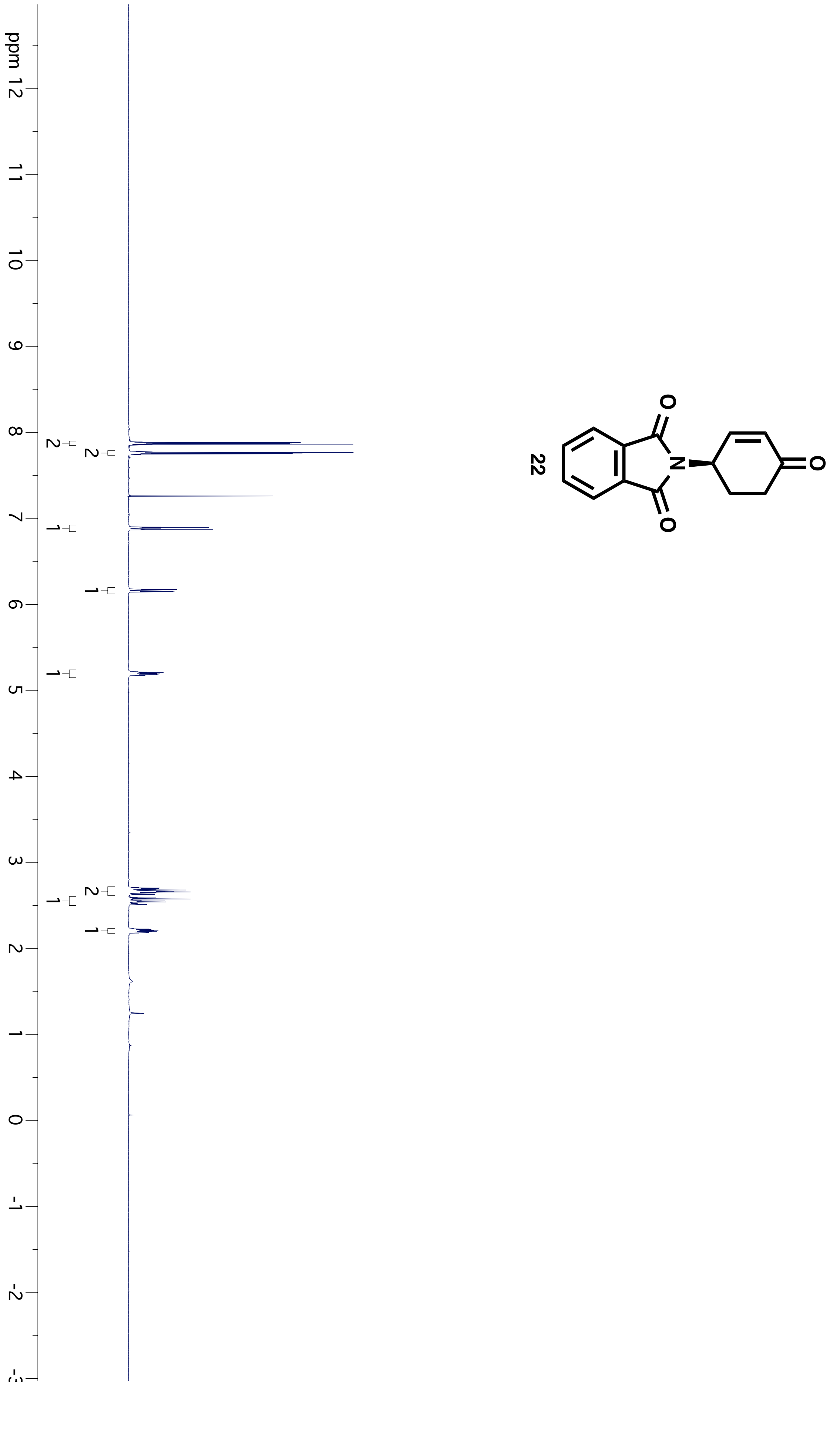
22
ppm 12
11
10
9
8
7
6
5
4
3
2
1
0
-1
-2

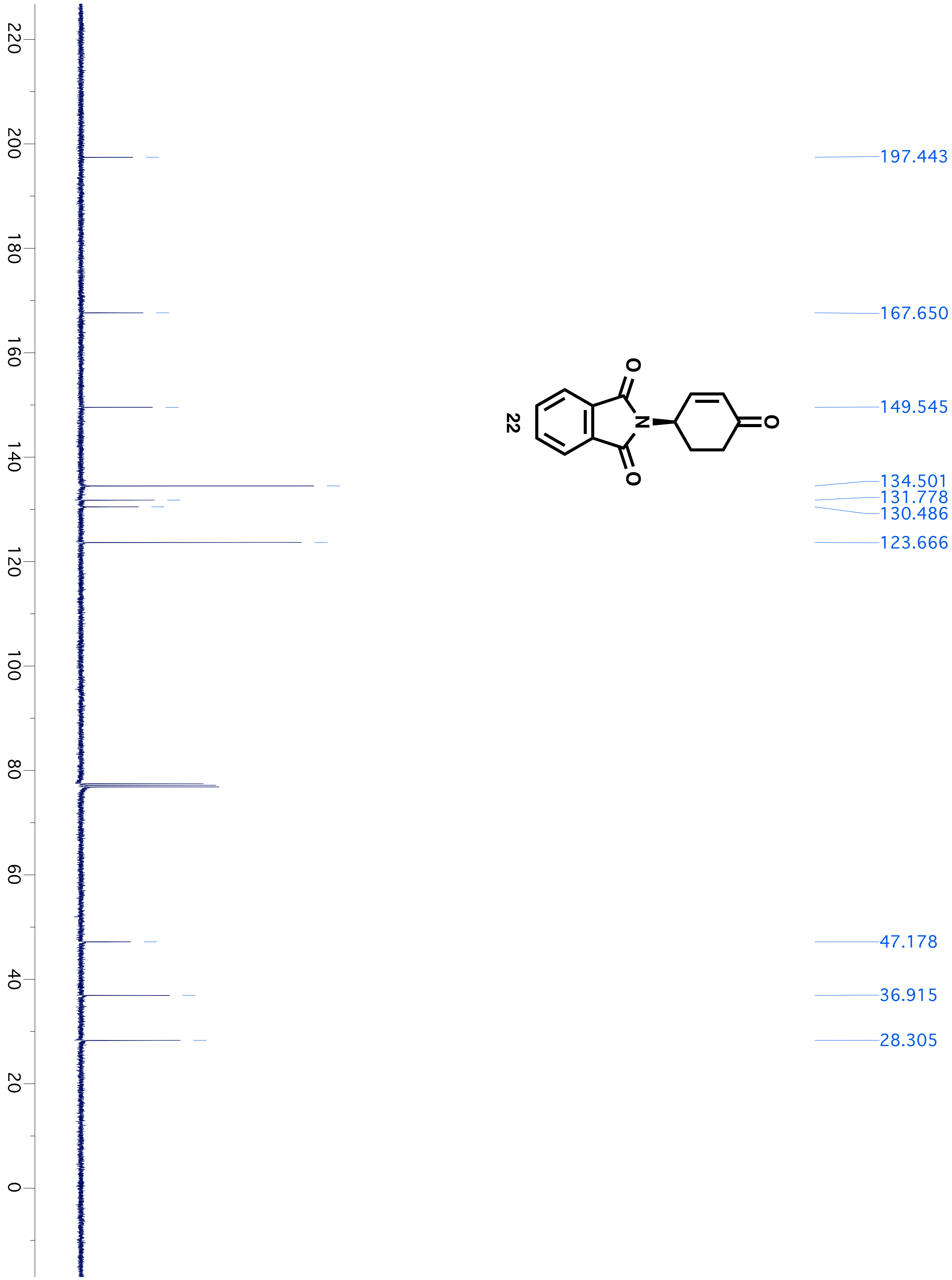
22
197.443
167.650
149.545
134.501
131.778
130.486
123.666
47.178
36.915
28.305
220
200
180
160
140
120
100
80
60
40
20
0

Electronic Supplementary Material (ESI) for Chemical Science
This journal is © The Royal Society of Chemistry 2014

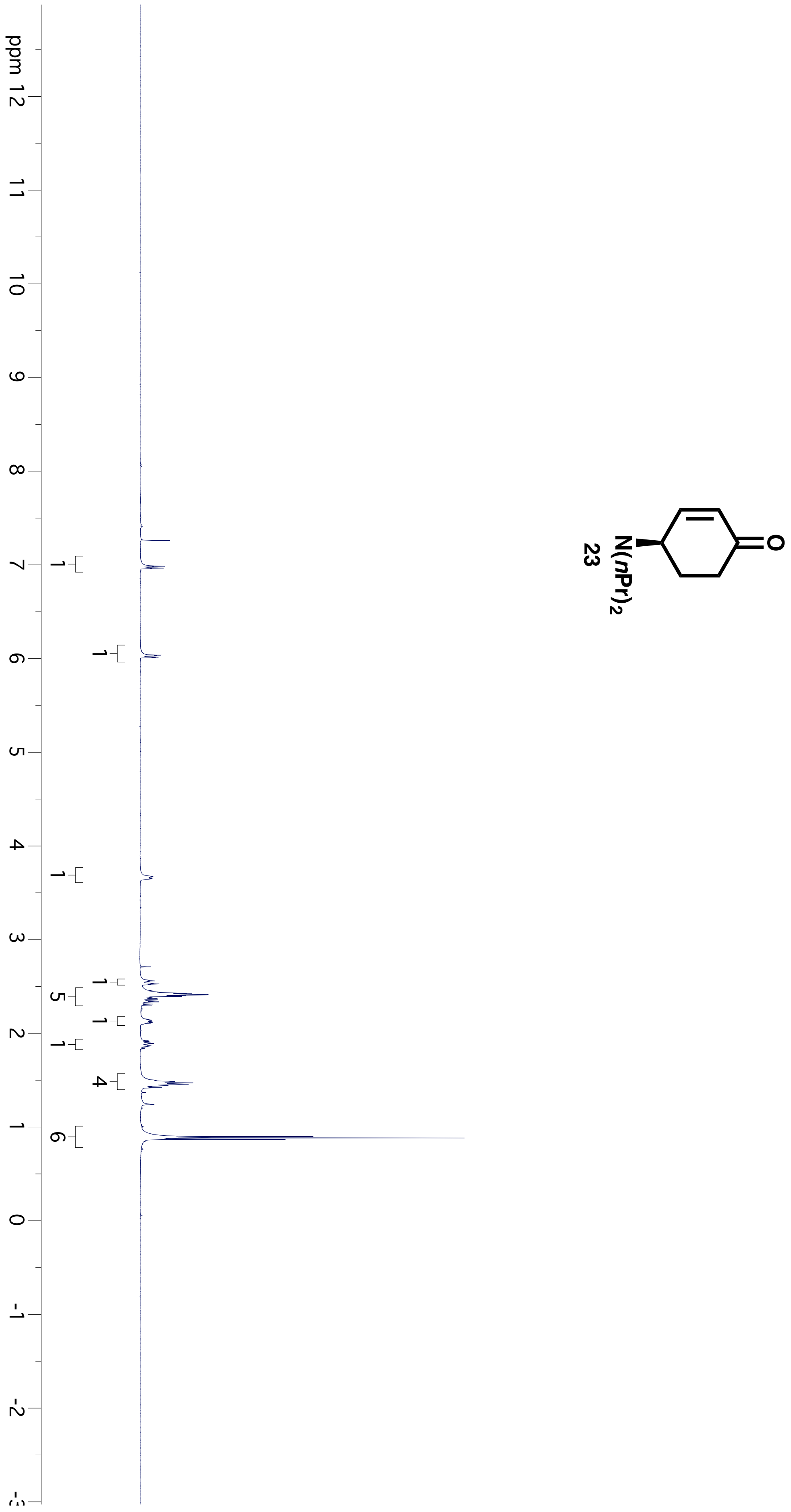

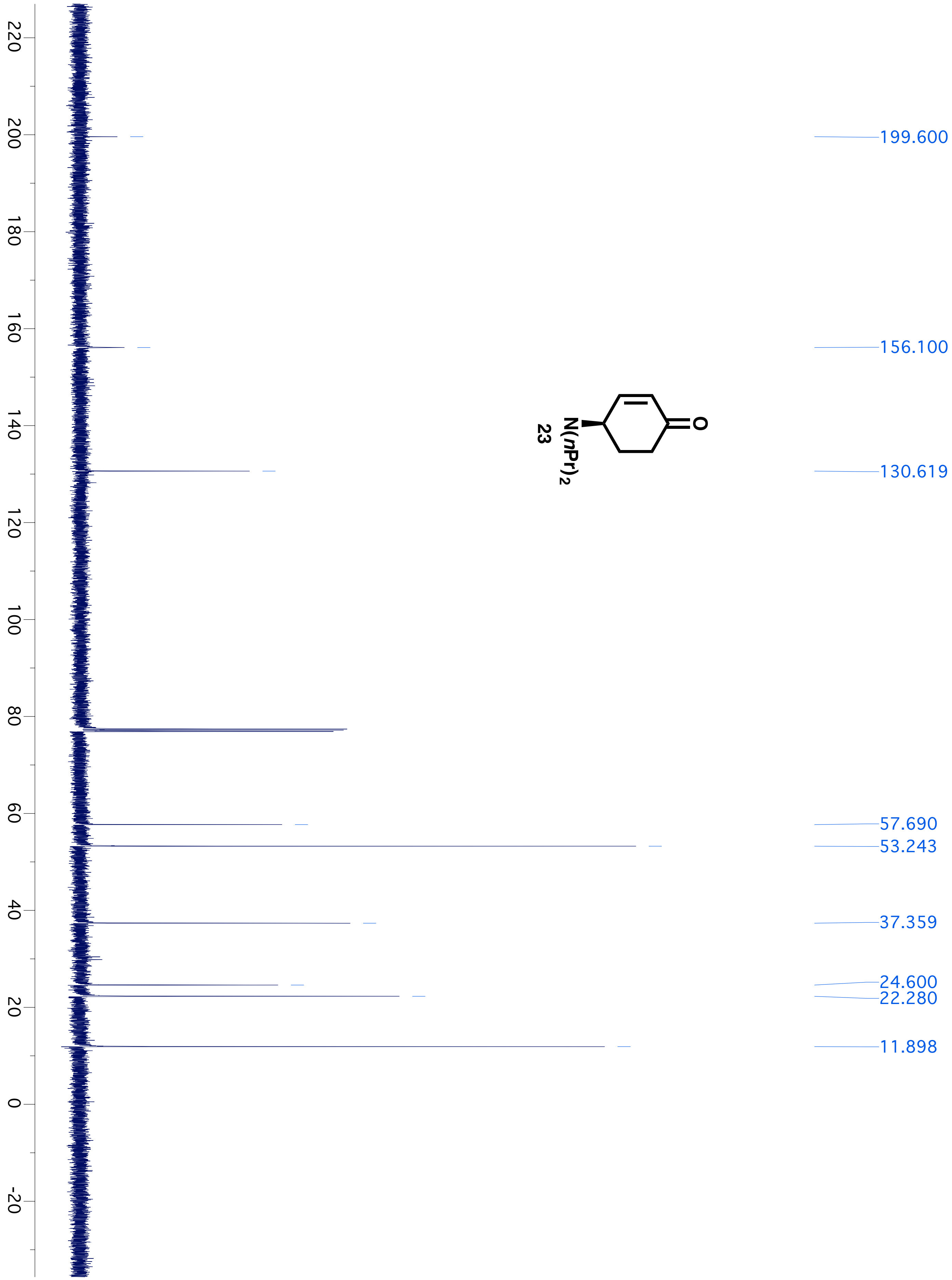
199.600
156.100
130.619
57.690
53.243
37.359
24.600
22.280
11.898
N(nPr)2
23

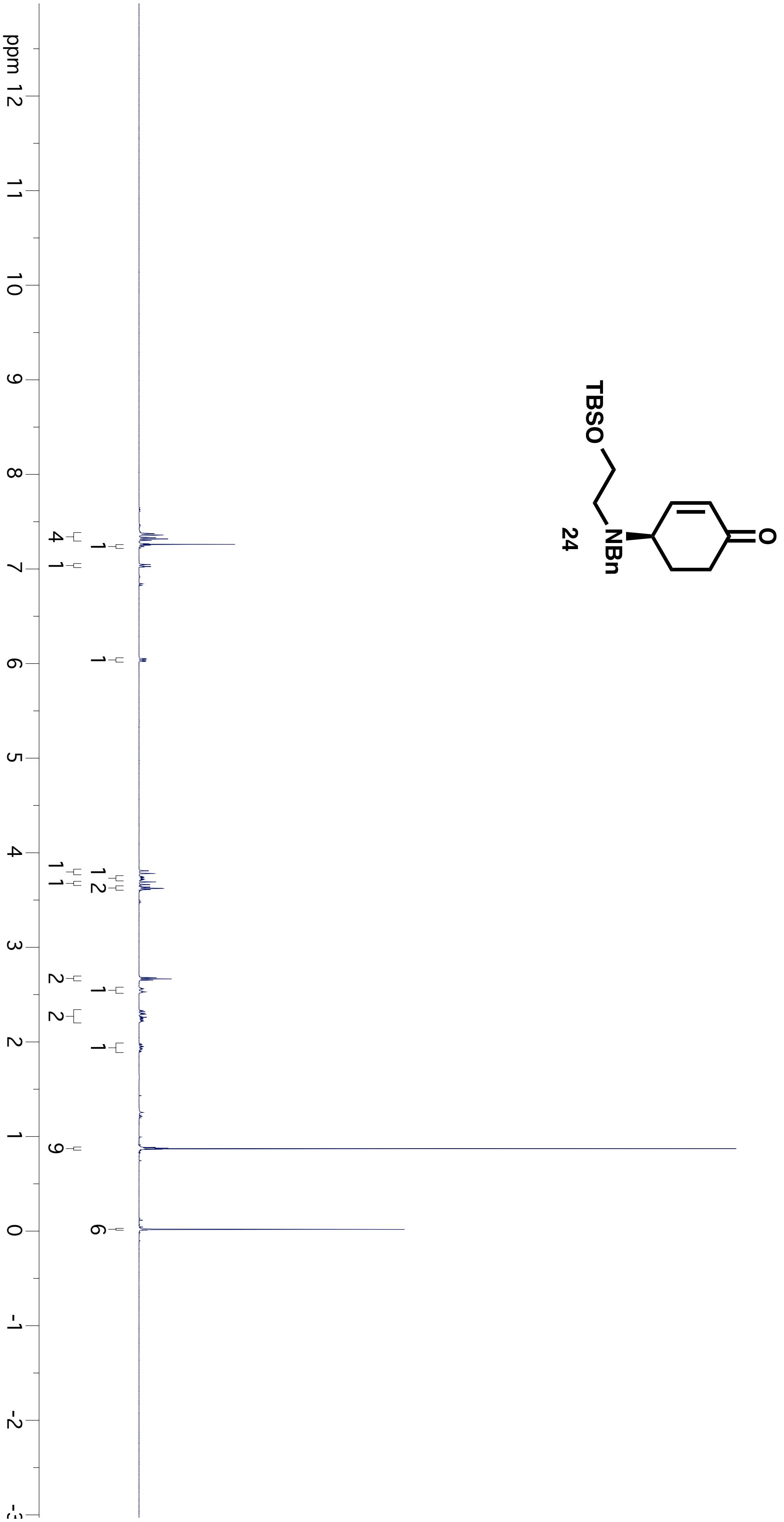
TBSO
NBn
O
24
ppm

Electronic Supplementary Material (ESI) for Chemical Science
This journal is © The Royal Society of Chemistry 2014

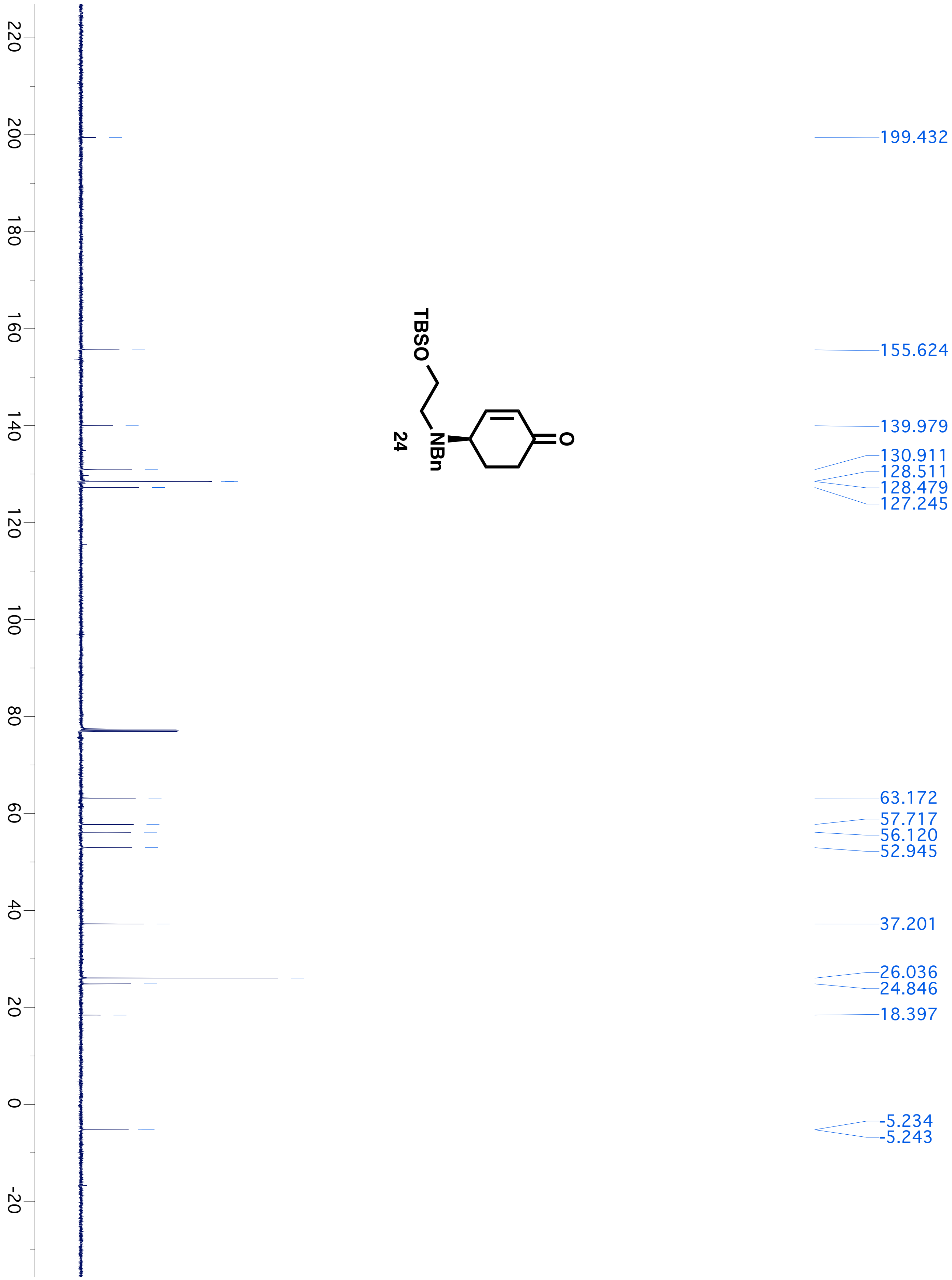

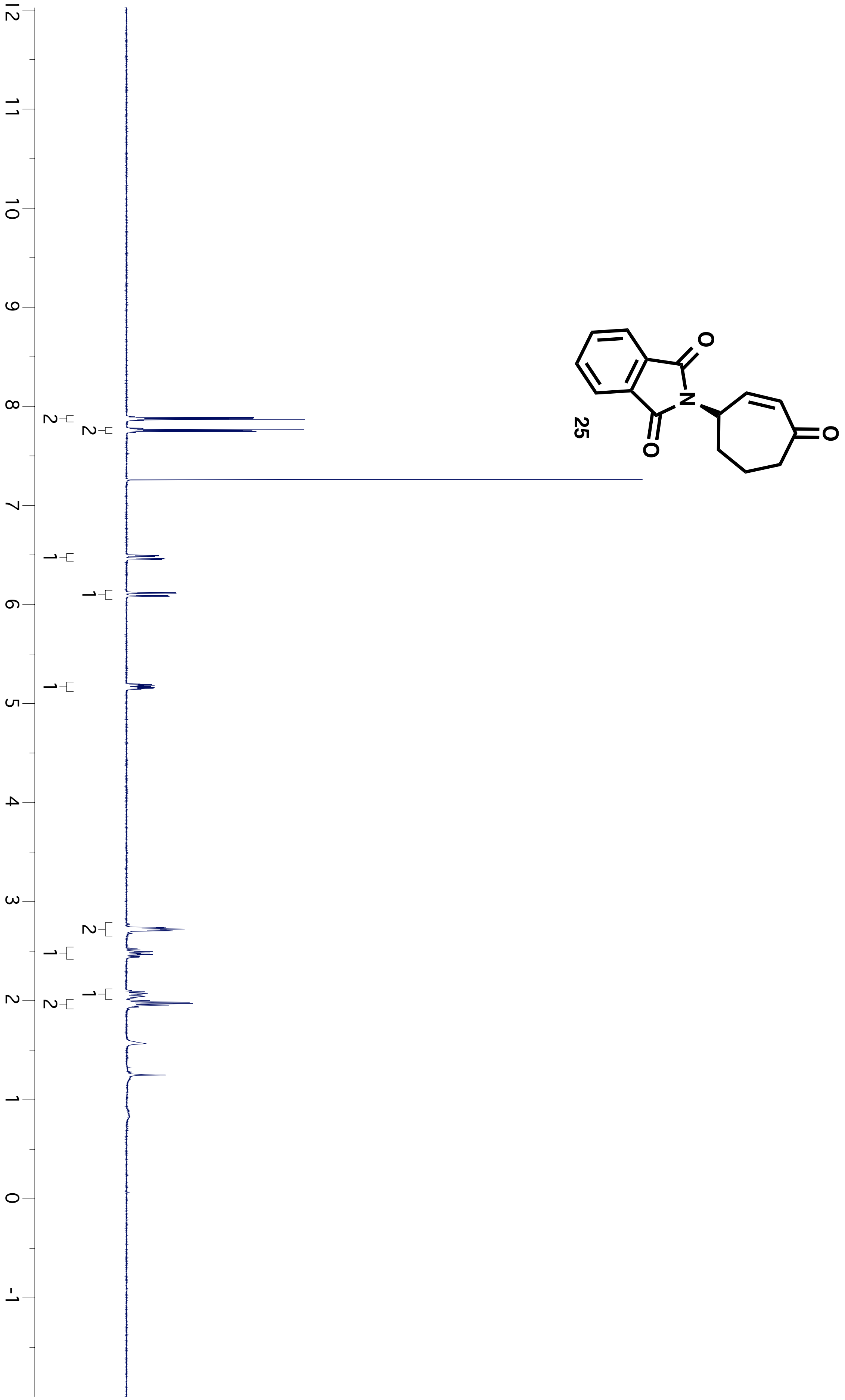
25

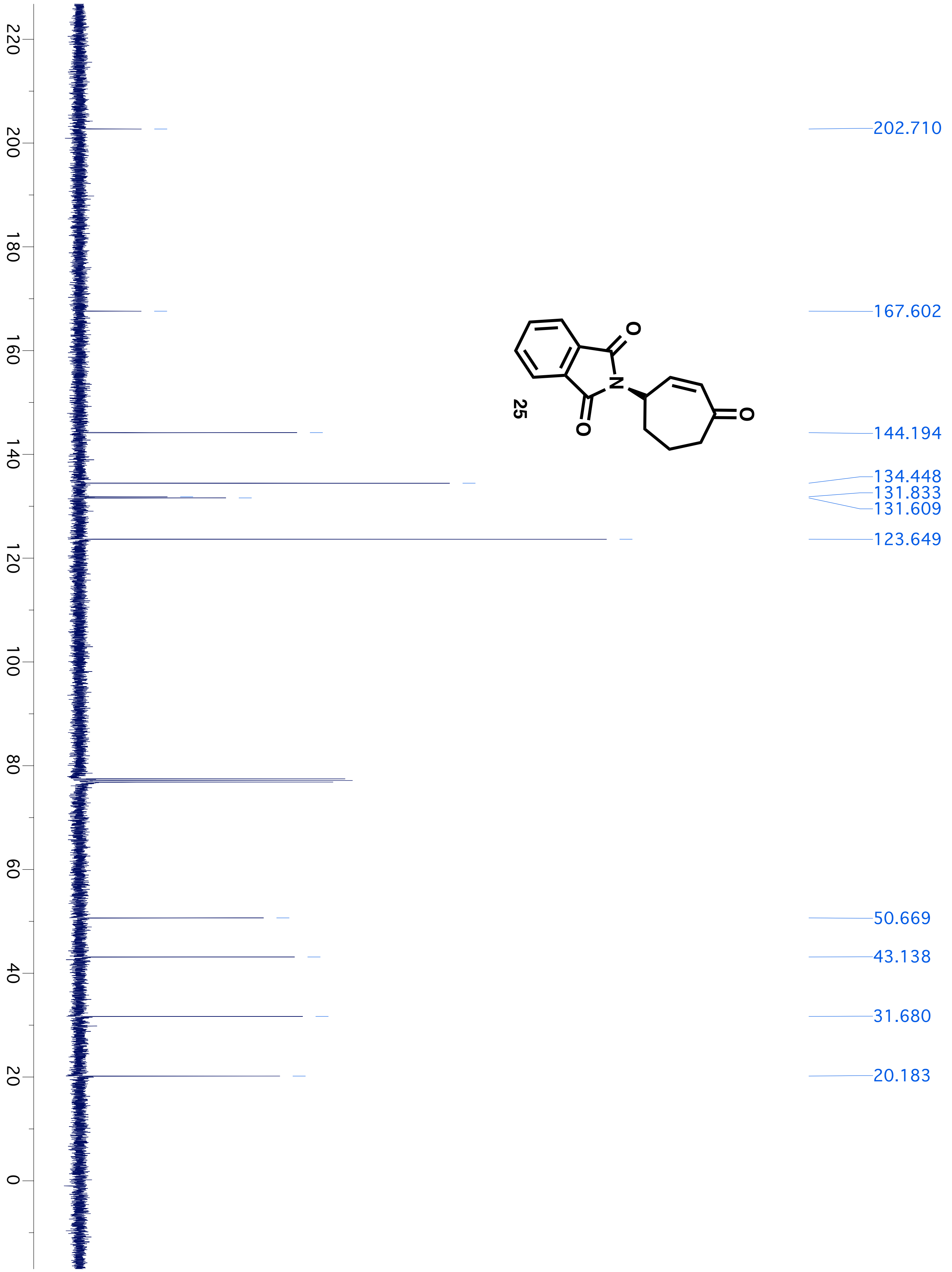
202.710
167.602
144.194
134.448
131.833
131.609
123.649
50.669
43.138
31.680
20.183
25
220
200
180
160
140
120
100
80
60
40
20
0

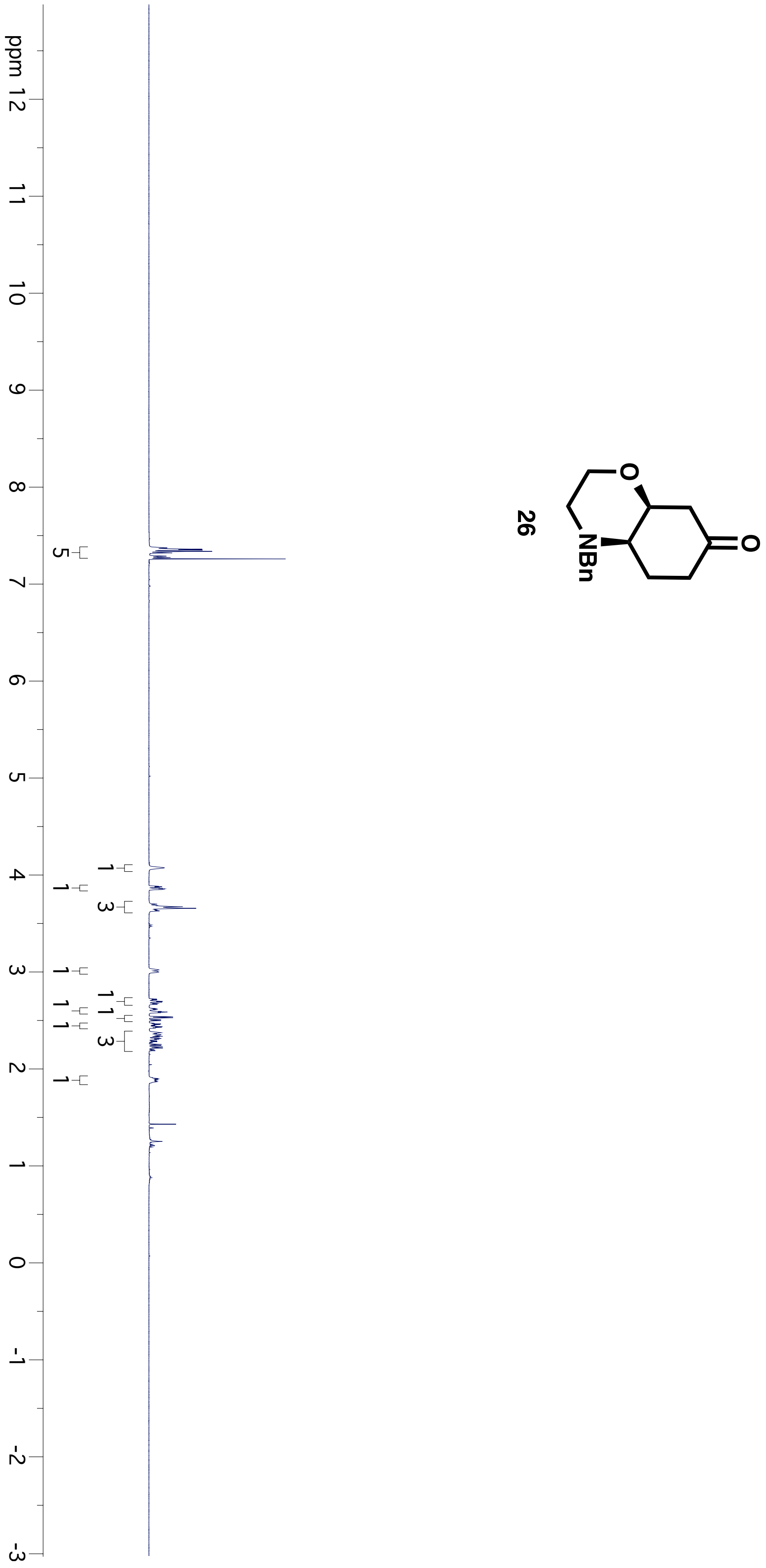

26

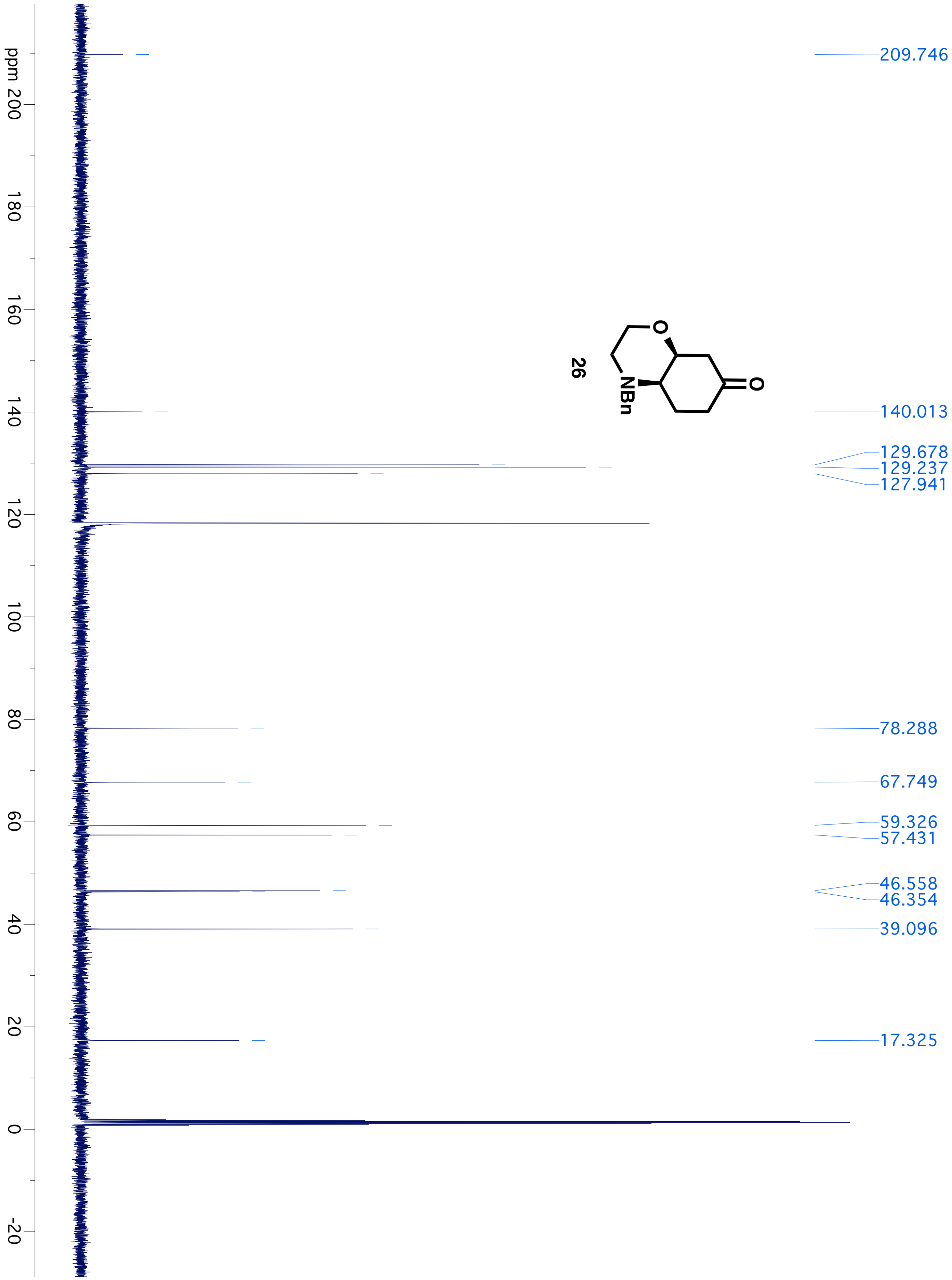
209.746
140.013
129.678
129.237
127.941
78.288
67.749
59.326
57.431
46.558
46.354
39.096
17.325
26
NBn
O
O
ppm 200
180
160
140
120
100
80
60
40
20
0
-20

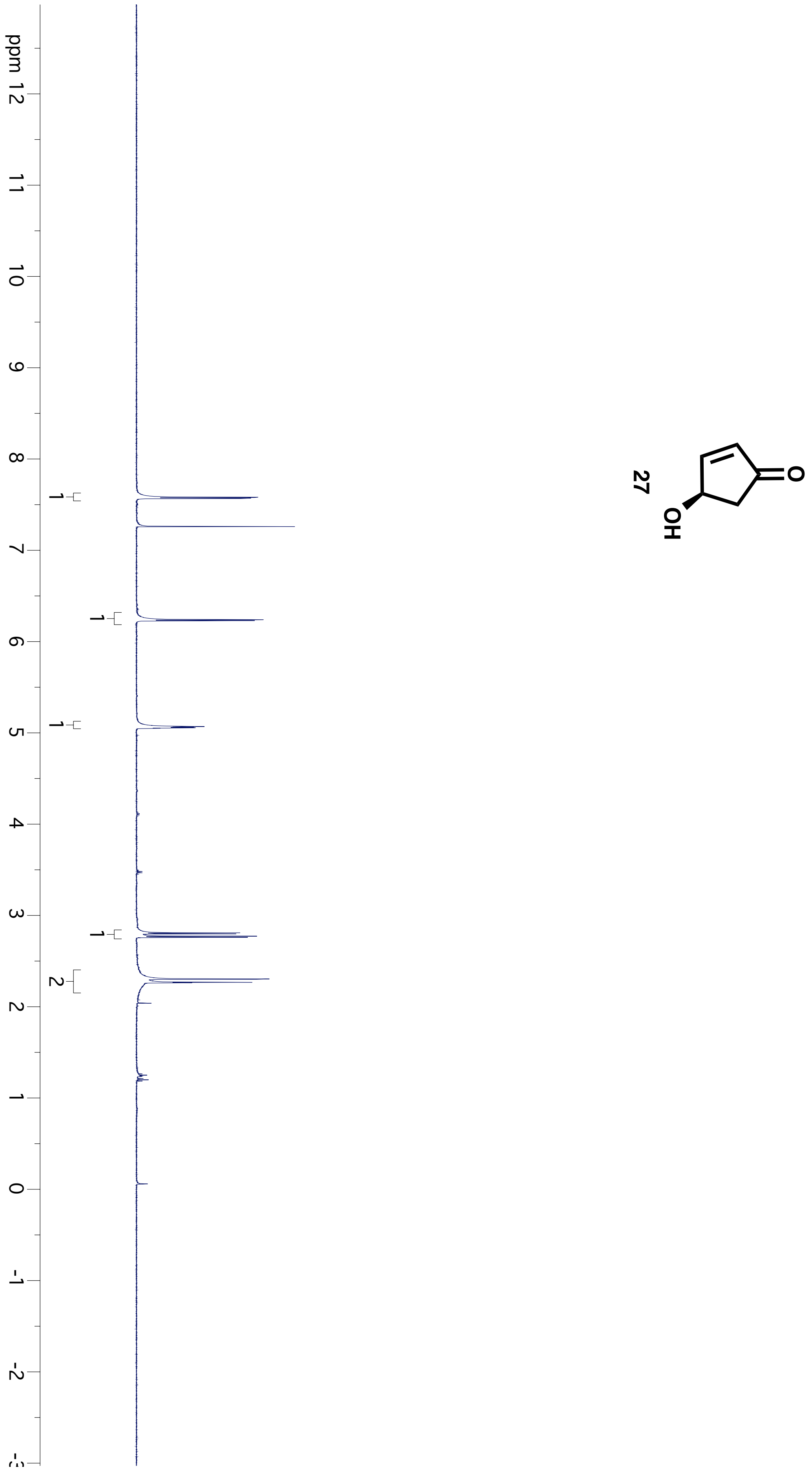
27
OH
O
ppm 12
11
10
9
8
7
6
5
4
3
2
1
0
-1
-2
1
1
1
1
2

Electronic Supplementary Material (ESI) for Chemical Science
This journal is © The Royal Society of Chemistry 2014

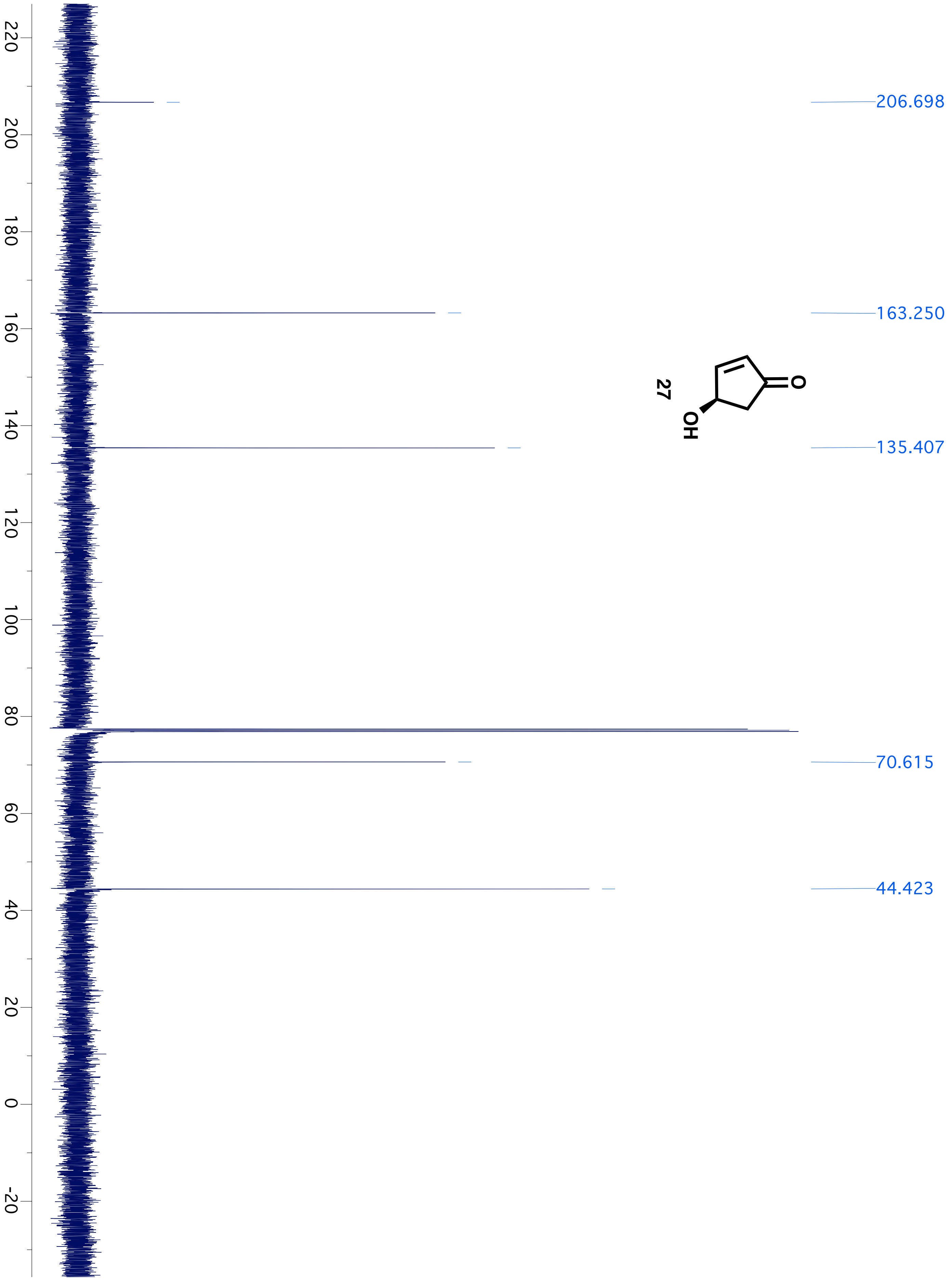

This journal is © The Royal Society of Chemistry 2014

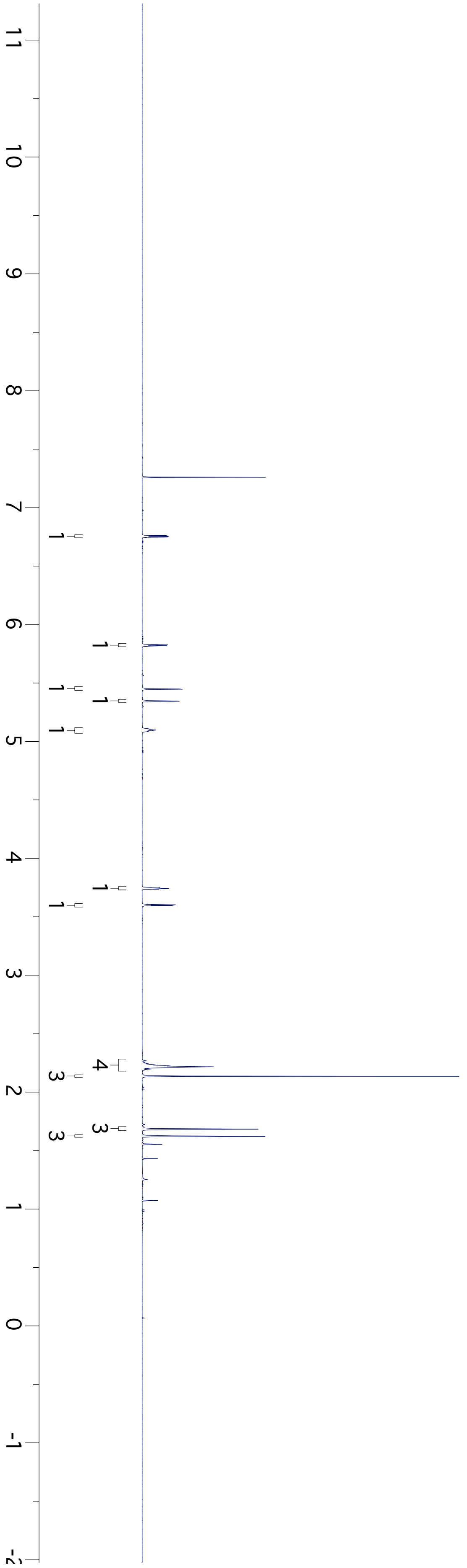

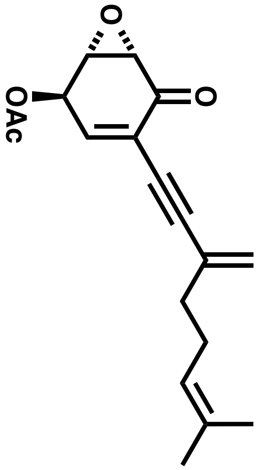

(–)-Tricholomenyn A (28)

Electronic Supplementary Material (ESI) for Chemical Science
This journal is © The Royal Society of Chemistry 2014

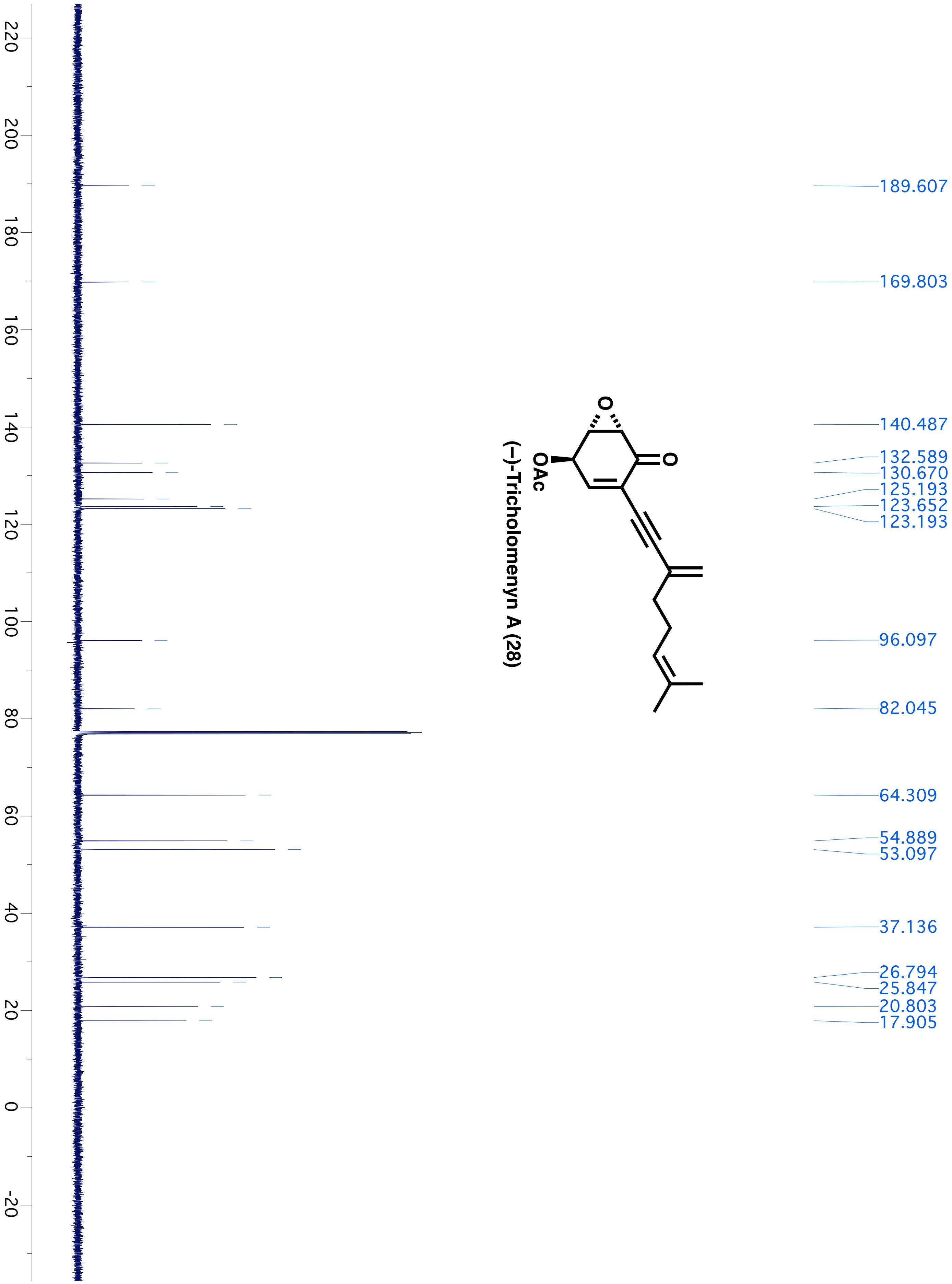

Electronic Supplementary Material (ESI) for Chemical Science
This journal is © The Royal Society of Chemistry 2014

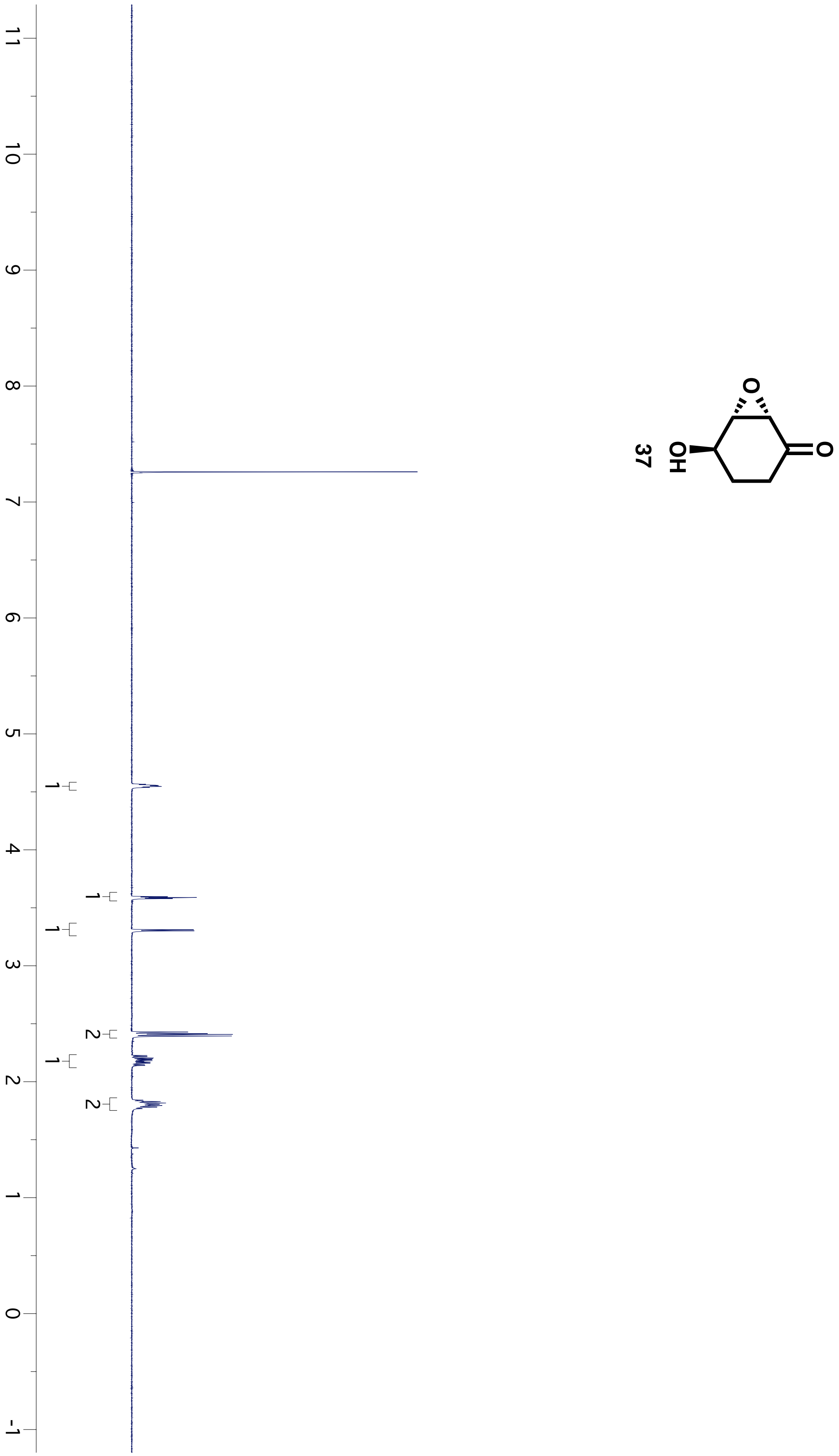

37

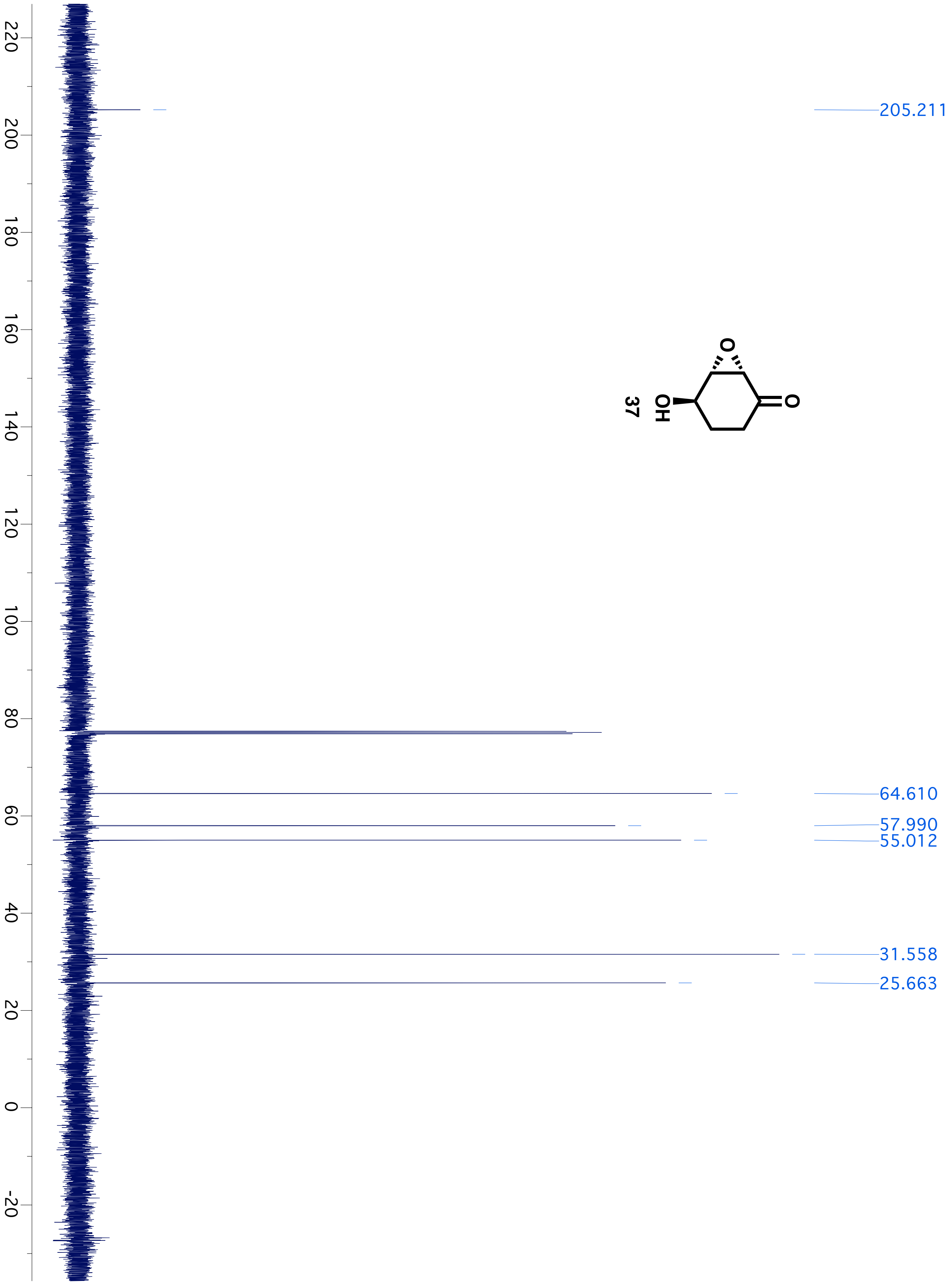
205.211
64.610
57.990
55.012
31.558
25.663
OH
37

Electronic Supplementary Material (ESI) for Chemical Science
This journal is © The Royal Society of Chemistry 2014

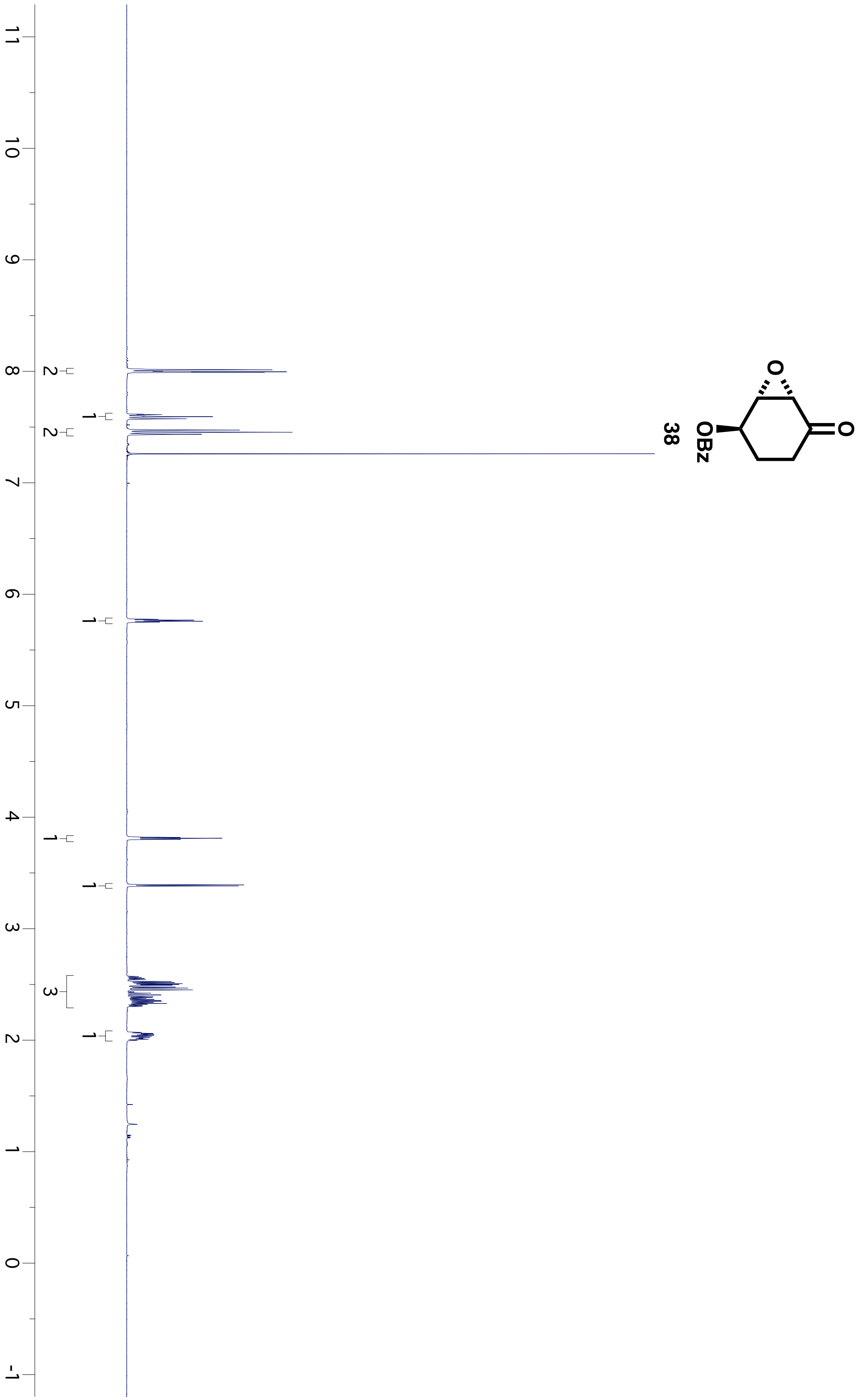

Electronic Supplementary Material (ESI) for Chemical Science
This journal is © The Royal Society of Chemistry 2014

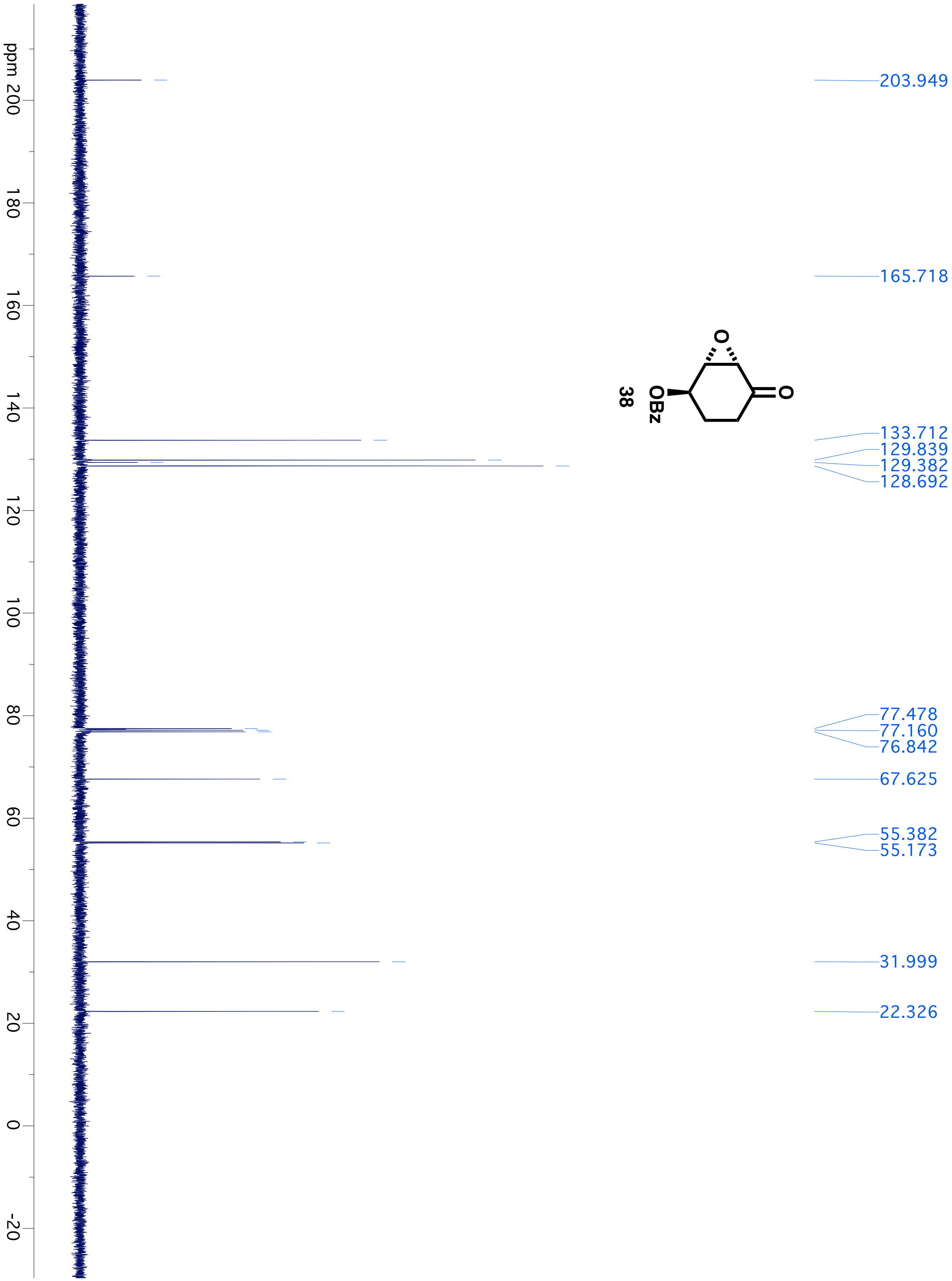

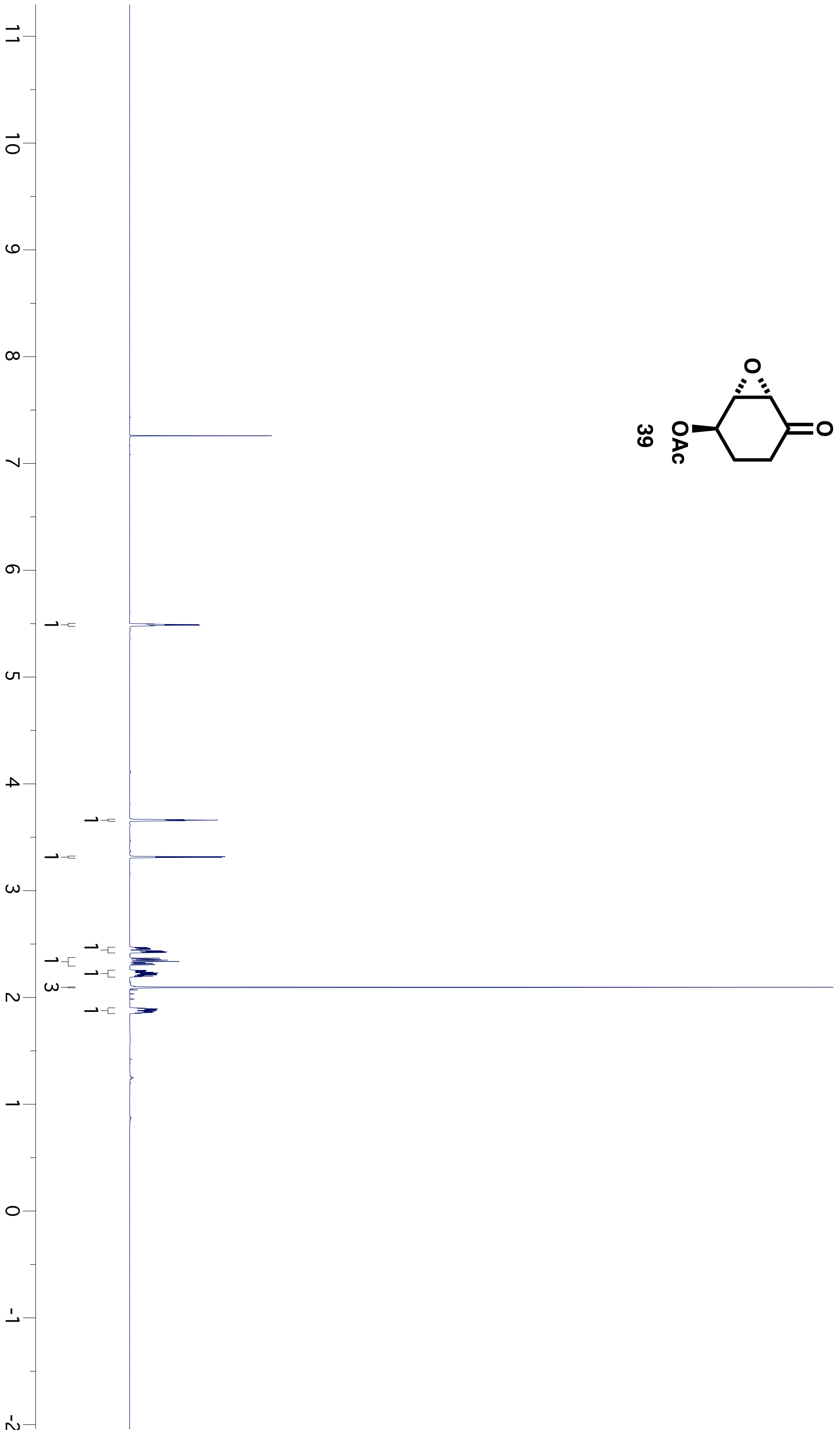
OAc
39
11
10
9
8
7
6
5
4
3
2
1
0
-1
-2

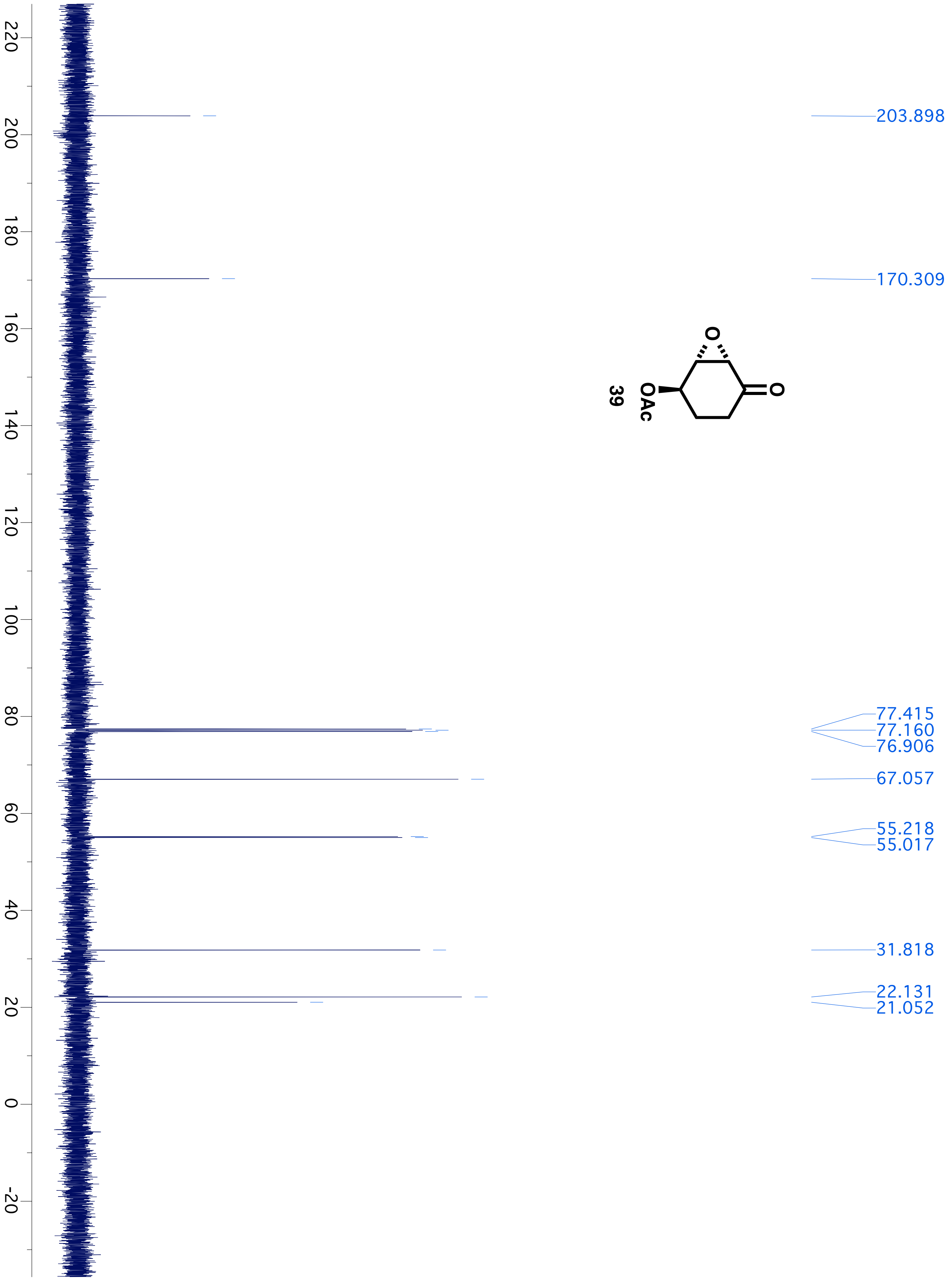
203.898
170.309
77.415
77.160
76.906
67.057
55.218
55.017
31.818
22.131
21.052
O
O
OAc
39
220
200
180
160
140
120
100
80
60
40
20
0
-20

Electronic Supplementary Material (ESI) for Chemical Science
This journal is © The Royal Society of Chemistry 2014

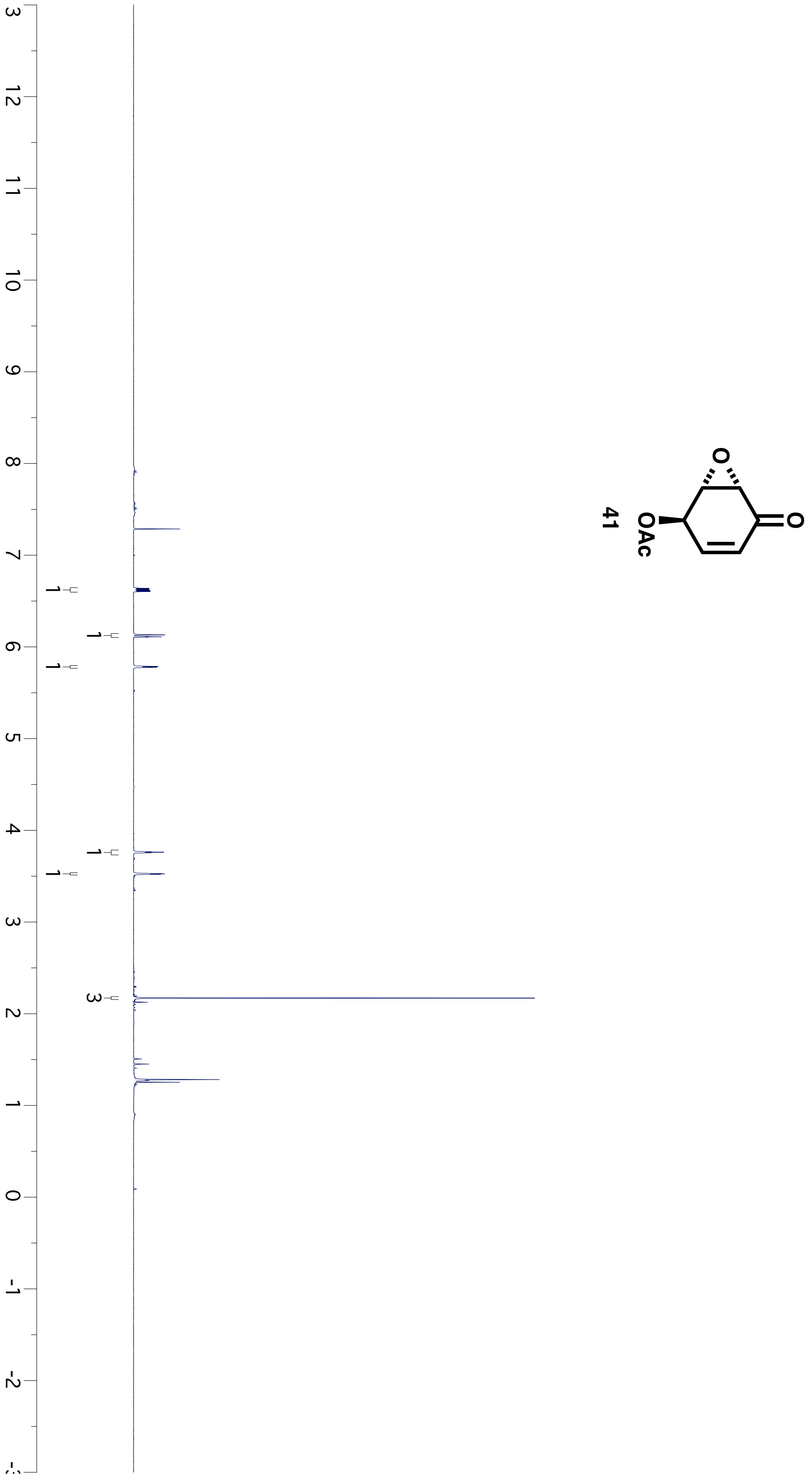

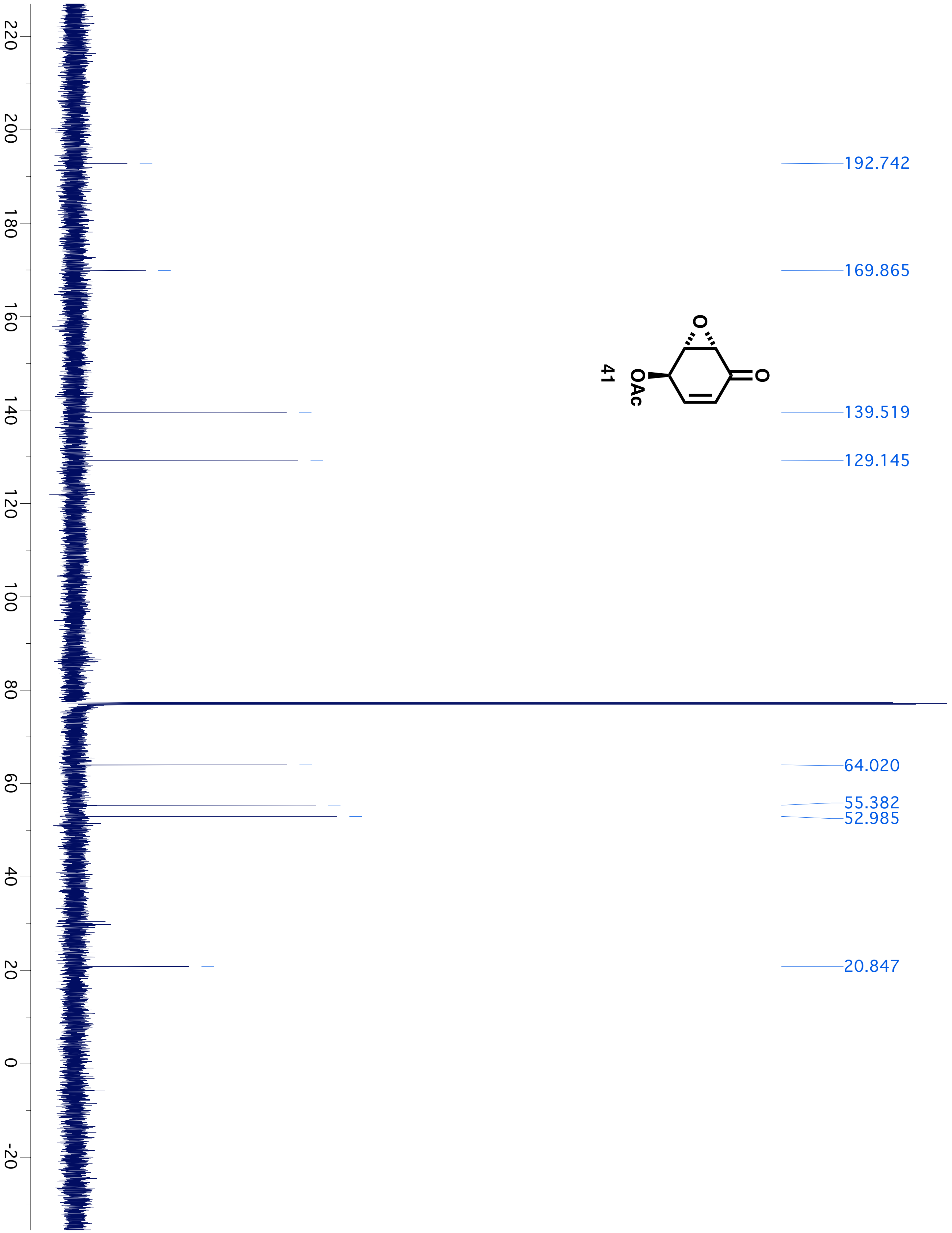
192.742
169.865
139.519
129.145
64.020
55.382
52.985
20.847
OAc
41

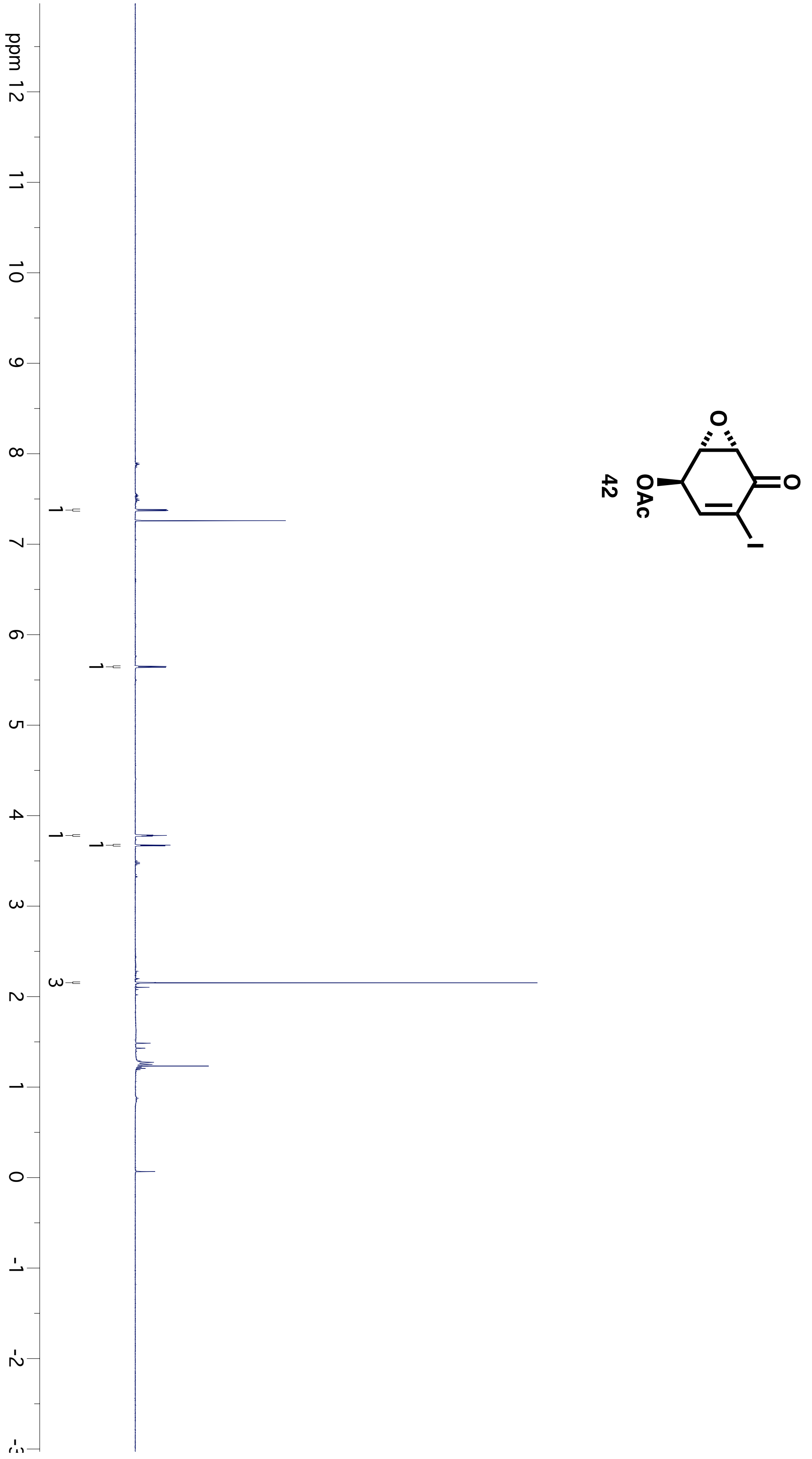
OAc
I
O
O
42
ppm
12
11
10
9
8
7
6
5
4
3
2
1
0
-1
-2
1
1
1
1
3

Electronic Supplementary Material (ESI) for Chemical Science
This journal is © The Royal Society of Chemistry 2014

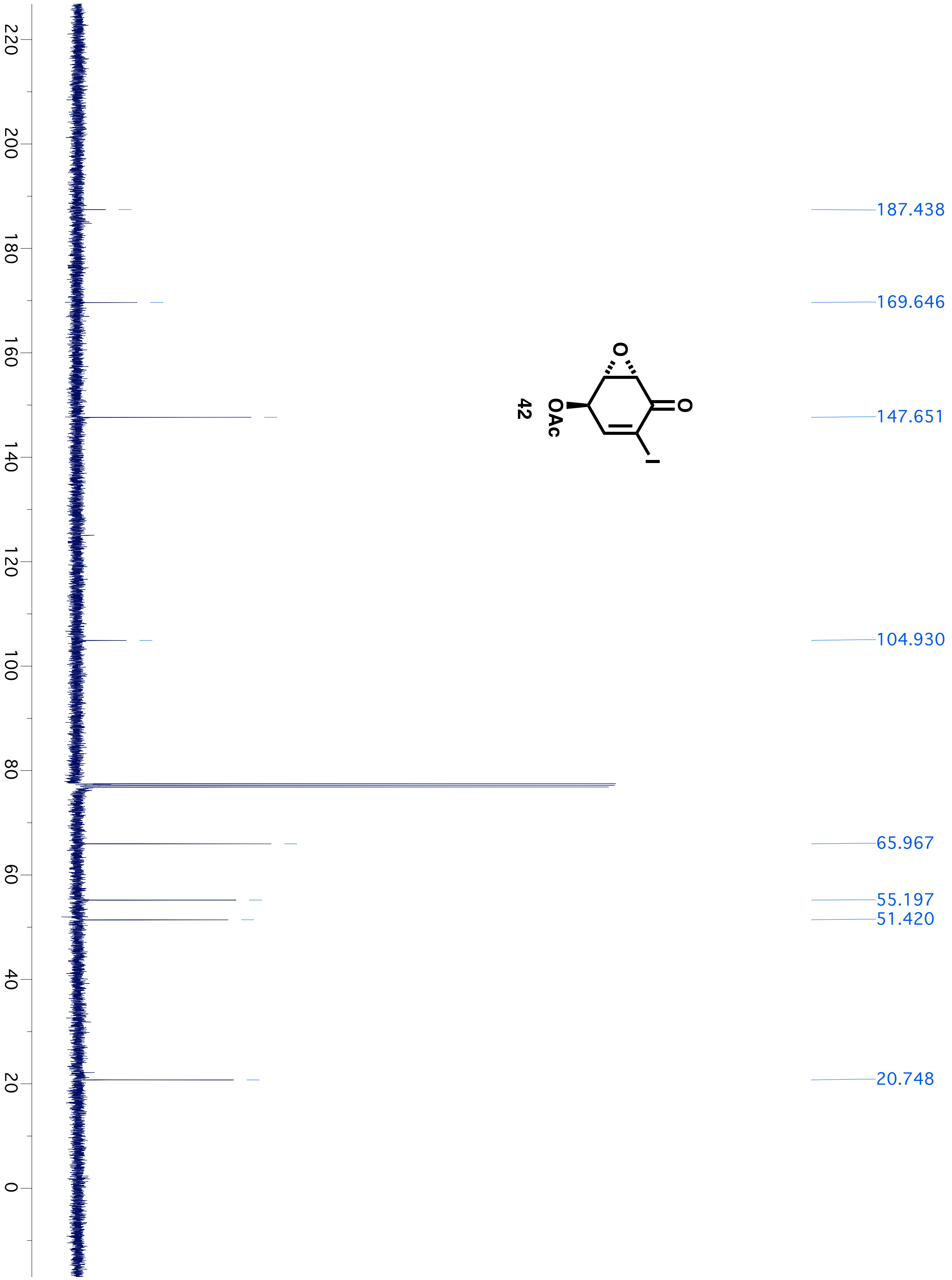

Electronic Supplementary Material (ESI) for Chemical Science
This journal is © The Royal Society of Chemistry 2014

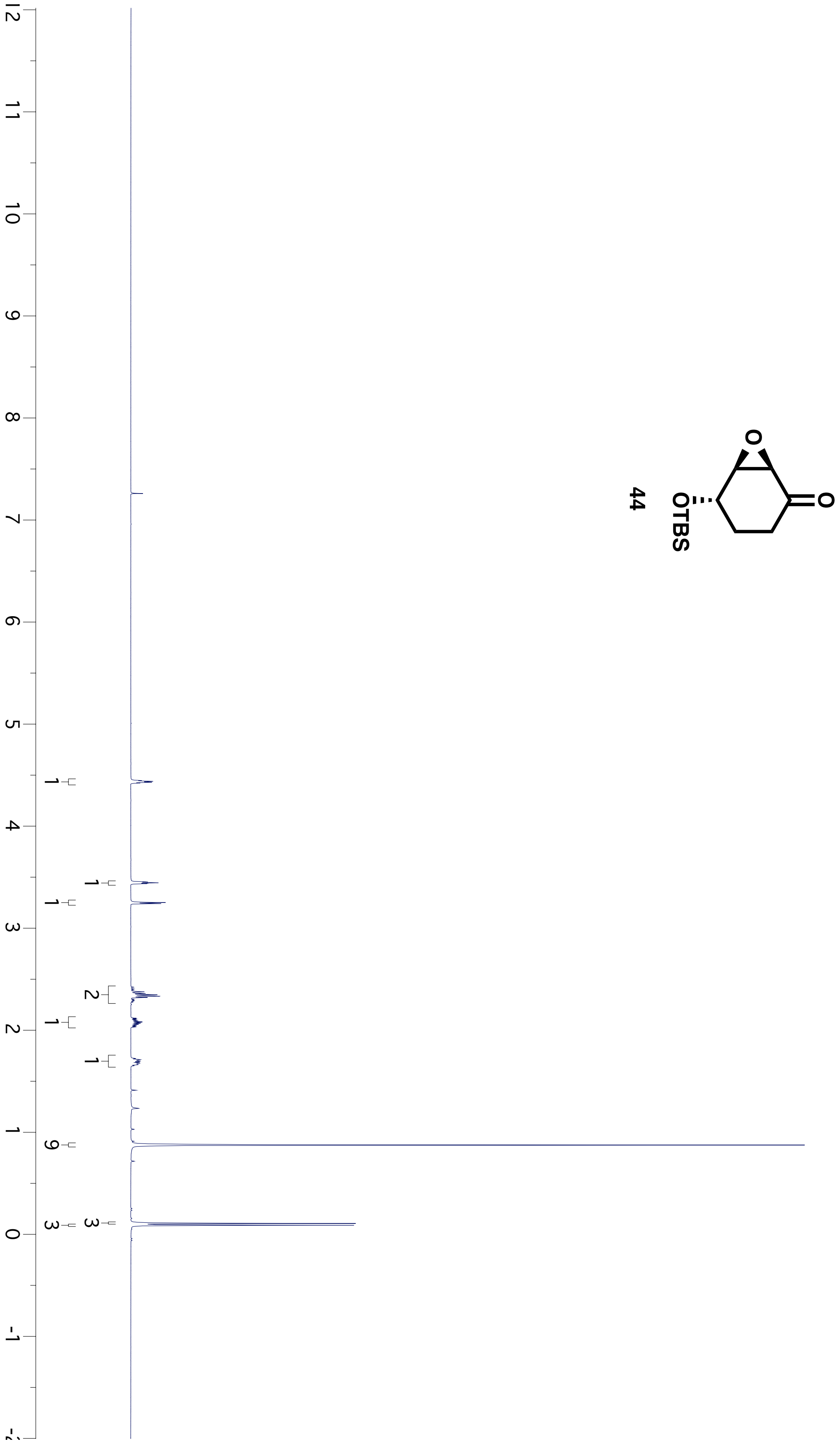

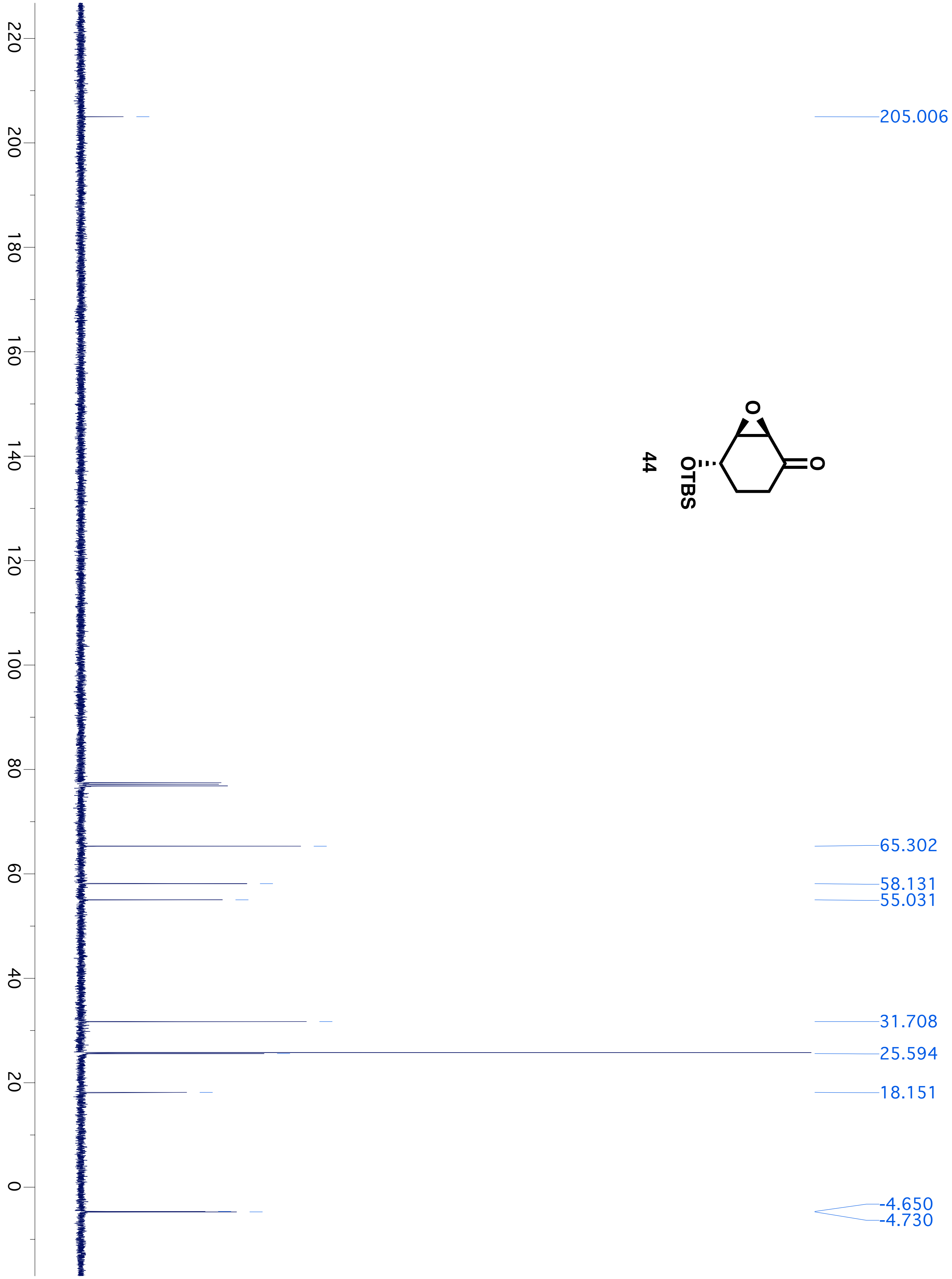
205.006
65.302
58.131
55.031
31.708
25.594
18.151
-4.650
-4.730
220
200
180
160
140
120
100
80
60
40
20
0
O
O
OTBS
44

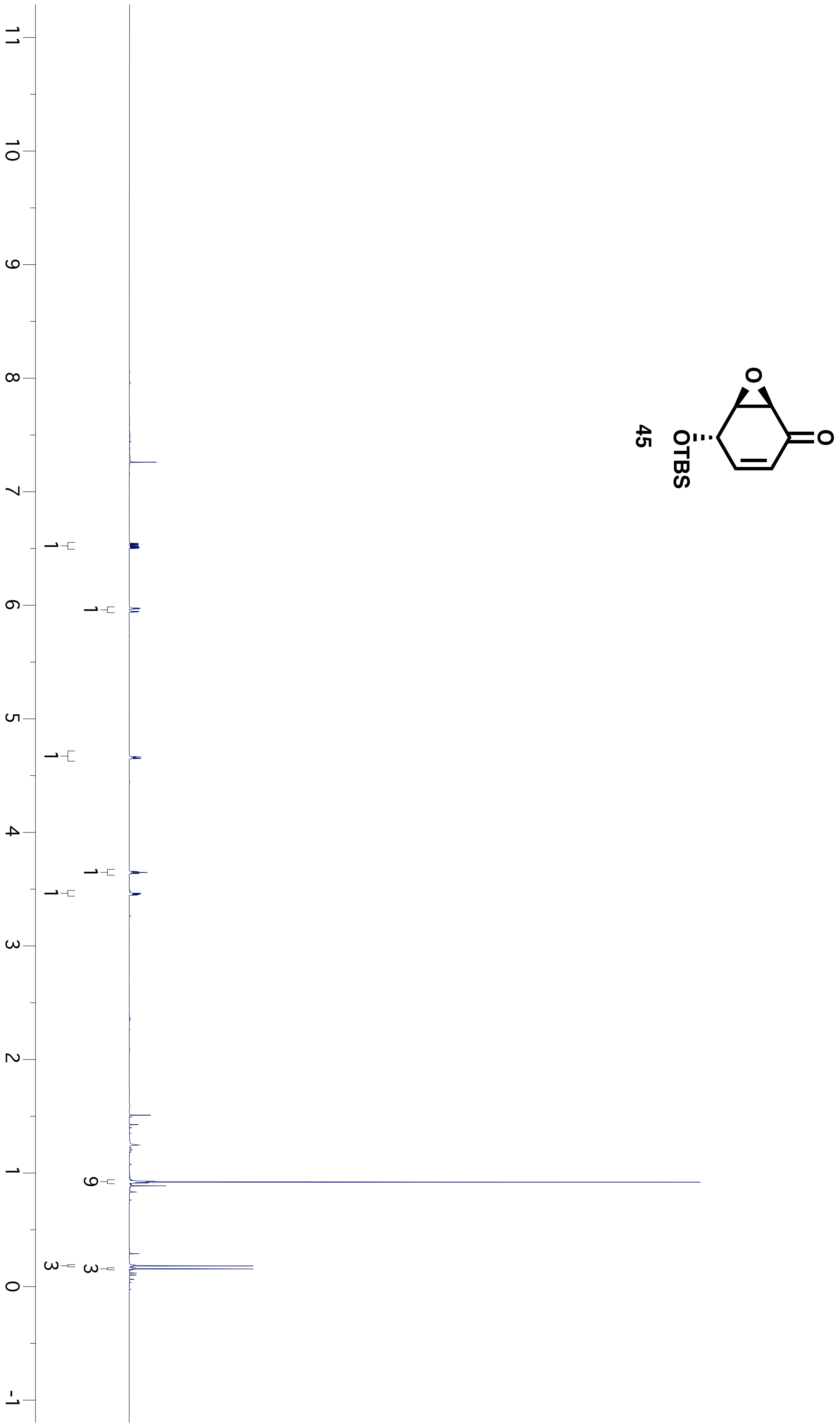
OTBS
45
O
O

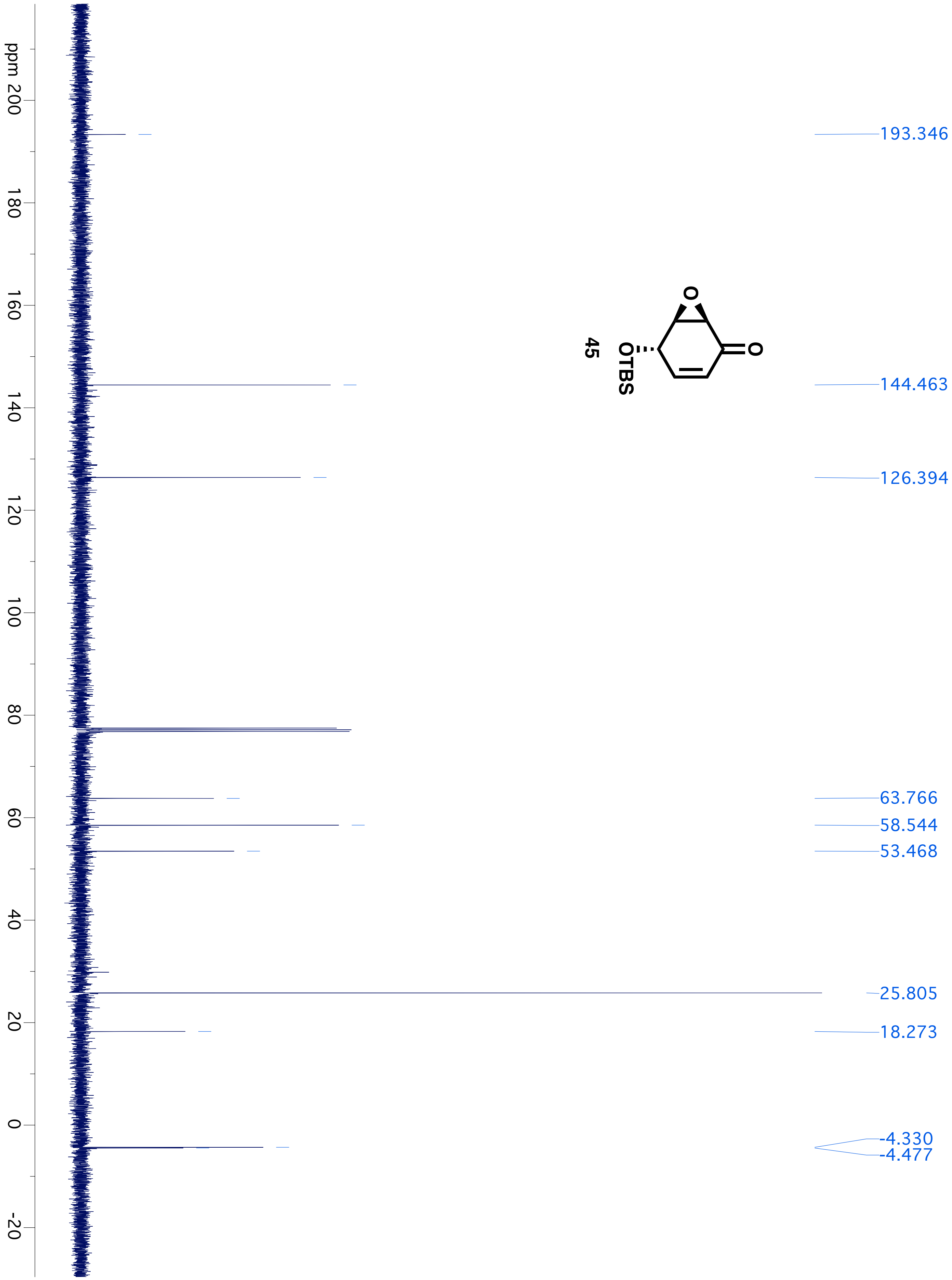
193.346
144.463
126.394
63.766
58.544
53.468
25.805
18.273
-4.330
-4.477
ppm 200
180
160
140
120
100
80
60
40
20
0
-20
O
O
OTBS
45

ple Name: jtm-21-7-pmb-rac

Method Method completed 17:45:00 09/25/13

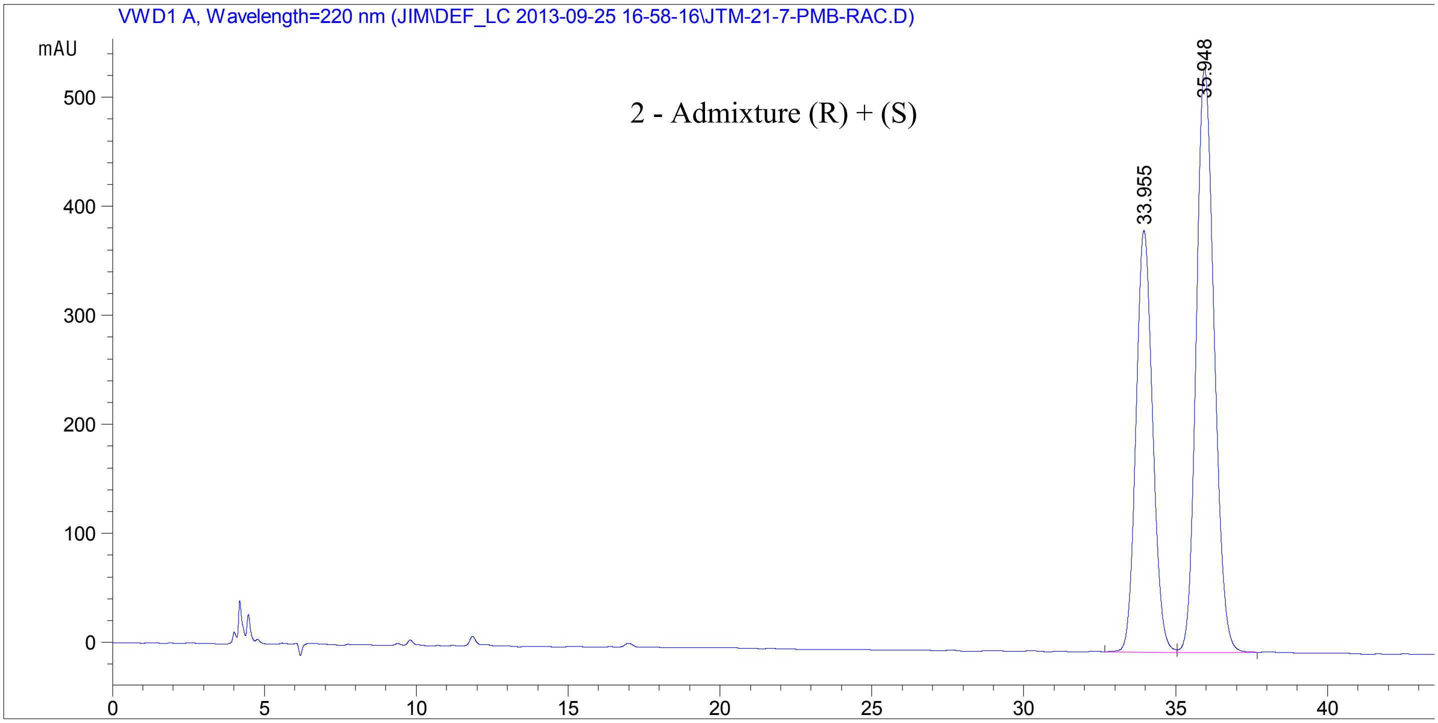

Area Percent Report

Sorted By : Signal
Multiplier: : 1.0000
Dilution: : 1.0000
Use Multiplier & Dilution Factor with ISTDs

Signal 1: VWD1 A, Wavelength=220 nm

Peak #	RetTime [min]	Type	Width [min]	Area mAU *s	Height [mAU]	Area %
1	33.955	VV	0.5937	1.47540e4	386.68399	40.1158
2	35.948	VB	0.6394	2.20245e4	536.22058	59.8842

Totals : 3.67785e4 922.90457

*** End of Report ***

ple Name: jtm-21-25

Method Method completed 14:04:04 10/10/13

===

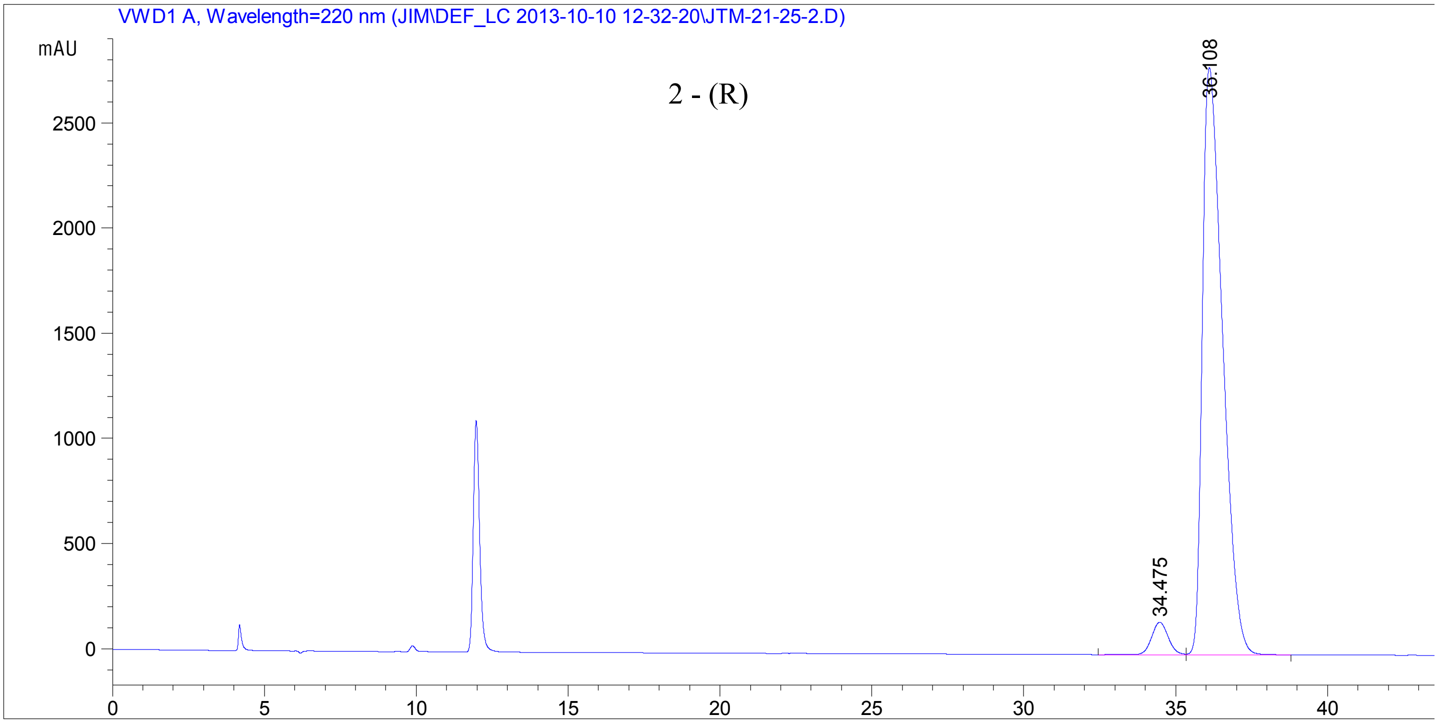

===

Area Percent Report

===

Sorted By : Signal
Multiplier: : 1.0000
Dilution: : 1.0000
Use Multiplier & Dilution Factor with ISTDs

Signal 1: VWD1 A, Wavelength=220 nm

Peak #	RetTime [min]	Type	Width [min]	Area mAU *s	Height [mAU]	Area %
1	34.475	VV	0.5897	5886.30420	154.62854	4.3601
2	36.108	VB	0.7159	1.29117e5	2790.92456	95.6399

Totals : 1.35003e5 2945.55310

===

*** End of Report ***

ple Name: jtm-21-7-pmb-s

Method Method completed 18:31:39 09/25/13

==

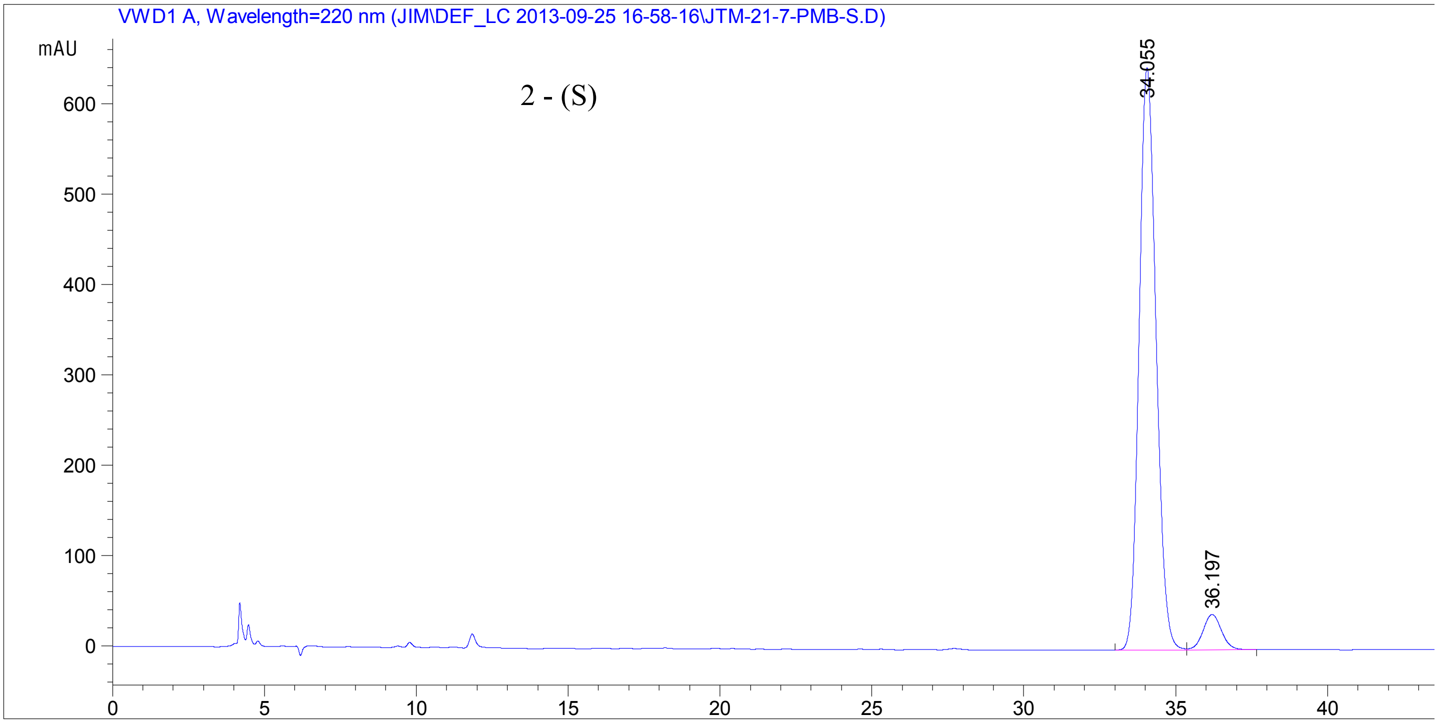

==

Area Percent Report

==

Sorted By : Signal
Multiplier: : 1.0000
Dilution: : 1.0000
Use Multiplier & Dilution Factor with ISTDs

Signal 1: VWD1 A, Wavelength=220 nm

Peak #	RetTime [min]	Type	Width [min]	Area mAU *s	Height [mAU]	Area %
1	34.055	BV	0.6033	2.49449e4	644.00079	93.7573
2	36.197	VB	0.6539	1660.93286	39.24398	6.2427

Totals : 2.66058e4 683.24478

==

*** End of Report ***

Electronic Supplementary Material (ESI) for Chemical Science
This journal is © The Royal Society of Chemistry 2014

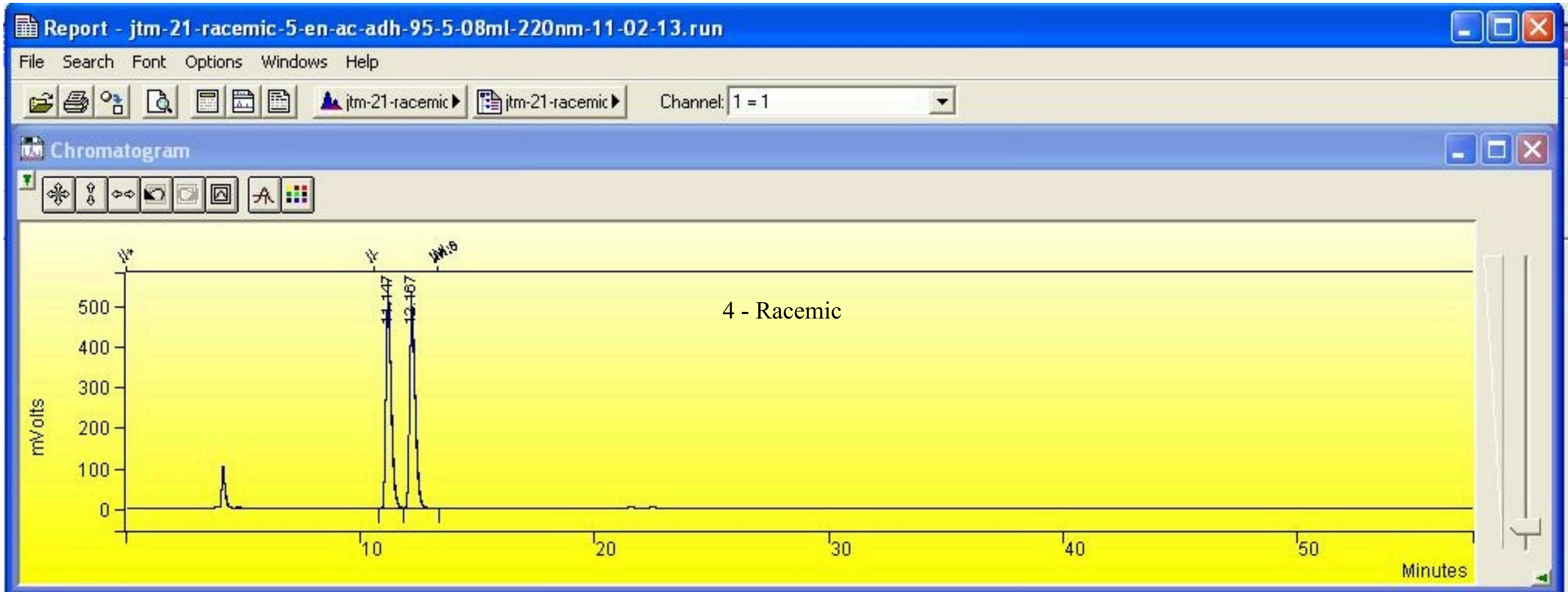

Results

Operator : Operator   Detector Type: 0800 (1 Volt)
Workstation: TROST-HPLC-LEFÿ□HÚp`   Bus Address : 80
Instrument : Instrument #1   Sample Rate : 50.00 Hz
Channel : 1 = 1   Run Time : 57.596 min

** LC Workstation Multi Instrument (Demo) Version 6.41 ** 05000-31c8-fa9-30a1 **

Run Mode : Analysis
Peak Measurement: Peak Area
Calculation Type: Percent

Peak No.	Peak Name	Result ()	Ret. Time (min)	Time Offset (min)	Area (counts)	Sep. Code	Width 1/2 (sec)	Status Codes
1		49.9342	11.147	0.000	8366827	BV	14.4	
2		50.0658	12.167	0.000	8388873	VB	15.3	

Report - jtm-21-70-adh-95-5-220nm-08ml-11-02-13.run

File Search Font Options Windows Help

jtm-21-70-adh jtm-21-70-adh Channel: 1 = 1

Chromatogram

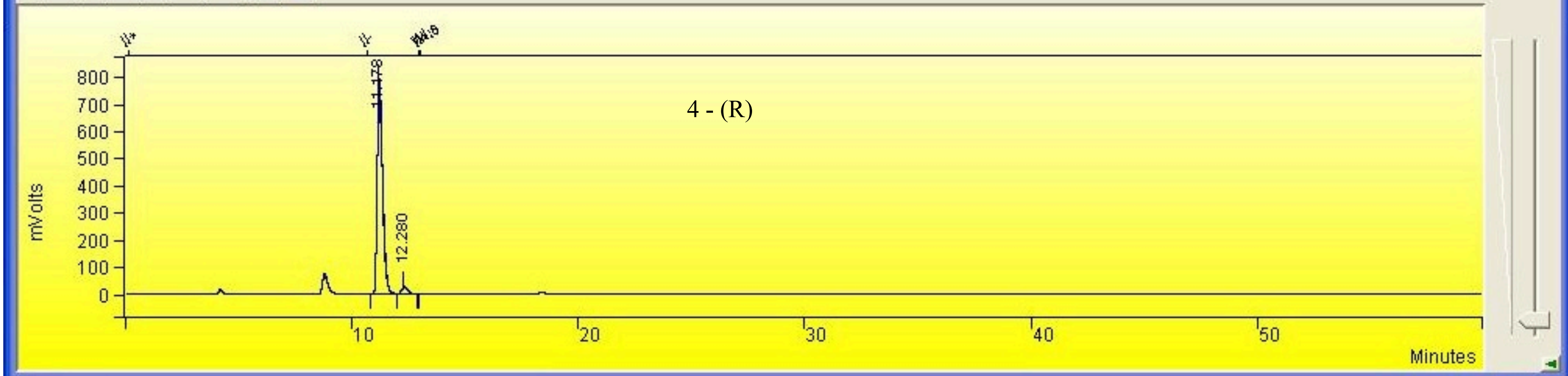

Results

```
Operator   : Operator                Detector Type: 0800 (1 Volt)
Workstation: TROST-HPLC-LEFÿ□HÚþ``   Bus Address  : 80
Instrument : Instrument #1           Sample Rate  : 50.00 Hz
Channel    : 1 = 1                   Run Time     : 60.000 min

** LC Workstation Multi Instrument (Demo) Version 6.41 ** 05000-31c8-fa9-30a1 **

Run Mode         : Analysis
Peak Measurement: Peak Area
Calculation Type: Percent
```

Peak No.	Peak Name	Result ()	Ret. Time (min)	Time Offset (min)	Area (counts)	Sep. Code	Width 1/2 (sec)	Status Codes
1		96.5242	11.178	0.000	13062313	BB	14.8	
2		3.4758	12.280	0.000	470362	TS	0.0	

Electronic Supplementary Material (ESI) for Chemical Science
This journal is © The Royal Society of Chemistry 2014

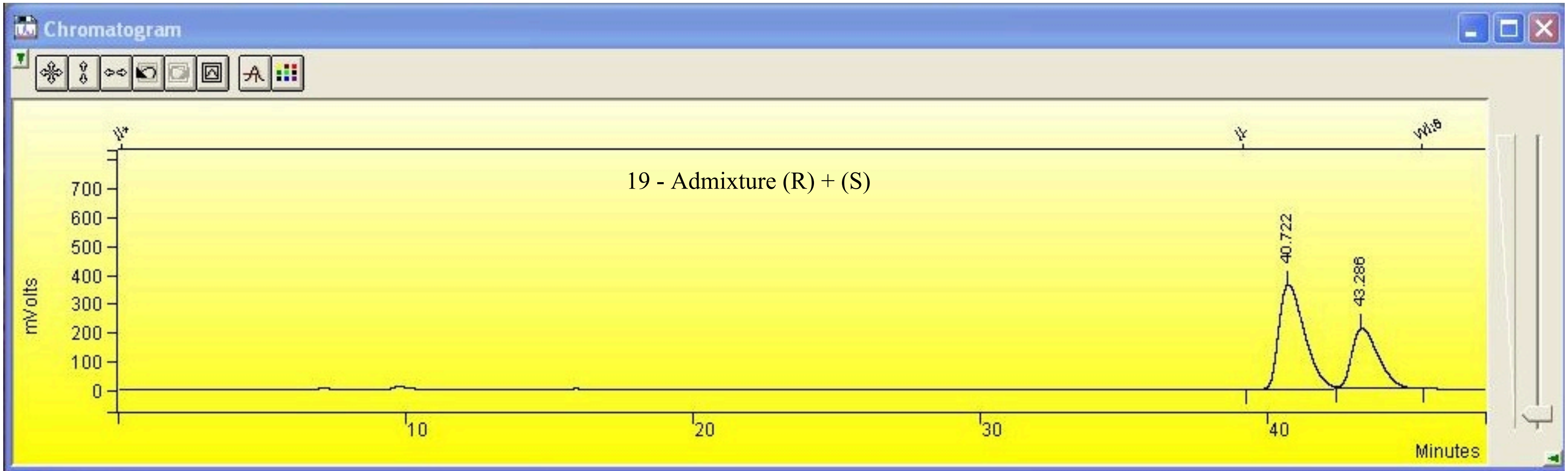

Results

```
Operator   : Operator               Detector Type: 0800 (1 Volt)
Workstation: TROST-HPLC-LEFÿ□HÚp`    Bus Address  : 80
Instrument : Instrument #1          Sample Rate  : 50.00 Hz
Channel    : 1 = 1                  Run Time     : 47.683 min

** LC Workstation Multi Instrument (Demo) Version 6.41 ** 05000-31c8-fa9-30a1 **

Run Mode        : Analysis
Peak Measurement: Peak Area
Calculation Type: Percent
```

Peak No.	Peak Name	Result ()	Ret. Time (min)	Time Offset (min)	Area (counts)	Sep. Code	Width 1/2 (sec)	Status Codes
1		62.6355	40.722	0.000	22667510	BV	65.3	
2		37.3645	43.286	0.000	13522044	VB	67.7	
	Totals:	100.0000		0.000	36189554			

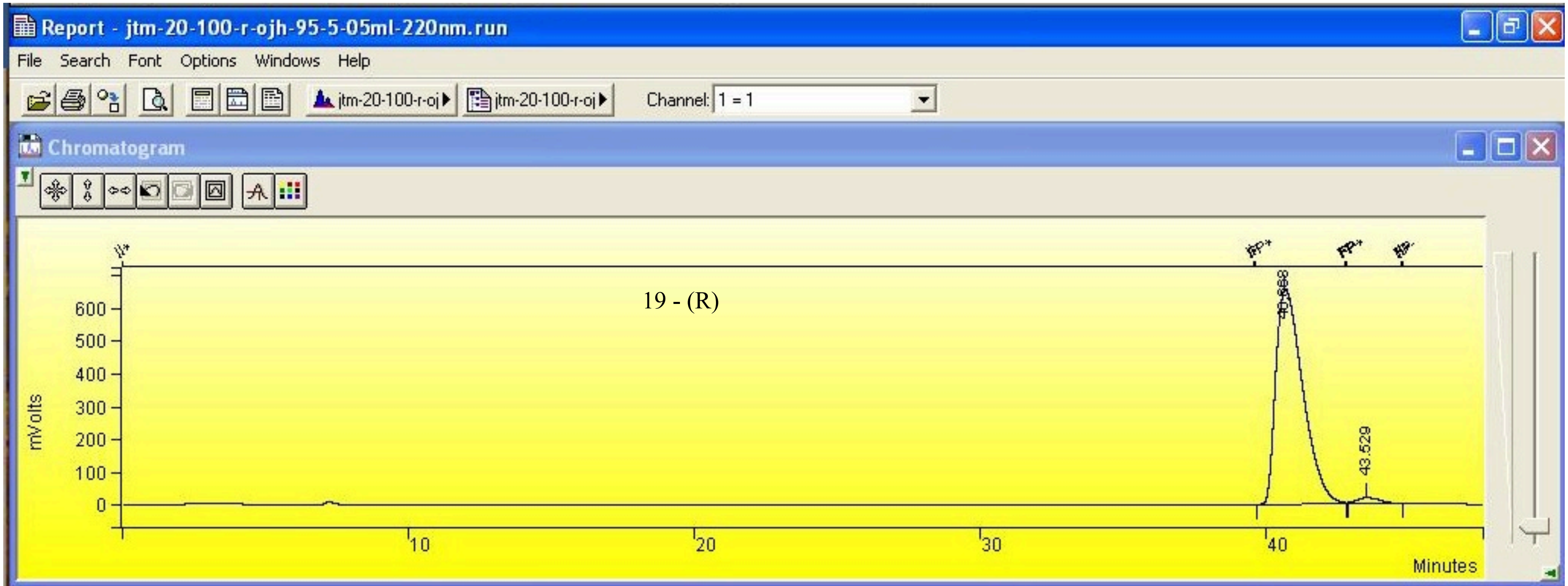

Results

```
Operator   : Operator                Detector Type: 0800 (1 Volt)
Workstation: TROST-HPLC-LEFÿ□HÚþ`□   Bus Address  : 80
Instrument : Instrument #1           Sample Rate  : 50.00 Hz
Channel    : 1 = 1                   Run Time     : 47.666 min

** LC Workstation Multi Instrument (Demo) Version 6.41 ** 05000-31c8-fa9-30a1 **

Run Mode        : Analysis
Peak Measurement: Peak Area
Calculation Type: Percent
```

Peak No.	Peak Name	Result ()	Ret. Time (min)	Time Offset (min)	Area (counts)	Sep. Code	Width 1/2 (sec)	Status Codes
1		97.9866	40.668	0.000	43152128	BB	62.1	
2		2.0134	43.529	0.000	886689	BB	47.8	
	Totals:	100.0000		0.000	44038817			

Report - jtm-20-100-s-ojh-95-5-05ml-220nm.run

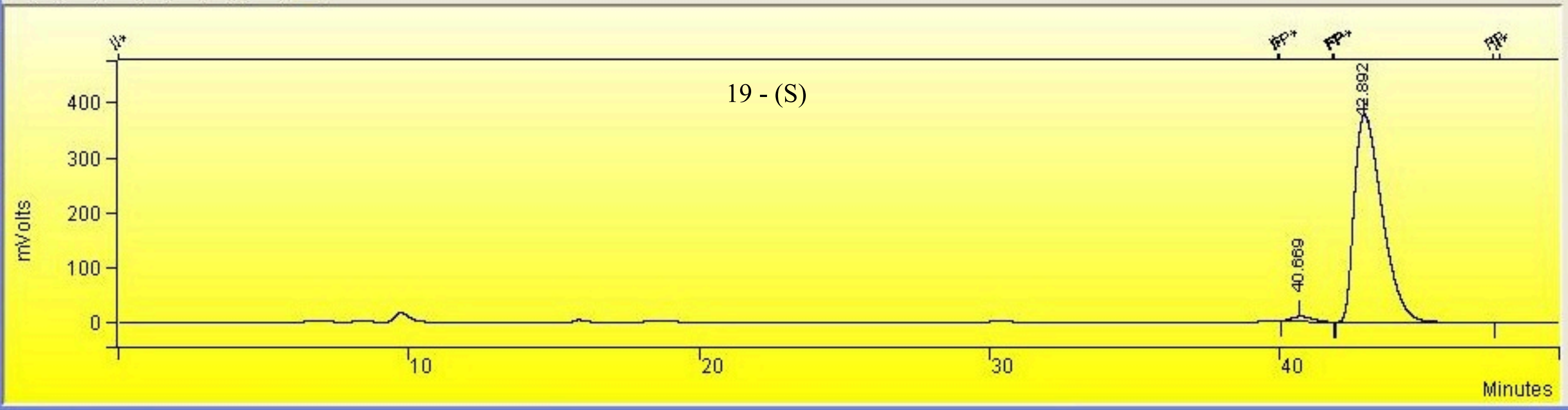

Results

```
Operator    : Operator              Detector Type: 0800 (1 Volt)
Workstation: TROST-HPLC-LEFÿ□HÚþ`   Bus Address  : 80
Instrument : Instrument #1          Sample Rate  : 50.00 Hz
Channel    : 1 = 1                  Run Time     : 49.684 min

** LC Workstation Multi Instrument (Demo) Version 6.41 ** 05000-31c8-fa9-30a1 **

Run Mode         : Analysis
Peak Measurement: Peak Area
Calculation Type: Percent
```

Peak No.	Peak Name	Result ()	Ret. Time (min)	Time Offset (min)	Area (counts)	Sep. Code	Width 1/2 (sec)	Status Codes
1		1.7286	40.669	0.000	443737	BB	43.5	
2		98.2714	42.892	0.000	25226274	BB	62.1	
	Totals:	100.0000		0.000	25670011			

Electronic Supplementary Material (ESI) for Chemical Science
This journal is © The Royal Society of Chemistry 2014

Data File C:\CHEM32\1\DATA\JIM\DEF_LC 2013-08-20 19-20-27\JTM-20-53-RAC-IC-90-10.D

Sample Name: jtm-20-53-rac

Method Method completed 20:07:09 08/20/13

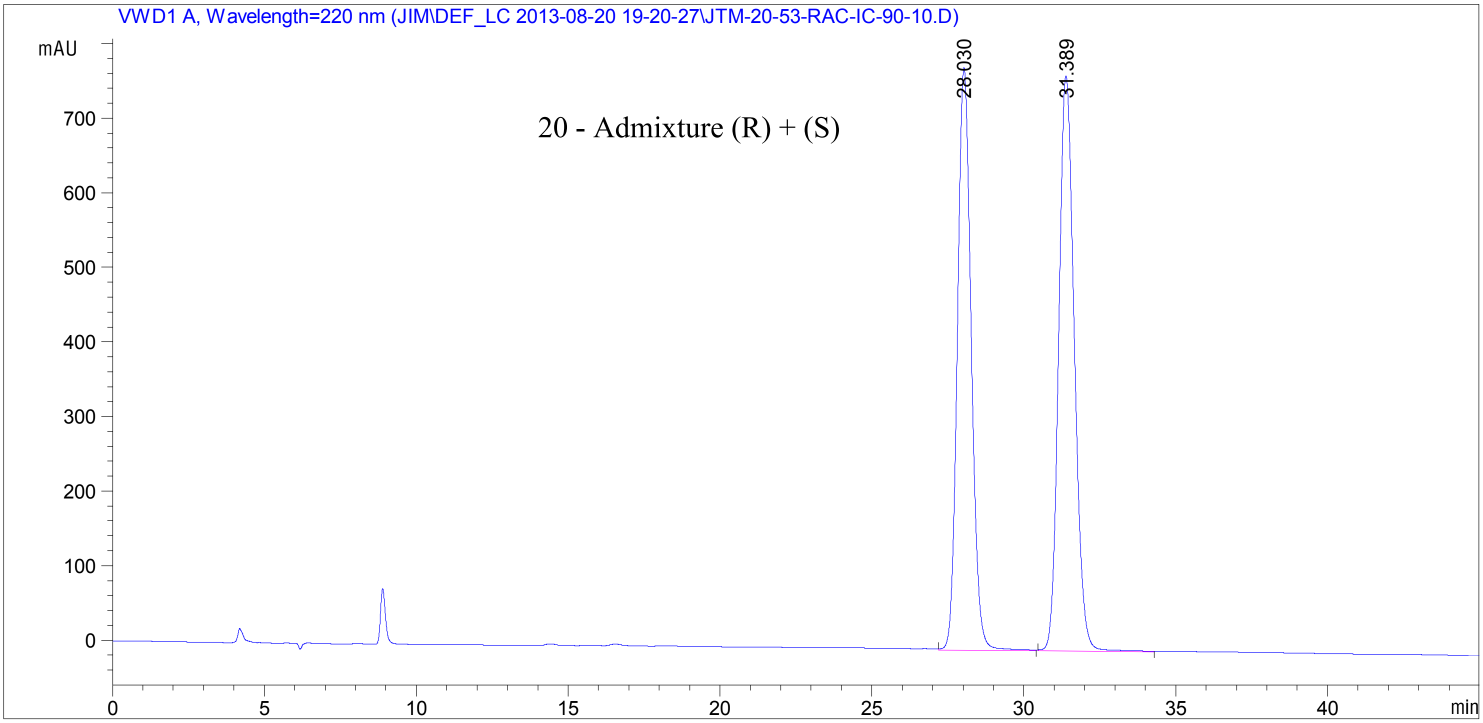

Area Percent Report

Sorted By : Signal
Multiplier: : 1.0000
Dilution: : 1.0000
Use Multiplier & Dilution Factor with ISTDs

Signal 1: VWD1 A, Wavelength=220 nm

Peak #	RetTime [min]	Type	Width [min]	Area mAU *s	Height [mAU]	Area %
1	28.030	VB	0.4926	2.47872e4	780.87238	47.2756
2	31.389	BB	0.5549	2.76441e4	771.46741	52.7244

Totals : 5.24314e4 1552.33978

*** End of Report ***

Data File C:\CHEM32\1\DATA\JIM\DEF_LC 2013-08-20 19-20-27\JTM-20-25-R-IC-90-10.D
Sample Name: jtm-20-25-R

Method Method completed 20:53:52 08/20/13

===

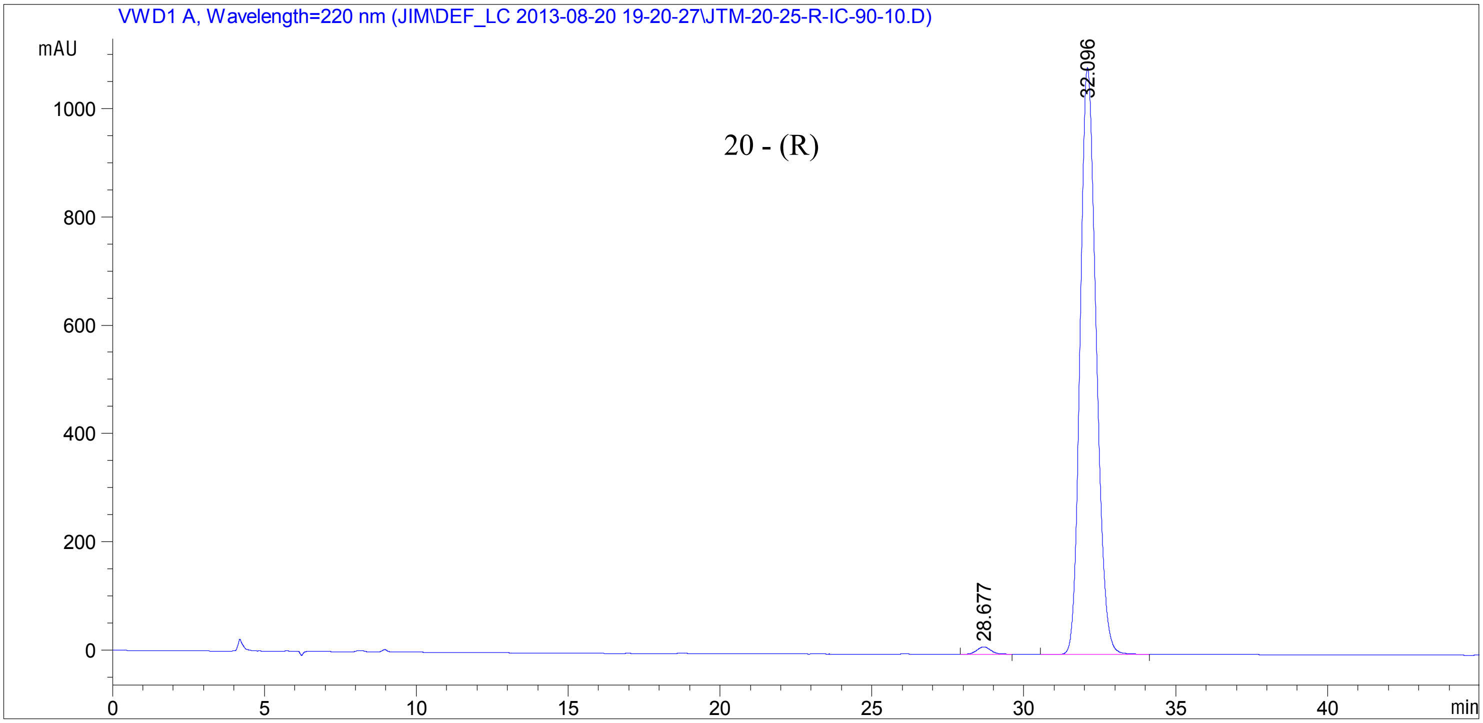

===
Area Percent Report
===

Sorted By : Signal
Multiplier: : 1.0000
Dilution: : 1.0000
Use Multiplier & Dilution Factor with ISTDs

Signal 1: VWD1 A, Wavelength=220 nm

Peak #	RetTime [min]	Type	Width [min]	Area mAU *s	Height [mAU]	Area %
1	28.677	VV	0.5317	491.20615	14.09659	1.2235
2	32.096	VB	0.5698	3.96559e4	1083.23499	98.7765

Totals : 4.01471e4 1097.33157

===
*** End of Report ***

Electronic Supplementary Material (ESI) for Chemical Science
This journal is © The Royal Society of Chemistry 2014
Data File C:\CHEM32\1\DATA\JIM\DEF_LC 2013-08-20 19-20-27\JTM-20-53-S-IC-90-10.D
Sample Name: jtm-20-53-S

Method Method completed 21:40:31 08/20/13

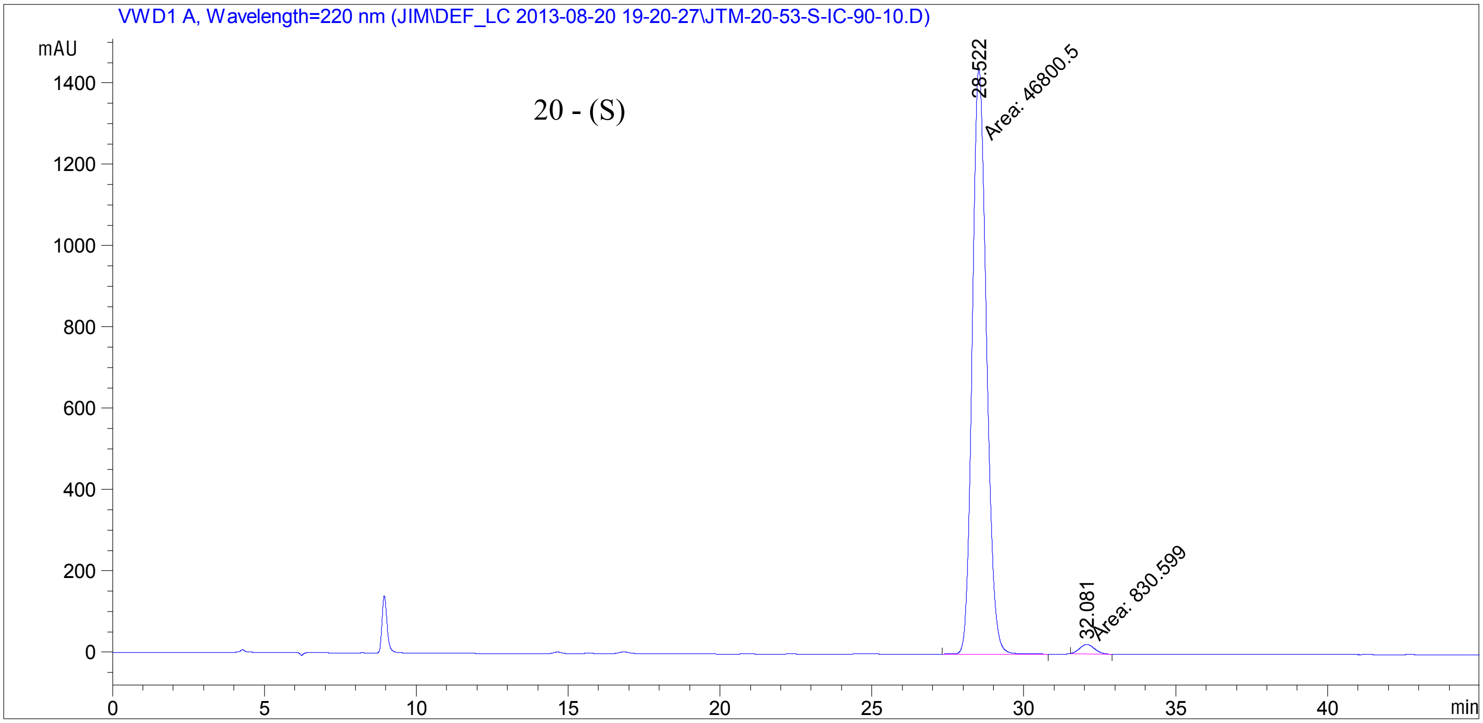

Area Percent Report

Sorted By : Signal
Multiplier: : 1.0000
Dilution: : 1.0000
Use Multiplier & Dilution Factor with ISTDs

Signal 1: VWD1 A, Wavelength=220 nm

Peak #	RetTime [min]	Type	Width [min]	Area mAU *s	Height [mAU]	Area %
1	28.522	MM	0.5407	4.68005e4	1442.52966	98.2562
2	32.081	MM	0.5856	830.59882	23.63828	1.7438

Totals : 4.76311e4 1466.16794

*** End of Report ***

Data File C:\CHEM32\1\DATA\JIM\DEF_LC 2013-09-02 12-14-59\JTM-20-85-RAC-IC-97-3.D
Sample Name: jtm-20-85-RAC

Method   Method stopped by user   12:50:50 09/02/13

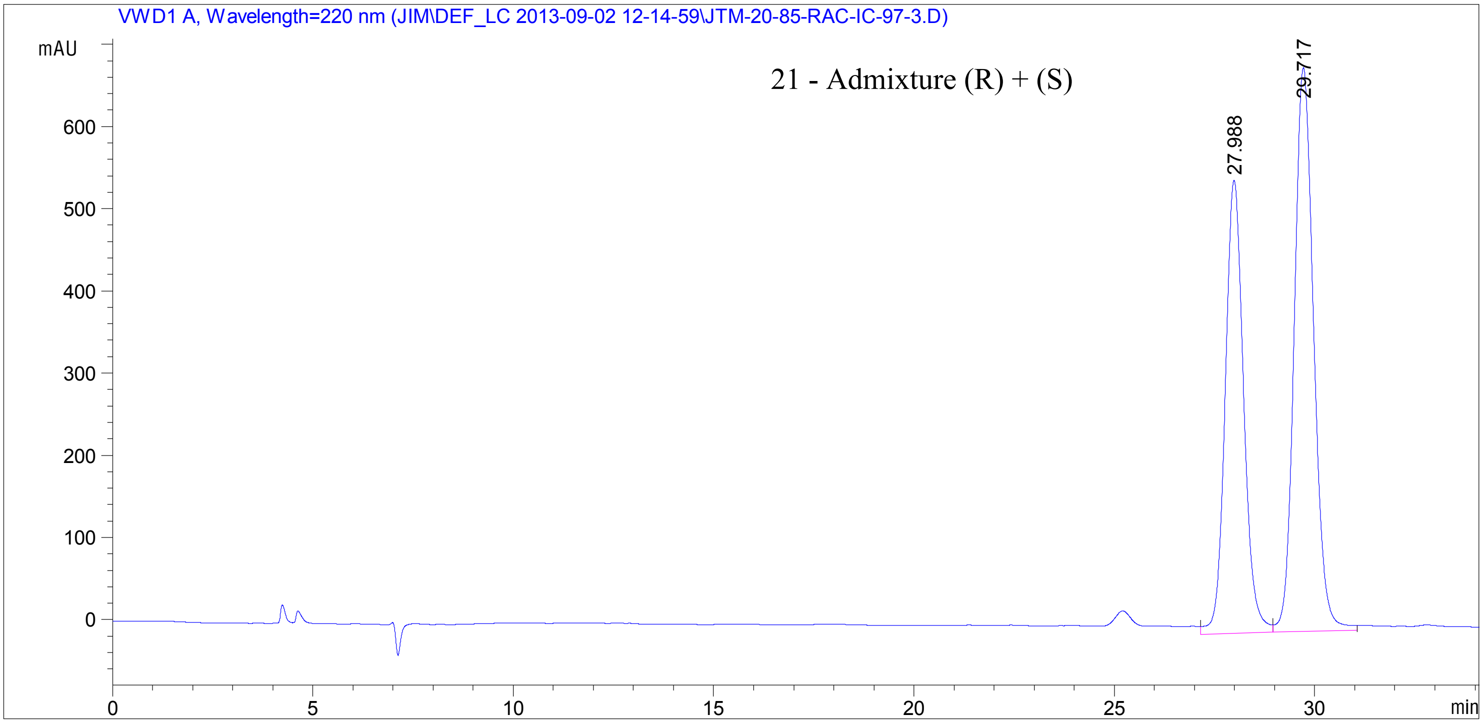

Area Percent Report

Sorted By : Signal
Multiplier: : 1.0000
Dilution: : 1.0000
Use Multiplier & Dilution Factor with ISTDs

Signal 1: VWD1 A, Wavelength=220 nm

Peak #	RetTime [min]	Type	Width [min]	Area mAU *s	Height [mAU]	Area %
1	27.988	VV	0.4945	1.77155e4	551.05151	43.2881
2	29.717	VV	0.5230	2.32091e4	685.66119	56.7119

Totals : 4.09245e4 1236.71271

*** End of Report ***

Electronic Supplementary Material (ESI) for Chemical Science
This journal is © The Royal Society of Chemistry 2014

Data File C:\CHEM32\1\DATA\JIM\DEF_LC 2013-09-16 18-39-01\JTM-20-96-2BR.D
Sample Name: jtm-20-96-2br

Method Method completed 20:42:35 09/16/13

===

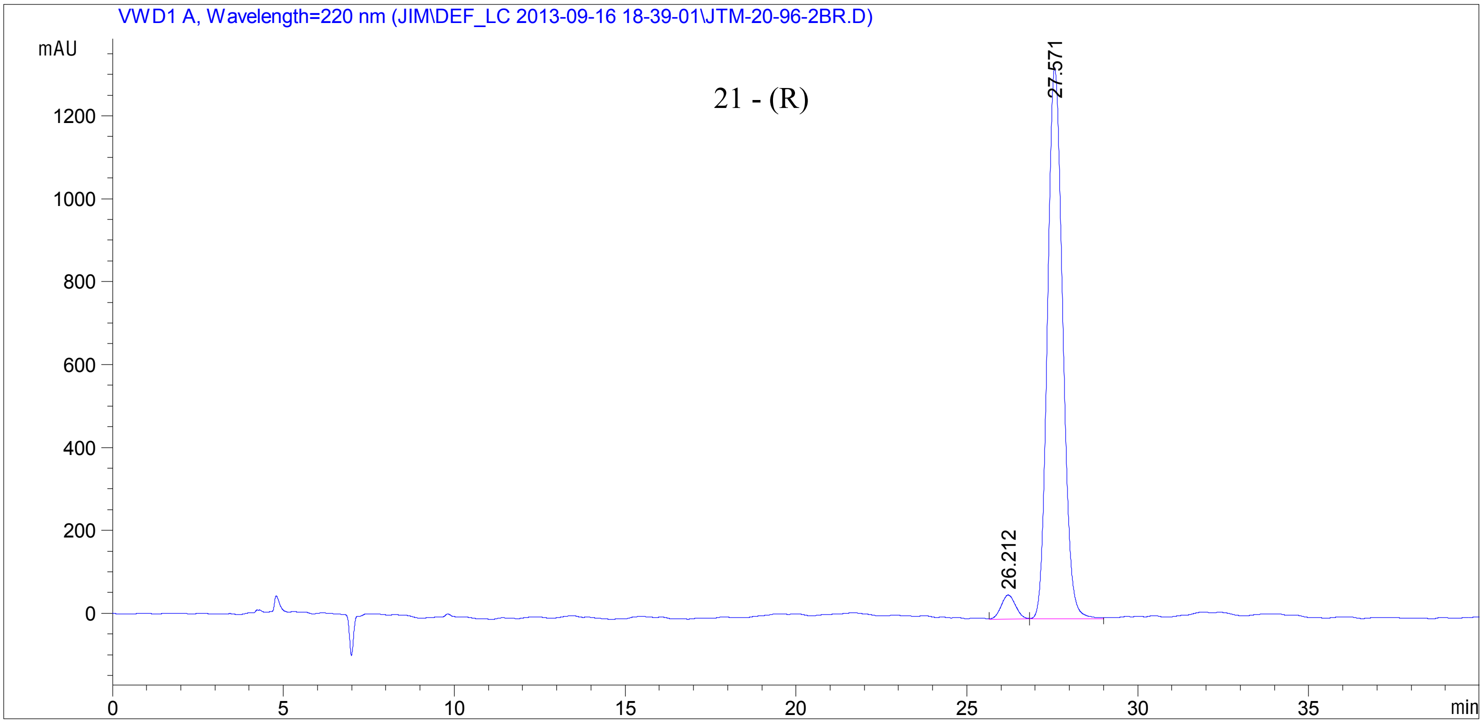

===

Area Percent Report

===

Sorted By : Signal
Multiplier: : 1.0000
Dilution: : 1.0000
Use Multiplier & Dilution Factor with ISTDs

Signal 1: VWD1 A, Wavelength=220 nm

Peak #	RetTime [min]	Type	Width [min]	Area mAU *s	Height [mAU]	Area %
1	26.212	VV	0.4557	1706.60278	58.19334	3.9701
2	27.571	VV	0.4844	4.12799e4	1329.99402	96.0299

Totals : 4.29865e4 1388.18736

===

*** End of Report ***

Data File C:\CHEM32\1\DATA\JIM\DEF_LC 2013-09-02 13-32-08\JTM-20-85-S.D
Sample Name: jtm-20-85-S

Method Method stopped by user 14:13:07 09/02/13

===

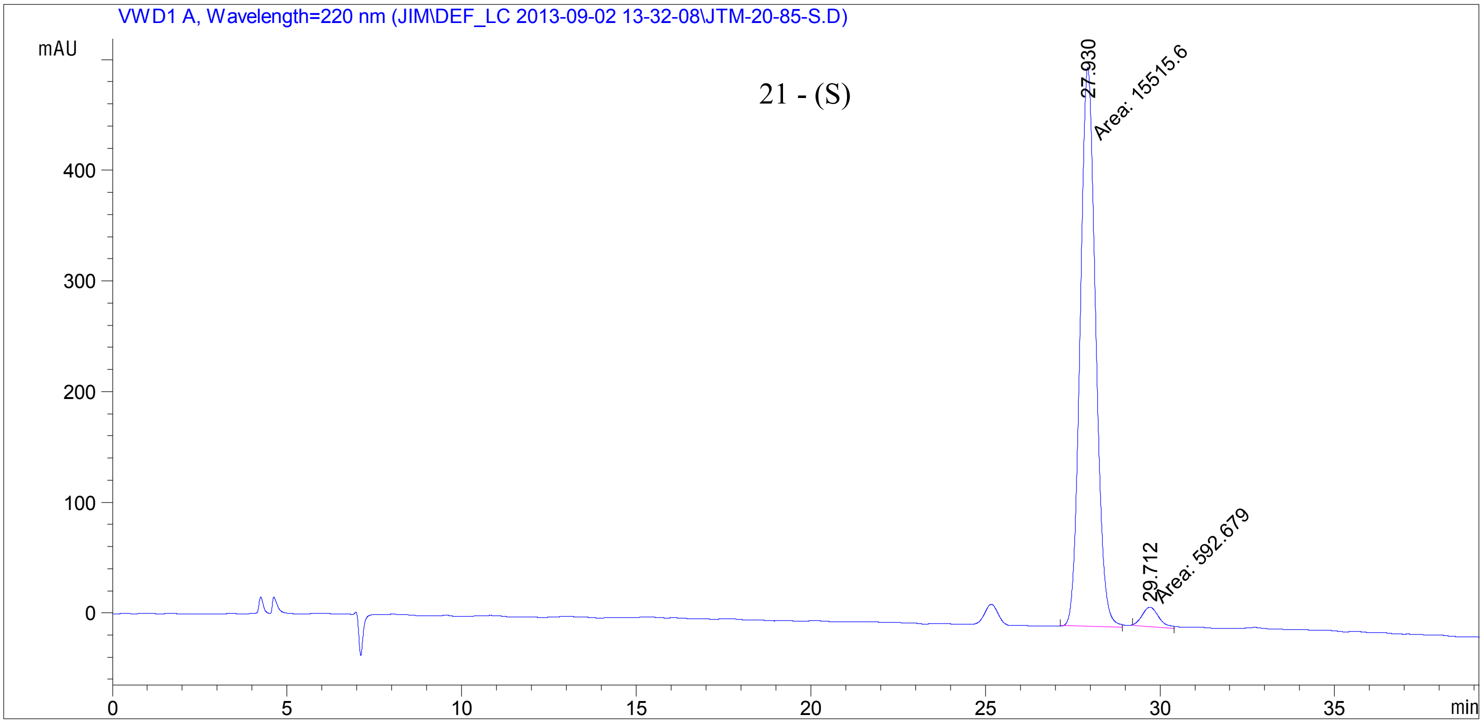

===

Area Percent Report

===

Sorted By : Signal
Multiplier: : 1.0000
Dilution: : 1.0000
Use Multiplier & Dilution Factor with ISTDs

Signal 1: VWD1 A, Wavelength=220 nm

Peak #	RetTime [min]	Type	Width [min]	Area mAU *s	Height [mAU]	Area %
1	27.930	MM	0.5126	1.55156e4	504.45609	96.3207
2	29.712	MM	0.5590	592.67914	17.67150	3.6793

Totals : 1.61083e4 522.12758

===

*** End of Report ***

Electronic Supplementary Material (ESI) for Chemical Science
This journal is © The Royal Society of Chemistry 2014
Data File C:\CHEM32\1\DATA\JIM\DEF_LC 2013-08-16 14-58-35\JTM-20-50-RAC-RUN2.D
Sample Name: jtm-20-50-rac

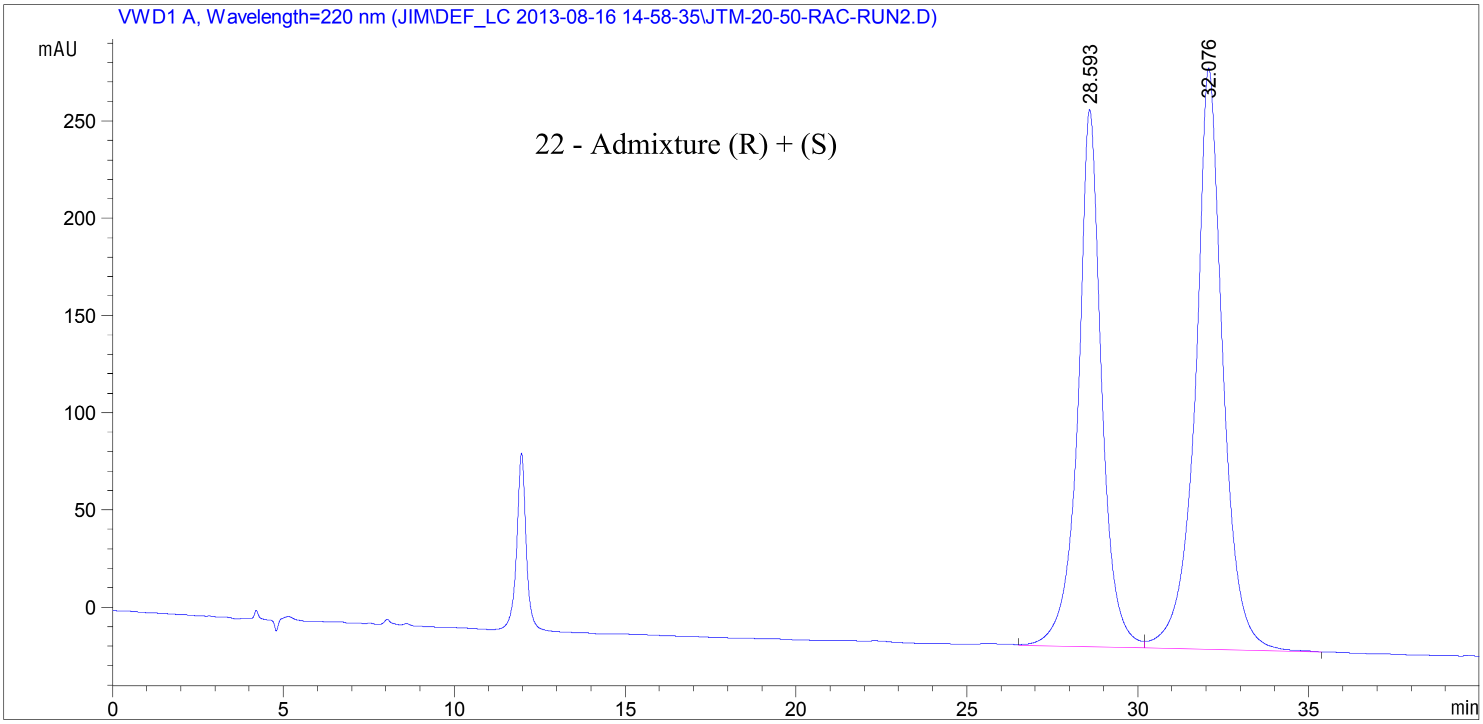

Area Percent Report

Sorted By : Signal
Multiplier: : 1.0000
Dilution: : 1.0000
Use Multiplier & Dilution Factor with ISTDs

Signal 1: VWD1 A, Wavelength=220 nm

Peak #	RetTime [min]	Type	Width [min]	Area mAU *s	Height [mAU]	Area %
1	28.593	VV	0.6977	1.33518e4	276.30225	44.1793
2	32.076	VB	0.8098	1.68700e4	298.92535	55.8207
Totals :				3.02218e4	575.22760	

*** End of Report ***

Electronic Supplementary Material (ESI) for Chemical Science
This journal is © The Royal Society of Chemistry 2014

===

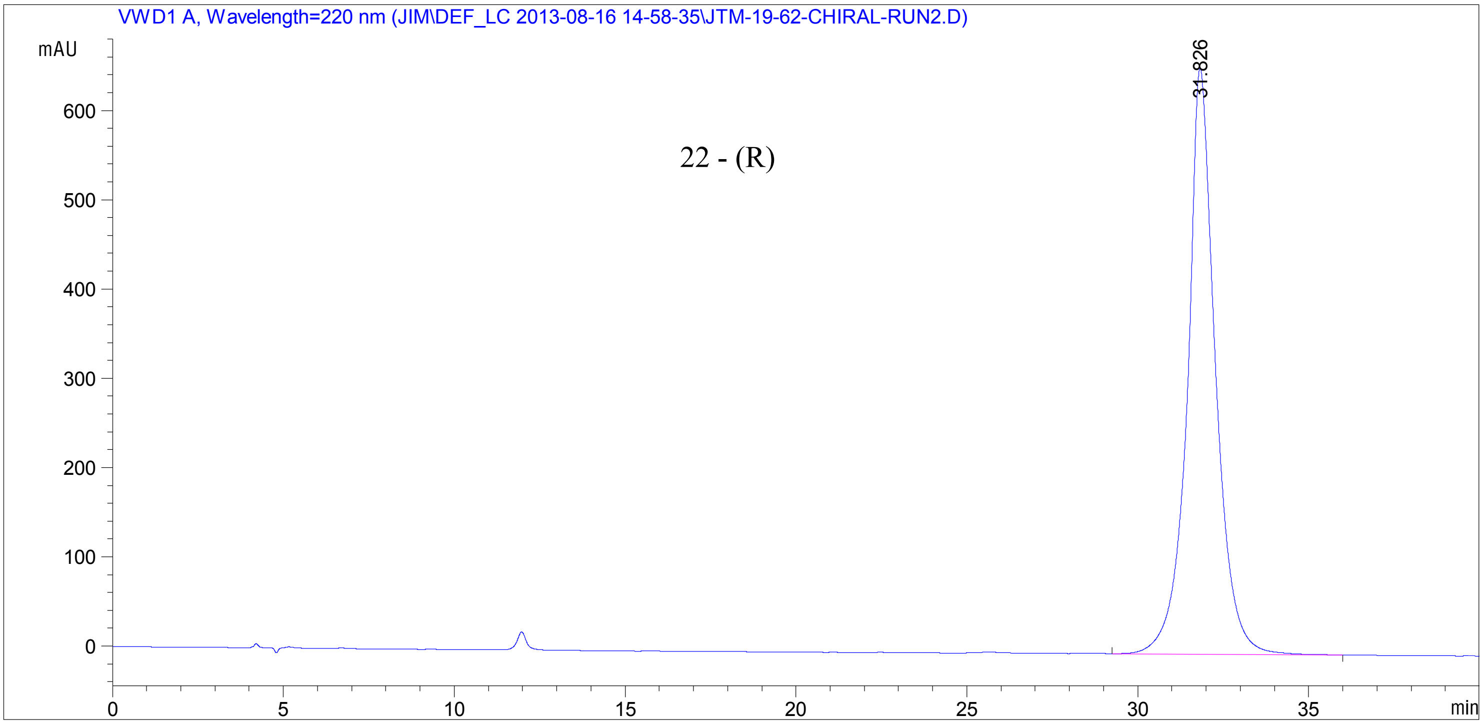

===

Area Percent Report

===

Sorted By : Signal
Multiplier: : 1.0000
Dilution: : 1.0000
Use Multiplier & Dilution Factor with ISTDs

Signal 1: VWD1 A, Wavelength=220 nm

Peak #	RetTime [min]	Type	Width [min]	Area mAU *s	Height [mAU]	Area %
1	31.826	VV	0.8156	3.75540e4	656.75989	100.0000

Totals : 3.75540e4 656.75989

===

*** End of Report ***

Electronic Supplementary Material (ESI) for Chemical Science
This journal is © The Royal Society of Chemistry 2014
Data File C:\CHEM32\1\DATA\JIM\DEF_LC 2013-08-16 14-58-35\JTM-20-50-CHIRAL.D
Sample Name: jtm-20-50-chiral-S

===

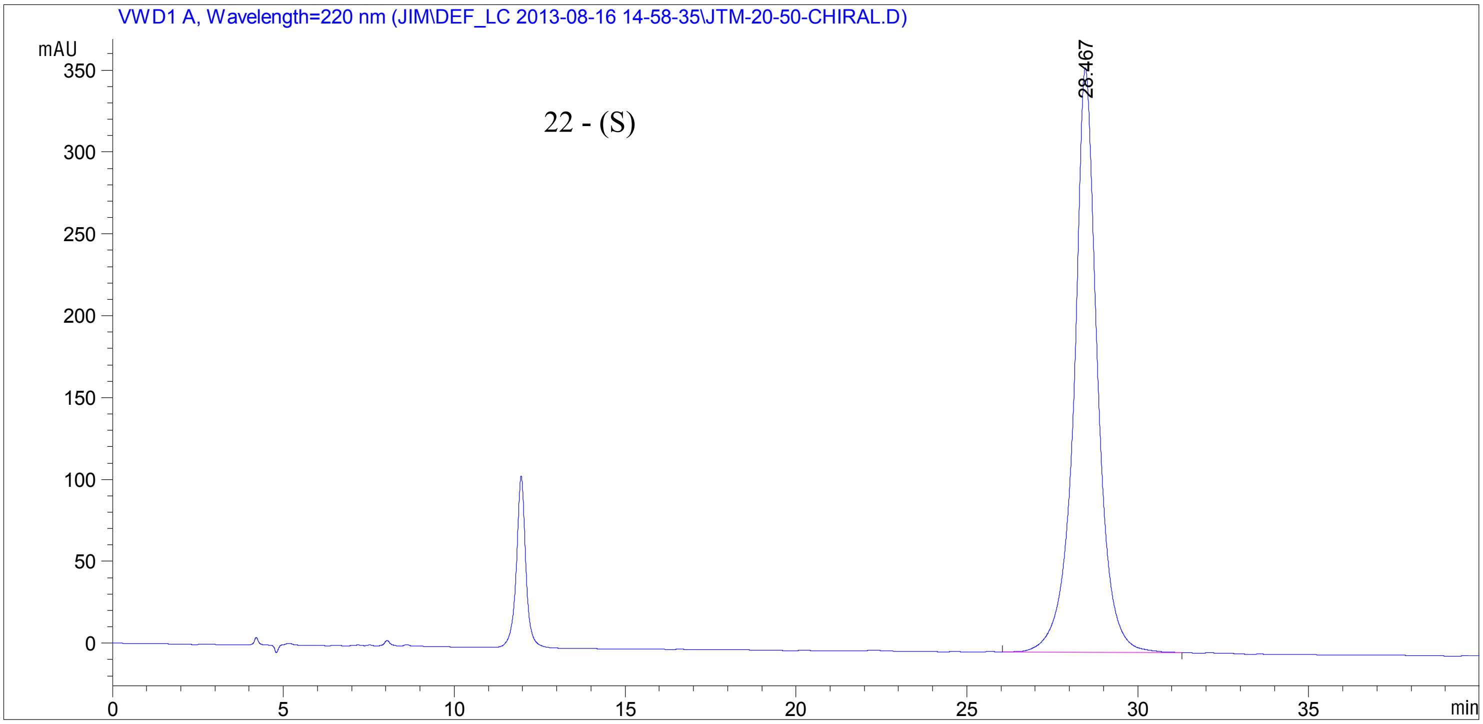

===
Area Percent Report
===

Sorted By : Signal
Multiplier: : 1.0000
Dilution: : 1.0000
Use Multiplier & Dilution Factor with ISTDs

Signal 1: VWD1 A, Wavelength=220 nm

Peak #	RetTime [min]	Type	Width [min]	Area mAU *s	Height [mAU]	Area %
1	28.467	VB	0.6937	1.71254e4	356.93121	100.0000

Totals : 1.71254e4 356.93121

===
*** End of Report ***

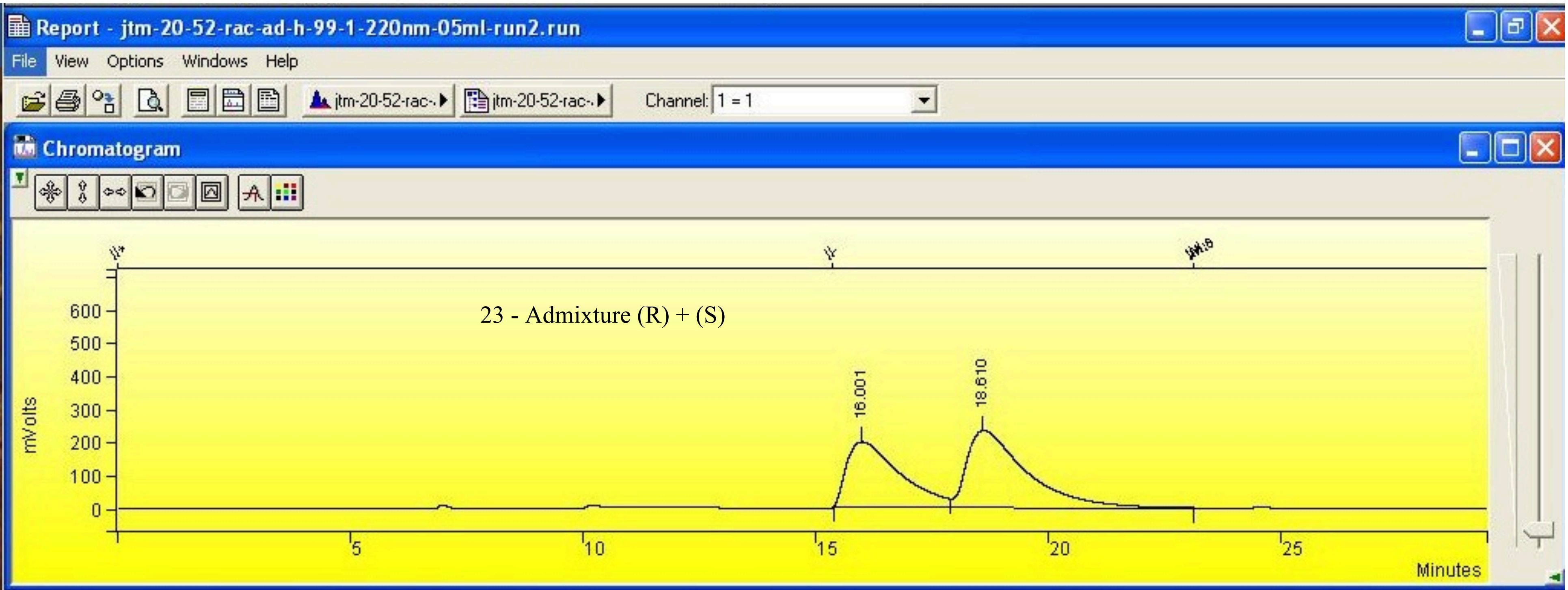

Results

```
Operator   : Operator                 Detector Type: 0800 (1 Volt)
Workstation: TROST-HPLC-LEFÿ□HÚþ`□    Bus Address  : 80
Instrument : Instrument #1            Sample Rate  : 50.00 Hz
Channel    : 1 = 1                    Run Time     : 29.491 min

** LC Workstation Multi Instrument (Demo) Version 6.41 ** 05000-31c8-fa9-30a1 **

Run Mode         : Analysis
Peak Measurement: Peak Area
Calculation Type: Percent
```

Peak No.	Peak Name	Result ()	Ret. Time (min)	Time Offset (min)	Area (counts)	Sep. Code	Width 1/2 (sec)	Status Codes
1		43.3250	16.001	0.000	15549590	BV	91.1	
2		56.6750	18.610	0.000	20341000	VB	94.4	
	Totals:	100.0000		0.000	35890590			

Report - jtm-20-48-r-ad-h-99-1-220nm-05ml.run

File View Options Windows Help

jtm-20-48-r-ad· jtm-20-52-rac-· Channel: 1 = 1

Chromatogram

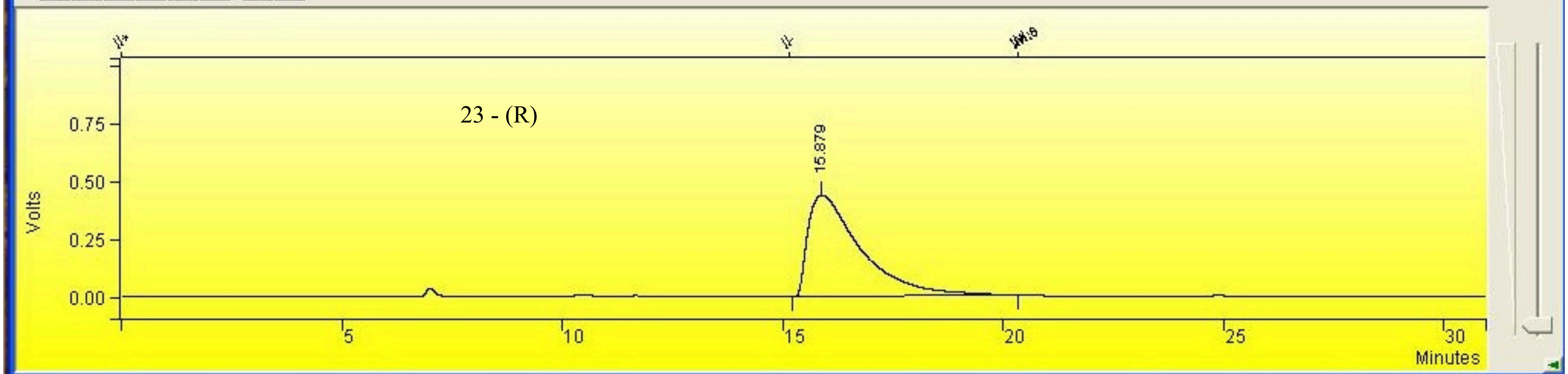

Results

```
Injection Date: 8/19/2013 10:49 AM    Calculation Date: 9/17/2013 5:27 PM

Operator    : Operator               Detector Type: 0800 (1 Volt)
Workstation: TROST-HPLC-LEFÿ□HÚþ˜□  Bus Address  : 80
Instrument : Instrument #1           Sample Rate  : 50.00 Hz
Channel    : 1 = 1                   Run Time     : 30.992 min

** LC Workstation Multi Instrument (Demo) Version 6.41 ** 05000-31c8-fa9-30a1 **

Run Mode          : Analysis
Peak Measurement: Peak Area
Calculation Type: Percent

                                    Ret.    Time                      Width
 Peak      Peak          Result     Time    Offset      Area     Sep.  1/2    Status
  No.      Name           ()        (min)   (min)     (counts)   Code (sec)   Codes
 ---- ------------ ---------- ------- ------- ---------- ---- ----- ------
    1                100.0000  15.879   0.000   36512908   BB  92.0
 ---- ------------ ========== ------- ======= ========== ---- ----- ------
```

[illegible]

This journal is © The Royal Society of Chemistry 2014

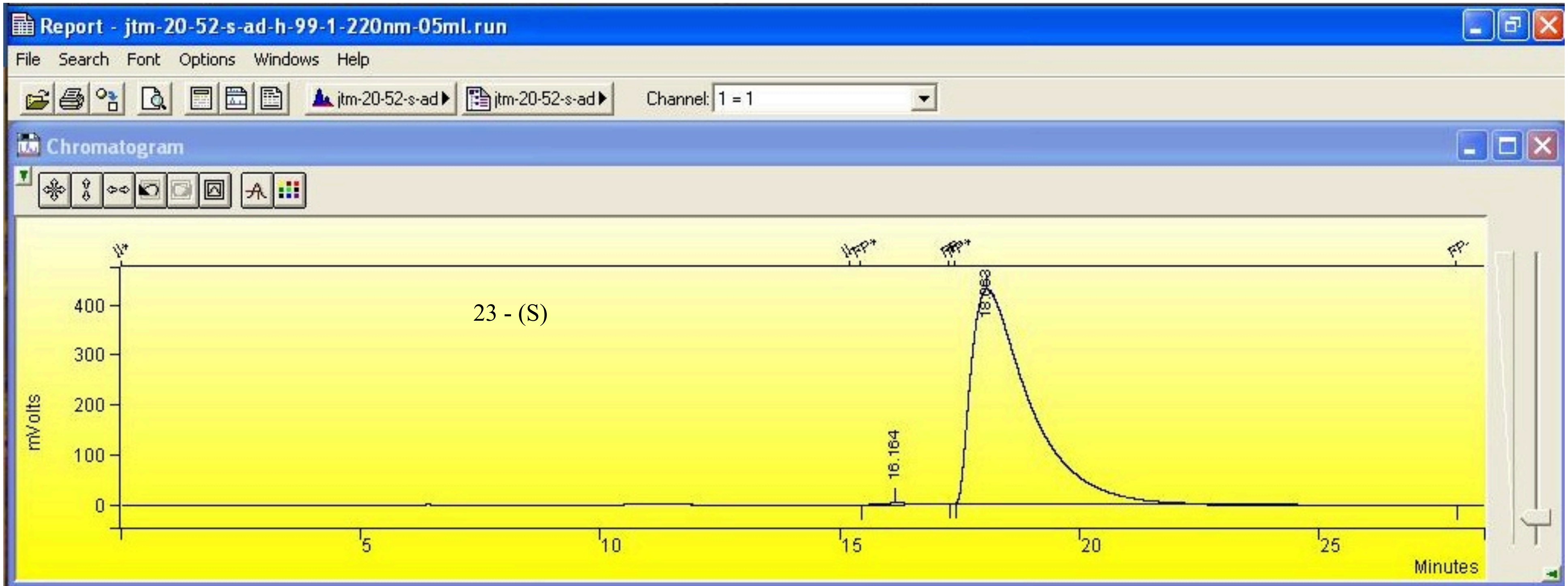

Results

```
Operator   : Operator             Detector Type: 0800 (1 Volt)
Workstation: TROST-HPLC-LEFÿ□HÚþ`  Bus Address  : 80
Instrument : Instrument #1         Sample Rate  : 50.00 Hz
Channel    : 1 = 1                 Run Time     : 28.496 min

** LC Workstation Multi Instrument (Demo) Version 6.41 ** 05000-31c8-fa9-30a1 **

Run Mode         : Analysis
Peak Measurement: Peak Area
Calculation Type: Percent
```

Peak No.	Peak Name	Result ()	Ret. Time (min)	Time Offset (min)	Area (counts)	Sep. Code	Width 1/2 (sec)	Status Codes
1		0.5518	16.164	0.000	200375	BB	70.9	
2		99.4482	18.063	0.000	36110976	BB	73.3	
[illegible]		[illegible]		[illegible]	[illegible]			

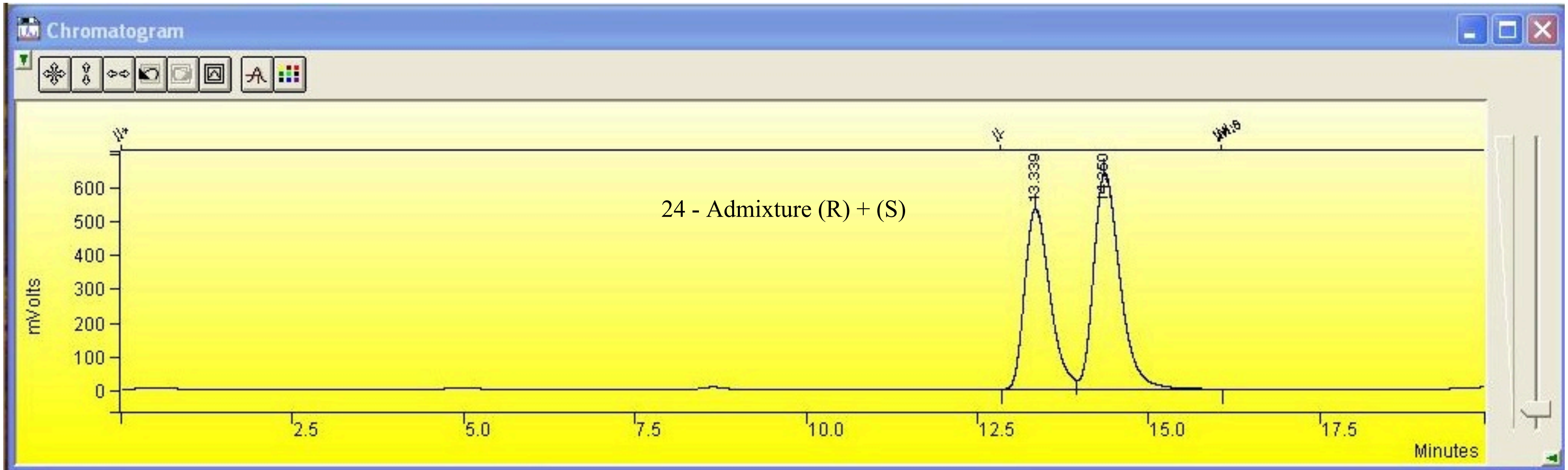

Results

Injection Date: 9/5/2013 11:46 AM   Calculation Date: 9/5/2013 12:54 PM

Operator : Operator   Detector Type: 0800 (1 Volt)
Workstation: TROST-HPLC-LEFÿ□HÚþ`   Bus Address : 80
Instrument : Instrument #1   Sample Rate : 50.00 Hz
Channel : 1 = 1   Run Time : 19.930 min

** LC Workstation Multi Instrument (Demo) Version 6.41 ** 05000-31c8-fa9-30a1 **

Run Mode : Analysis
Peak Measurement: Peak Area
Calculation Type: Percent

Peak No.	Peak Name	Result ()	Ret. Time (min)	Time Offset (min)	Area (counts)	Sep. Code	Width 1/2 (sec)	Status Codes
1		44.3609	13.339	0.000	13760318	BV	24.0	
2		55.6391	14.350	0.000	17258698	VB	24.4	

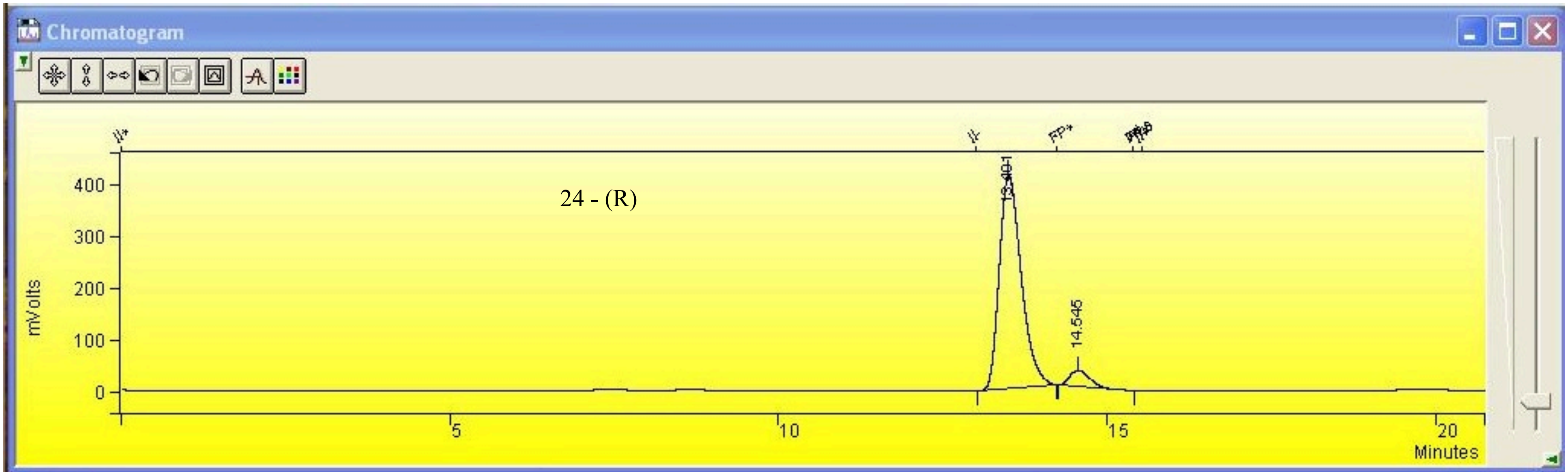

24 - (R)

Results

```
Workstation: TROST-HPLC-LEFÿ□HÚþ˜□  Bus Address  : 80
Instrument : Instrument #1          Sample Rate  : 50.00 Hz
Channel    : 1 = 1                  Run Time     : 20.767 min

** LC Workstation Multi Instrument (Demo) Version 6.41 ** 05000-31c8-fa9-30a1 **

Run Mode          : Analysis
Peak Measurement: Peak Area
Calculation Type: Percent
```

Peak No.	Peak Name	Result ()	Ret. Time (min)	Time Offset (min)	Area (counts)	Sep. Code	Width 1/2 (sec)	Status Codes
1		93.6570	13.491	0.000	9625068	BB	21.5	
2		6.3430	14.545	0.000	651871	BB	19.0	
	Totals:	100.0000		0.000	10276939			

[illegible]

Report - jtm-20-86-s-adh-98-02-03ml-220nm.run

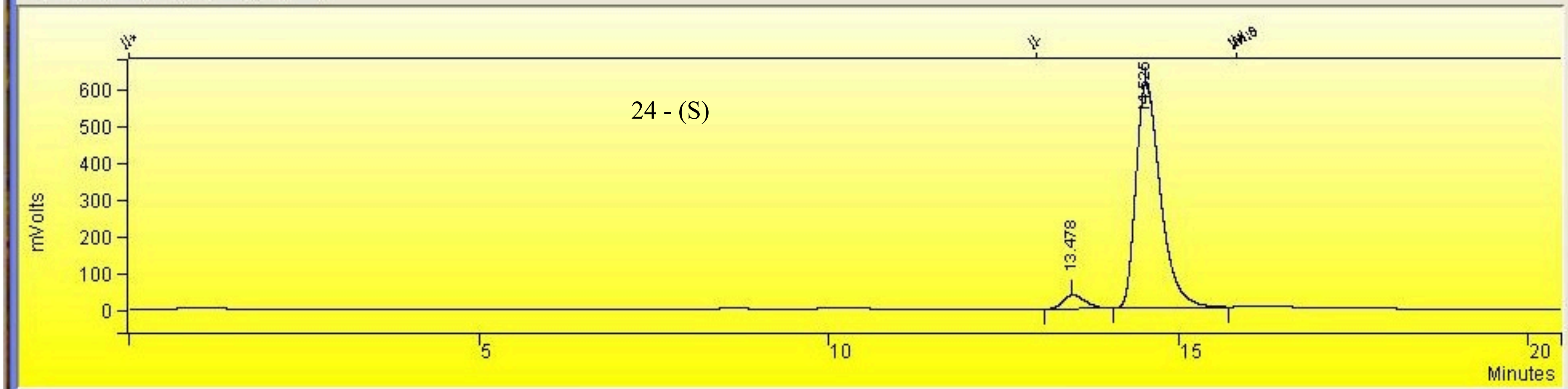

Results

```
Operator    : Operator                Detector Type: 0800 (1 Volt)
Workstation: TROST-HPLC-LEFÿ□HÚþ`     Bus Address  : 80
Instrument : Instrument #1            Sample Rate  : 50.00 Hz
Channel     : 1 = 1                   Run Time     : 20.493 min

** LC Workstation Multi Instrument (Demo) Version 6.41 ** 05000-31c8-fa9-30a1 **

Run Mode          : Analysis
Peak Measurement: Peak Area
Calculation Type: Percent
```

Peak No.	Peak Name	Result ()	Ret. Time (min)	Time Offset (min)	Area (counts)	Sep. Code	Width 1/2 (sec)	Status Codes
1		5.2388	13.478	0.000	839303	BP	21.3	
2		94.7611	14.525	0.000	15181440	PB	22.2	
[illegible]		[illegible]		[illegible]	[illegible]			

ple Name: jtm-21-31-dppf-rac

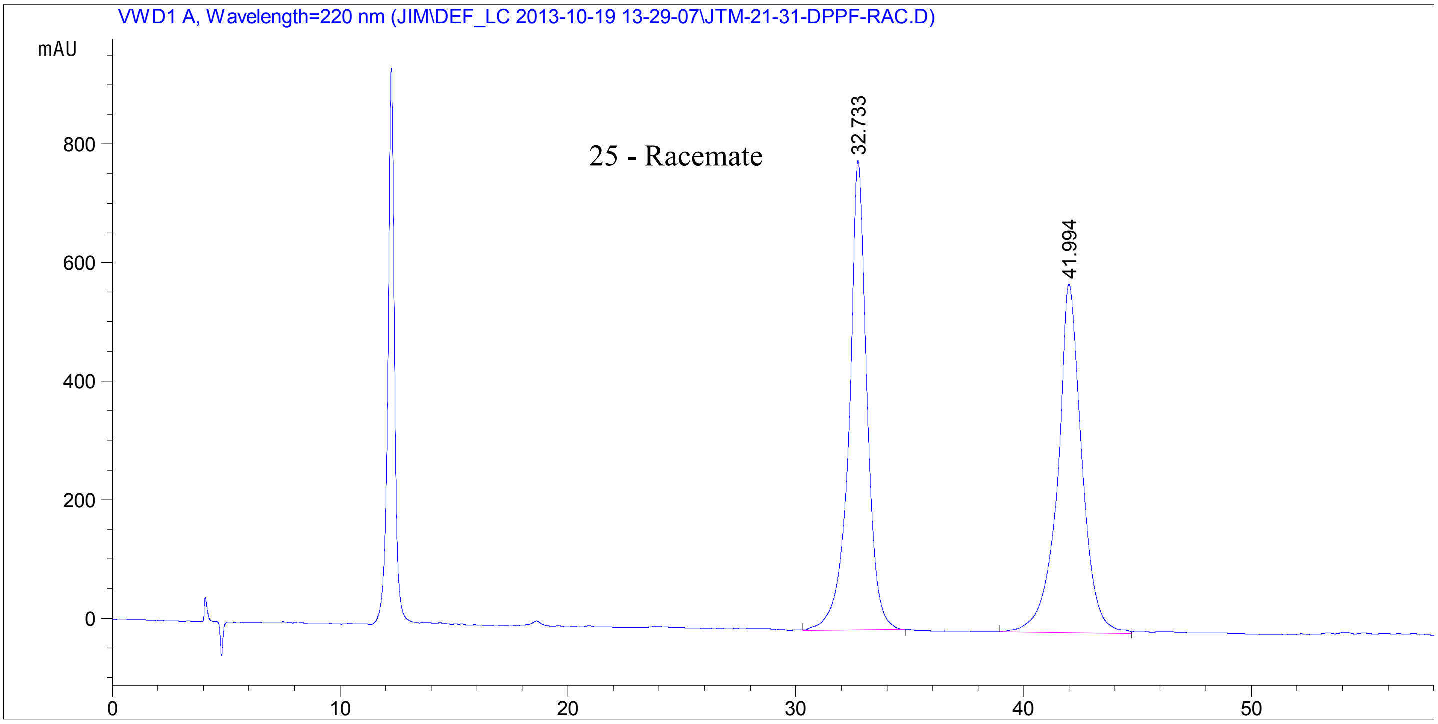

Area Percent Report

Sorted By : Signal
Multiplier: : 1.0000
Dilution: : 1.0000
Use Multiplier & Dilution Factor with ISTDs

Signal 1: VWD1 A, Wavelength=220 nm

Peak #	RetTime [min]	Type	Width [min]	Area mAU *s	Height [mAU]	Area %
1	32.733	VB	0.7858	4.33353e4	791.51624	49.8124
2	41.994	VV	1.0552	4.36618e4	588.27252	50.1876

Totals : 8.69971e4 1379.78876

*** End of Report ***

ple Name: jtm-21-33-r

Method Method stopped by user 17:28:08 10/21/13

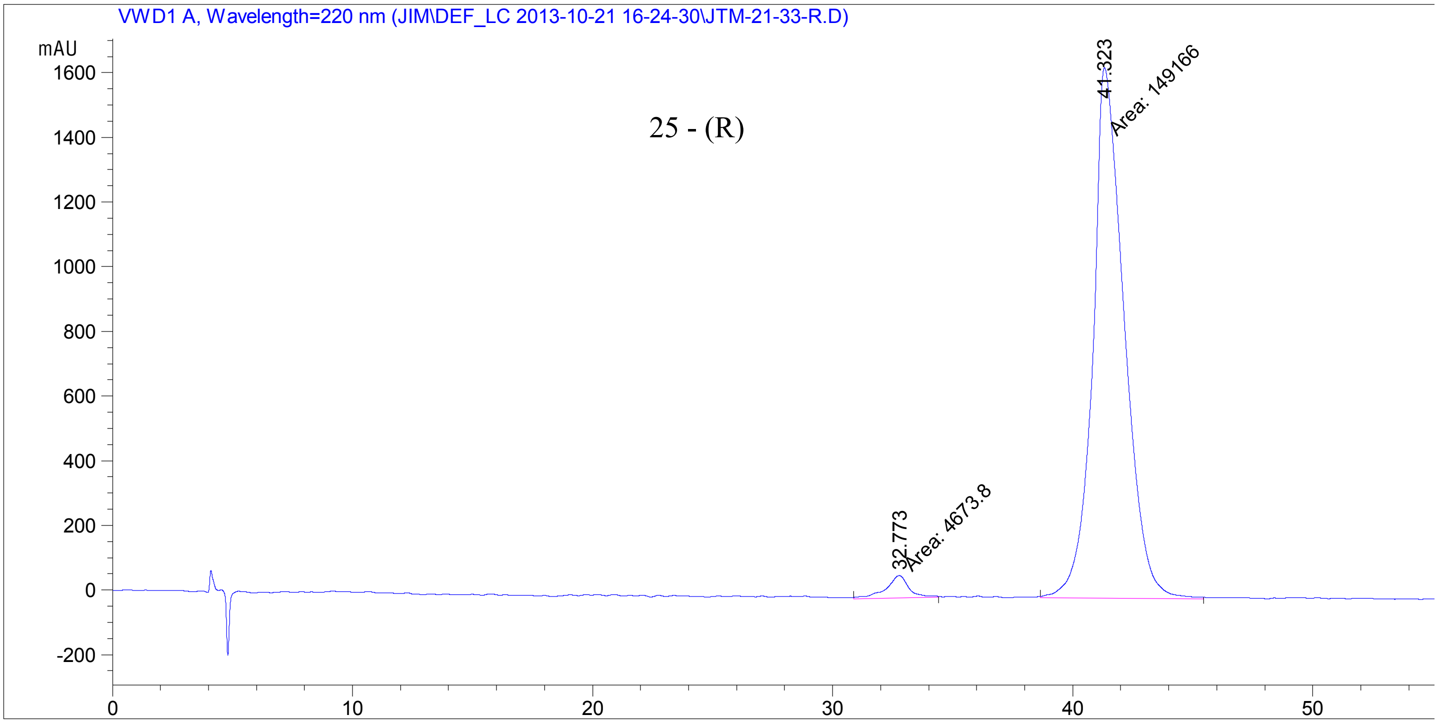

Area Percent Report

Sorted By : Signal
Multiplier: : 1.0000
Dilution: : 1.0000
Use Multiplier & Dilution Factor with ISTDs

Signal 1: VWD1 A, Wavelength=220 nm

Peak #	RetTime [min]	Type	Width [min]	Area mAU *s	Height [mAU]	Area %
1	32.773	MM	1.1269	4673.79688	69.12724	3.0381
2	41.323	MM	1.5156	1.49166e5	1640.31873	96.9619

Totals : 1.53840e5 1709.44596

*** End of Report ***

Electronic Supplementary Material (ESI) for Chemical Science
This journal is © The Royal Society of Chemistry 2014

Report - jtm-20-89-no-cyclized-rac-95-5-adh-08-220.run

File Search Font Options Windows Help

Channel: 1 = 1

Chromatogram

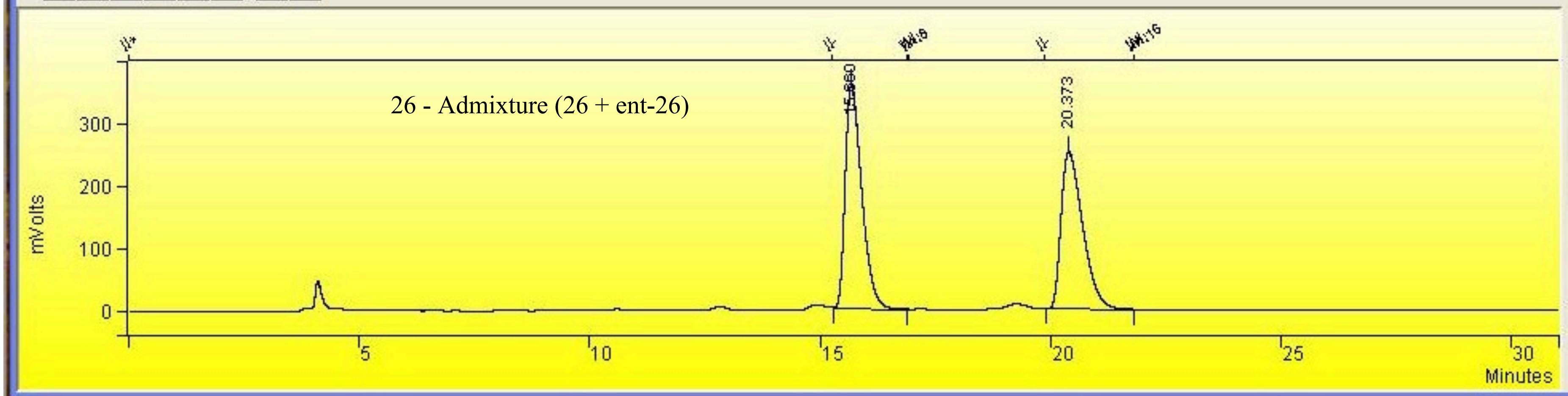

Results

```
Operator   : Operator              Detector Type: 0800 (1 Volt)
Workstation: TROST-HPLC-LEFÿ□HÚþ`  Bus Address  : 80
Instrument : Instrument #1         Sample Rate  : 50.00 Hz
Channel    : 1 = 1                 Run Time     : 31.057 min

** LC Workstation Multi Instrument (Demo) Version 6.41 ** 05000-31c8-fa9-30a1 **

Run Mode         : Analysis
Peak Measurement: Peak Area
Calculation Type: Percent
```

Peak No.	Peak Name	Result ()	Ret. Time (min)	Time Offset (min)	Area (counts)	Sep. Code	Width 1/2 (sec)	Status Codes
1		51.8622	15.660	0.000	8889672	BB	22.8	
2		48.1378	20.373	0.000	8251278	BB	30.2	
	Totals:	100.0000		0.000	17140950			

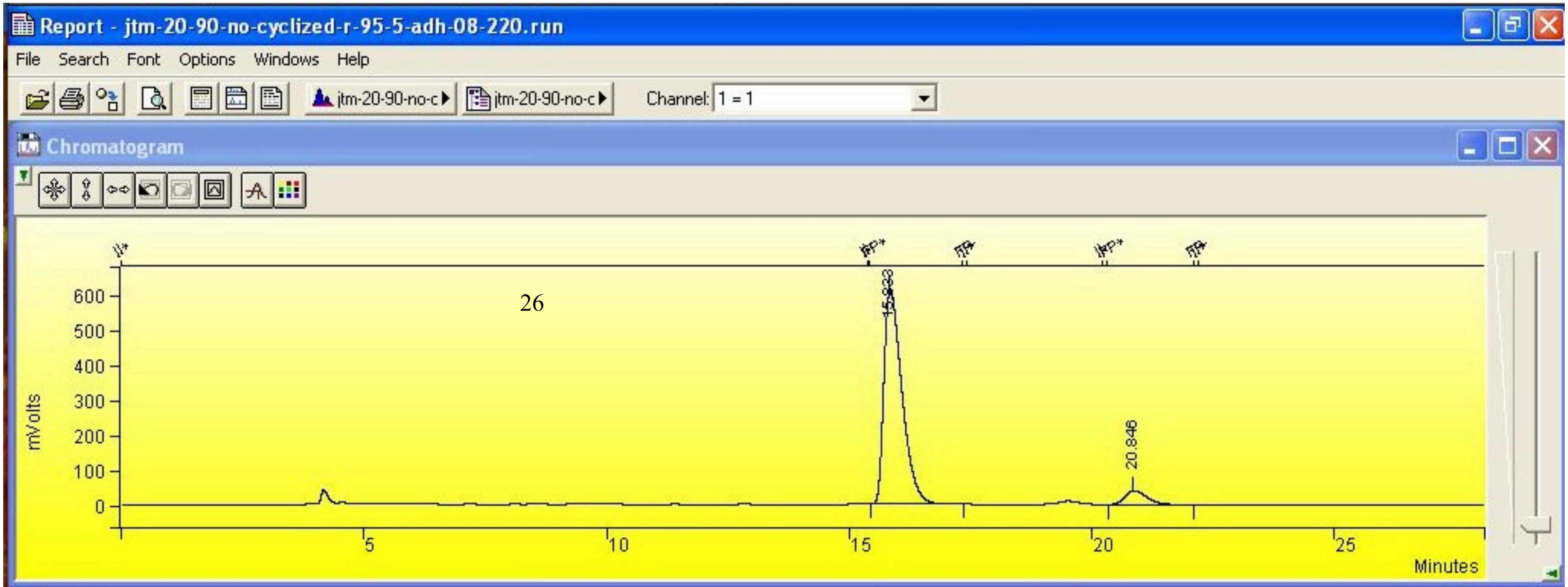

26

Results

```
Operator   : Operator                Detector Type: 0800 (1 Volt)
Workstation: TROST-HPLC-LEFÿ□HÚþ`    Bus Address  : 80
Instrument : Instrument #1           Sample Rate  : 50.00 Hz
Channel    : 1 = 1                   Run Time     : 28.123 min

** LC Workstation Multi Instrument (Demo) Version 6.41 ** 05000-31c8-fa9-30a1 **

Run Mode         : Analysis
Peak Measurement: Peak Area
Calculation Type: Percent
```

Peak No.	Peak Name	Result ()	Ret. Time (min)	Time Offset (min)	Area (counts)	Sep. Code	Width 1/2 (sec)	Status Codes
1		92.5793	15.833	0.000	15950677	BB	23.7	
2		7.4207	20.846	0.000	1278535	BB	29.1	
Totals:		[illegible]		[illegible]	[illegible]			

Report - jtm-20-89-no-cyclized-s-95-5-adh-08-220.run

File Search Font Options Windows Help

Channel: 1 = 1

Chromatogram

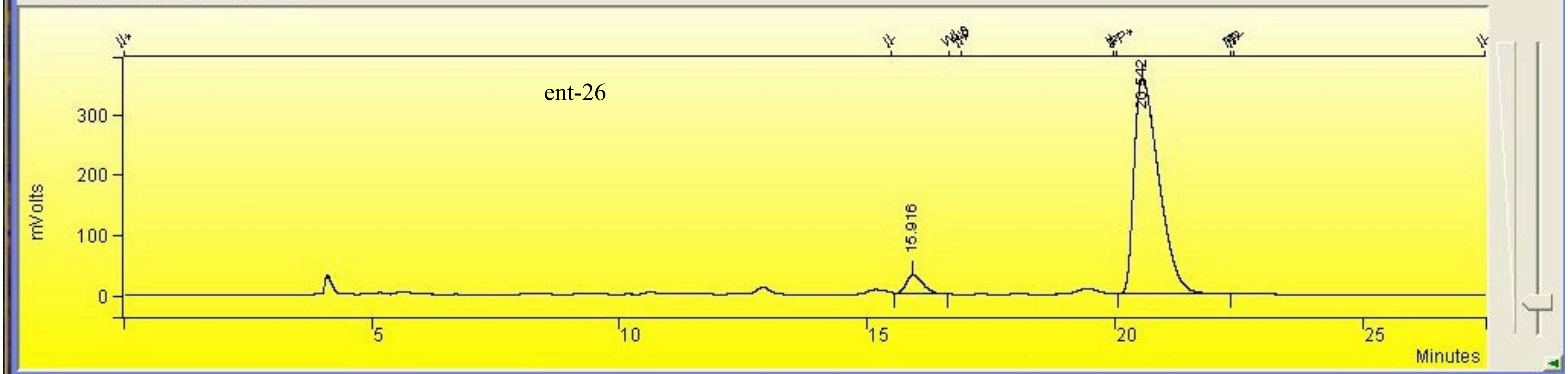

Results

```
Operator   : Operator              Detector Type: 0800 (1 Volt)
Workstation: TROST-HPLC-LEFÿ□HÚþ`□ Bus Address  : 80
Instrument : Instrument #1         Sample Rate  : 50.00 Hz
Channel    : 1 = 1                 Run Time     : 27.527 min

** LC Workstation Multi Instrument (Demo) Version 6.41 ** 05000-31c8-fa9-30a1 **

Run Mode        : Analysis
Peak Measurement: Peak Area
Calculation Type: Percent
```

Peak No.	Peak Name	Result ()	Ret. Time (min)	Time Offset (min)	Area (counts)	Sep. Code	Width 1/2 (sec)	Status Codes
1		5.2966	15.916	0.000	694248	BB	21.1	
2		94.7034	20.542	0.000	12413209	BB	32.6	
	Totals:	100.0000		0.000	13107457			

Report - jtm-epoxybenzoate-oj-h-90-10-08-220nm-run2.run

File Search Font Options Windows Help

Channel: 1 = 1

Chromatogram

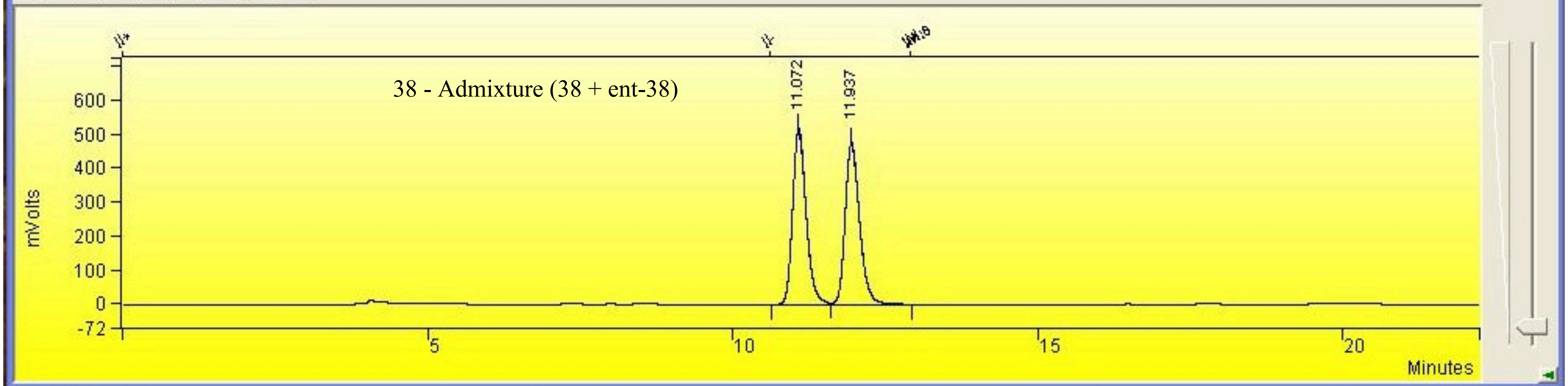

Results

```
Operator   : Operator                Detector Type: 0800 (1 Volt)
Workstation: TROST-HPLC-LEFÿ□HÚp`    Bus Address  : 80
Instrument : Instrument #1           Sample Rate  : 50.00 Hz
Channel    : 1 = 1                   Run Time     : 22.272 min

** LC Workstation Multi Instrument (Demo) Version 6.41 ** 05000-31c8-fa9-30a1 **

Run Mode         : Analysis
Peak Measurement: Peak Area
Calculation Type: Percent

                                Ret.    Time                       Width
Peak      Peak        Result    Time   Offset      Area     Sep.   1/2    Status
 No.      Name         ()       (min)   (min)    (counts)   Code  (sec)   Codes
---- ------------ ----------  ------- ------- ----------  ---- -----   ------
   1                 50.5956   11.072   0.000    8061113    BV  13.9
   2                 49.4044   11.937   0.000    7871338    VB  14.9
---- ------------ ==========  ------- ======= ==========  ---- -----   ------
      Totals:       100.0000            0.000   15932451
```

Electronic Supplementary Material (ESI) for Chemical Science
This journal is © The Royal Society of Chemistry 2014

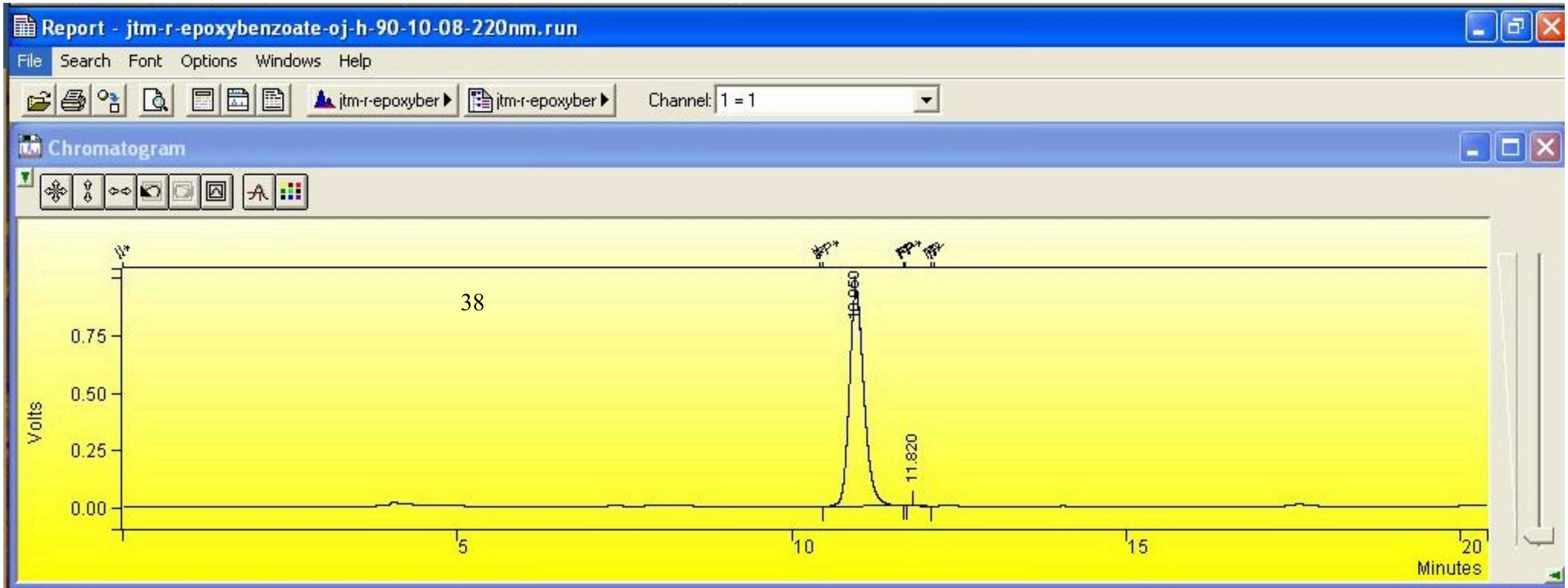

Results

Injection Date: 8/20/2013 10:56 AM   Calculation Date: 8/20/2013 11:20 AM

Operator : Operator	Detector Type: 0800 (1 Volt)
Workstation: TROST-HPLC-LEFÿ□HÚþ`□	Bus Address : 80
Instrument : Instrument #1	Sample Rate : 50.00 Hz
Channel : 1 = 1	Run Time : 20.427 min

** LC Workstation Multi Instrument (Demo) Version 6.41 ** 05000-31c8-fa9-30a1 **

Run Mode : Analysis
Peak Measurement: Peak Area
Calculation Type: Percent

Peak No.	Peak Name	Result ()	Ret. Time (min)	Time Offset (min)	Area (counts)	Sep. Code	Width 1/2 (sec)	Status Codes
1		99.9278	10.950	0.000	14538395	BB	13.8	
2		0.0722	11.820	0.000	10497	BB	7.5	

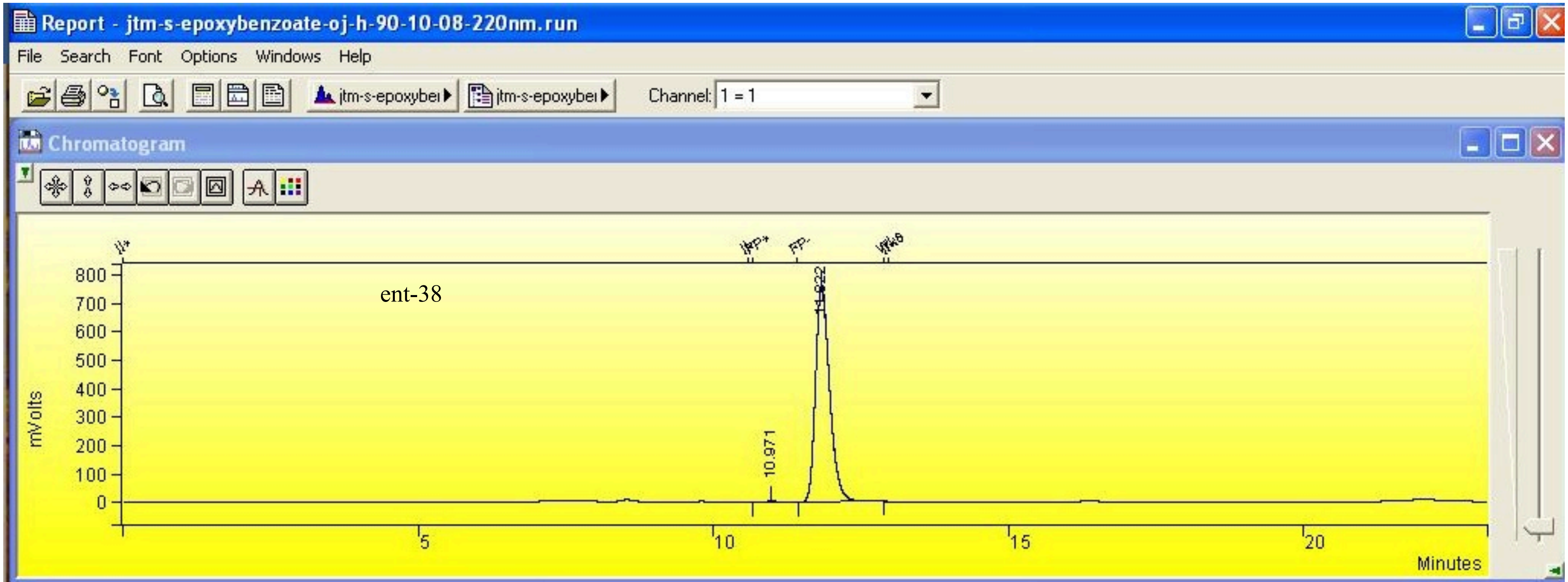

Results

```
Operator   : Operator              Detector Type: 0800 (1 Volt)
Workstation: TROST-HPLC-LEFÿ□HÚþ``  Bus Address  : 80
Instrument : Instrument #1         Sample Rate  : 50.00 Hz
Channel    : 1 = 1                 Run Time     : 23.141 min

** LC Workstation Multi Instrument (Demo) Version 6.41 ** 05000-31c8-fa9-30a1 **

Run Mode         : Analysis
Peak Measurement: Peak Area
Calculation Type: Percent
```

Peak No.	Peak Name	Result ()	Ret. Time (min)	Time Offset (min)	Area (counts)	Sep. Code	Width 1/2 (sec)	Status Codes
1		0.2729	10.971	0.000	34732	BB	13.9	
2		99.7271	11.822	0.000	12690070	BB	15.1	
